# Redox‐Neutrale Selen‐katalysierte Isomerisierung von *para*‐Hydroxamsäuren zu *para*‐Aminophenolen

**DOI:** 10.1002/ange.202100801

**Published:** 2021-03-24

**Authors:** Hsiang‐Yu Chuang, Manuel Schupp, Ricardo Meyrelles, Boris Maryasin, Nuno Maulide

**Affiliations:** ^1^ Universität Wien Institut für Organische Chemie Währinger Strasse 38 1090 Wien Österreich; ^2^ CeMM – Forschungszentrum für Molekulare Medizin der Österreichischen Akademie der Wissenschaften Lazarettgasse 14, AKH BT 25.3 1090 Wien Österreich; ^3^ Universität Wien Institut für Theoretische Chemie Währinger Straße 17 1090 Wien Österreich

**Keywords:** [2,3]-Umlagerung, Aminophenol, Hydroxamsäure, N-O-Bindungsbruch, Selen

## Abstract

Über eine Selen‐katalysierte *para*‐Hydroxylierung von *N*‐Arylhydroxamsäuren wird berichtet. Mechanistisch verläuft diese über N‐O‐Bindungsbruch und nachfolgende Selen‐induzierte [2,3]‐Umlagerungen um *para*‐Hydroxylanilinderivate zu erzeugen. Der Mechanismus wurde sowohl mittels ^18^O‐Überkreuzungsexperimenten als auch quantenchemischen Berechnungen untersucht. Diese redox‐neutrale Transformation ermöglicht einen ungewöhnlichen synthetischen Zugang zu *para*‐Aminophenolen.

## Graphical abstract

Spaß im Doppelpack: Eine Organoselen‐katalysierte, regiospezifische *para*‐Hydroxylierung ausgehend von *N*‐Arylhydroxamsäuren mittels doppelter [2,3]‐sigmatroper Umlagerung wurde entwickelt. Diese operativ einfache Reaktion erzeugt nützliche *para*‐Aminophenolbausteine in kleinem und großem Maßstab und der zugrundeliegende Mechanismus wird mittels quantenchemischer Berechnungen als auch Markierungsexperimenten überprüft.

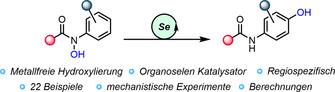

Das *para*‐Aminophenolmotiv, dessen Inbegriff das jahrhundertalte Analgetikum Paracetamol darstellt, ist ein wichtiges Strukturmotiv in pharmazeutischen Substanzen und Materialen. Zahlreiche Methoden zur Darstellung von *para*‐Aminophenolen wurden seit der Entdeckung der ersten praktikablen Synthesemethode durch Eugen Bamberger entdeckt, welcher *para*‐Aminophenol durch die Umlagerung von *N*‐Arylhydroxylaminen in wässriger Schwefelsäure herstellte (Schema 1 a).^[1]^ Dieser Prozess verläuft vermutlich über die heterolytische Spaltung der N‐O‐Bindung und nachfolgender intermolekularer Addition von Wasser an ein Nitreniumion. Neben starken Brønsted‐Säuren können diese N‐O‐Bindungsbrüche/Umlagerungssequenzen auch mittels Lewis‐Säuren,^[2]^ thermischer Aktivierung^[3]^ oder Übergangsmetallen erreicht werden.^[4]^ Pionierarbeit durch Nutzung einer Lewis‐Säure zur säure‐vermittelten *ortho*‐Migration einer Methoxygruppe wurde von Kikugawa geleistet (Schema 1 b).^[2]^ Später stellte die gleiche Arbeitsgruppe die PBu_3_/CCl_4_‐induzierte *ortho*‐Migration von Hydroxylgruppen in *N*‐Aryl‐*N*‐phenylhydroxylaminen vor (Schema 1 c), wobei auch die entsprechenden *para*‐Isomere als Nebenprodukte gefunden wurden.^[5]^ Ngai zeigte hingegen eine elegante *ortho*‐Trifluoromethoxylierung von Anilinen durch einen thermischen Umlagerungsprozess (Schema 1 d).^[3]^ Vor kurzem berichtete zudem Terada in einer detaillierten Studie über eine Kobalt‐katalysierte [1,3]‐Migration von Alkoxycarbonyloxygruppen (Schema 1 e).^[5]^ Interessanterweise verlaufen die meisten dieser N‐O‐Bindungsbruchprozesse unter Bildung neuer C‐O‐Bindungen mit *ortho*‐Selektivität. Die wenigen vorhandenen Methoden zur *para*‐Hydroxylierung benötigen entweder verhältnismäßig harsche Bedingungen oder erzeugen Produktgemische aus *ortho*‐ und *para*‐Regioisomeren.[^5^, ^6^] Unserem Wissen nach ist bisher keine milde und praktikable Methode zur regioselektiven *para*‐Hydroxylierung vorgestellt worden.^[7]^

**Scheme 1:**
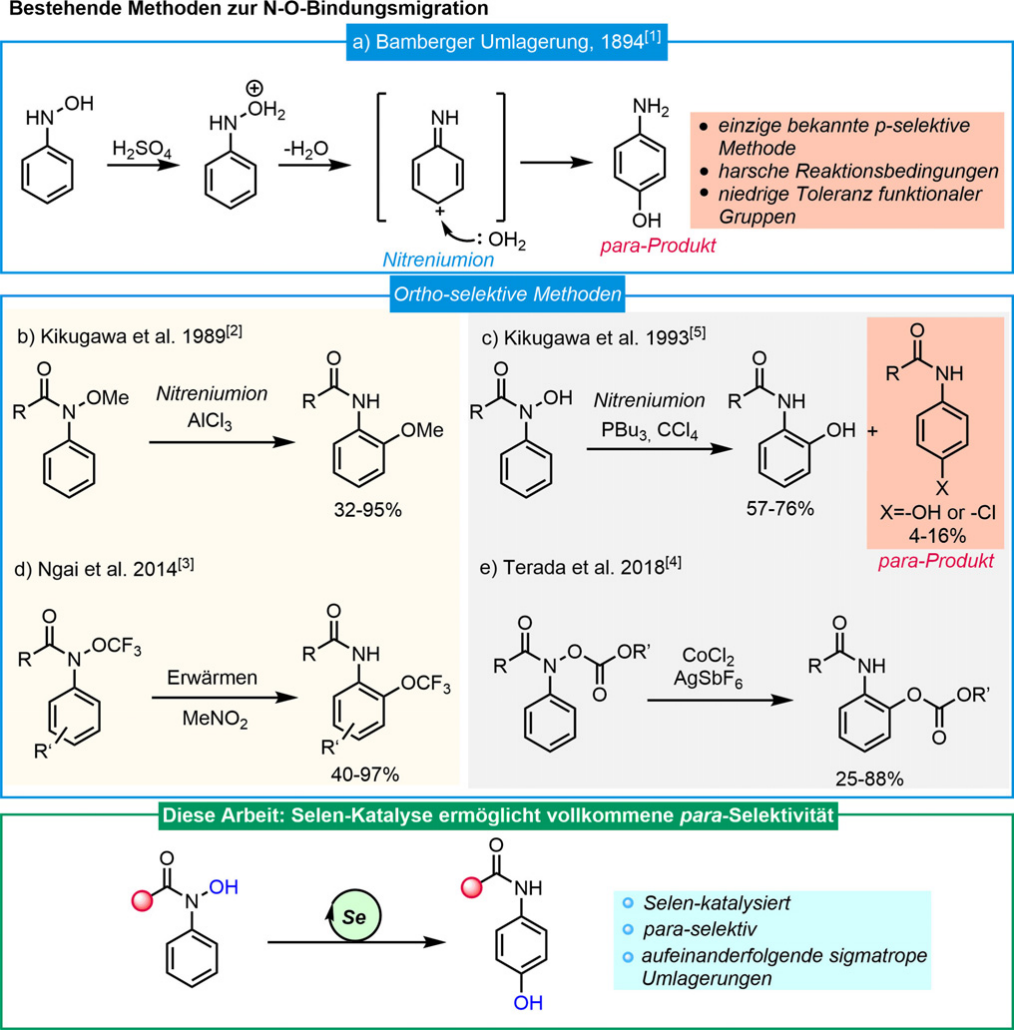
Zugänge zum N‐O‐Bindungsbruch/Sauerstoffmigrationsreaktionen und die hier vorgestellte Arbeit.

Selen ist ein essenzielles Oligoelement, welches vermutlich am besten für sein Vorkommen in Selenocystein bekannt ist.[^8^, ^9^, ^10^] Innerhalb der organischen Synthese haben sich Organoselenverbindungen als einzigartige Katalysatoren für Oxidationen,^[11]^ Reduktionen,^[12]^ C‐C/C‐X‐Bindungsbildung und Umlagerungen etabliert.[^13^, ^14^, ^15^] Das schwerere Selen besitzt einzigartige Eigenschaften im Vergleich zu den anderen Chalkogenen.^[16]^ Hier möchten wir nun eine neue Selen‐katalysierte *para*‐selektive Hydroxylierung ausgehend von Hydroxamsäuren über aufeinanderfolgende [2,3]‐Umlagerungen vorstellen (Schema 1 f).

In ersten Versuchen versetzen wir die Hydroxamsäure **1** mit einem Äquivalent PhSeBr. Zu unserer Zufriedenstellung wurde *para*‐Aminophenol **2** mit 72 % isolierter Ausbeute erhalten (Tabelle 1, Eintrag 1). Durch dieses frühe Resultat ermutigt stellten wir fest, dass durch das Reduzieren des Einsatzes von Phenylselenylbromid auf 10 mol % immer noch das *para*‐Aminophenol **2** mit zunächst 66 % isolierter Ausbeute erhalten wurde (Eintrag 2). Es ist anzumerken, dass dieser katalytische Prozess, wenngleich er auch längere Reaktionszeiten erfordert um vollen Umsatz zu erreichen, dennoch nur mit geringen Einbußen bei der Ausbeute verläuft. Wir stellten zudem fest, dass die *para*‐Hydroxylierung mittels PhSeCl‐Katalyse fast identische Ausbeute lieferte (Eintrag 3). Ein Wechsel des Katalysators zu *N*‐(phenylselenyl)‐phthalimid oder 2‐Nitrophenylselenocyanat führte zu Ausbeuten von 40 % und 35 % des *para*‐Aminophenols **2** (Einträge 4 und 5). Um die Elektrophilie des Selenreagenz zu erhöhen wurde eine Kombination von PhSeCl und AgOTf versucht, welche allerdings nur eine Ausbeute von **2** von 25 % erzielen konnte (Eintrag 6). PhSeSePh war ineffektiv als Katalysator und das Startmaterial konnte reisoliert werden. Nach diesen anfänglichen Beobachtungen wurde Phenylselenylbromid als Katalysator für weitere Experimente ausgewählt. In nachfolgenden Versuchen wurden verschiedene Lösungsmittel untersucht. Dichlormethan, Acetonitril und verschiedene Ether konnten erfolgreich in der Reaktion eingesetzt werden (Tabelle 1, Einträge 8–12). 1,4‐Dioxan wurde letztlich als ideales Lösungsmittel gewählt, da der verhältnismäßig hohe Siedepunkt größere Flexibilität beim Erhitzen problematischer Substrate ermöglicht (vide infra).

**Table 1:** Untersuchung der Selen‐Katalysatoren. [a] Reaktionen wurden mit einer Konzentration von 0.2 M durchgeführt. [b] Ausbeuten wurden mittels NMR bestimmt unter Einsatz von Trimethoxybenzol als internem Standard. [c] Isolierte Ausbeuten.

Eintrag	Reagenz	Lösungsmittel^[a]^	Temperatur	Zeit	Ausbeute^[b]^
1	PhSeBr (1 Äquiv.)	1,4‐Dioxan	rt	1 h	72 %^c^
2	PhSeBr (10 mol %)	1,4‐Dioxan	rt	3 h	66 %
3	PhSeCl (10 mol %)	1,4‐Dioxan	rt	6 h	67 %
4	*N*‐(Phenylseleno)‐phthalimid (10 mol %)	1,4‐Dioxan	rt	12 h	40 %
5	2‐Nitrophenylselenocyanat (10 mol %)	1,4‐Dioxan	rt	18 h	35 %
6	PhSeCl (10 mol %) AgOTf (10 mol %)	1,4‐Dioxan	rt	18 h	25 %
7	PhSeSePh (10 mol %)	1,4‐Dioxan	rt	18 h	0 %
8	PhSeBr (10 mol %)	1,4‐Dioxan	rt	3 h	79 % (76 %)^c^
9	PhSeBr (10 mol %)	MeOH	rt	3 h	29 %
10	PhSeBr (10 mol %)	CH_2_Cl_2_	rt	3 h	73 %
11	PhSeBr (10 mol %)	MeCN	rt	3 h	76 %
12	PhSeBr (10 mol %)	THF	rt	3 h	81 %

Nachdem bestmögliche Reaktionsbedingungen gefunden wurden wendeten wir unsere Aufmerksamkeit der Reichweite der vorgestellten Selen‐katalysierten *para*‐Hydroxylierung zu (Schema 2). Wie gezeigt wird, toleriert diese Transformation eine außerordentliche Breite an funktionellen Gruppen, wie unter anderem die sterische gehinderten Pivalamide **4 a** und Adamantylamide **4 b**, als auch das stark gespannte Cyclobutylamid **4 c**. Bemerkenswerterweise wurden höhere Ausbeuten und kürzere Reaktionszeiten bei Substraten, welche elektronenziehende Benzamidfragmente tragen, erzielt (**4 d**, **2**, **4 e**). Dies scheint mit den verhältnismäßig schwächeren N‐O‐Bindungen dieser Substrate zusammenzuhängen. Zudem werden auch das Zimtamid **4 f**, als auch das Styrolamid **4 g** toleriert, allerdings mit leicht verringerten Ausbeuten. Nachfolgenden wurde eine Auswahl verschiedener Substituenten am *N*‐Arylring untersucht. Das Naphthalinderivat **3 i** reagierte reibungslos zum Aminophenol **4 i**. Außerdem sind das gehinderte 3,4‐dimethylsubstituierte Substrat **3 k** und das 2,6‐dimethylsubstituierte Substrat **3 l** hervorzuheben, welche beide das entsprechende *para*‐Aminophenol bildeten. Zusätzlich lässt sich unsere Reaktionsführung problemlos auf halogenierte Substrate anwenden, welche die gewünschten *para*‐Aminophenole bildeten (**4 o**–**4 t**). Elektronische Effekte am *N*‐Arylring beeinflussen die Ausbeuten erheblich: während das elektronenreiche 4‐Methoxyphenylsubstrat **3 m** eine hohe Ausbeute bei Raumtemperatur liefert müssen die *N*‐elektronenarmen Hydroxamsäuren (**4 n, 4 p, 4 q**) bei erhöhten Temperaturen umgesetzt werden um die entsprechenden *para*‐Aminophenole in moderaten Ausbeuten zu erhalten.^[17]^

**Scheme 2:**
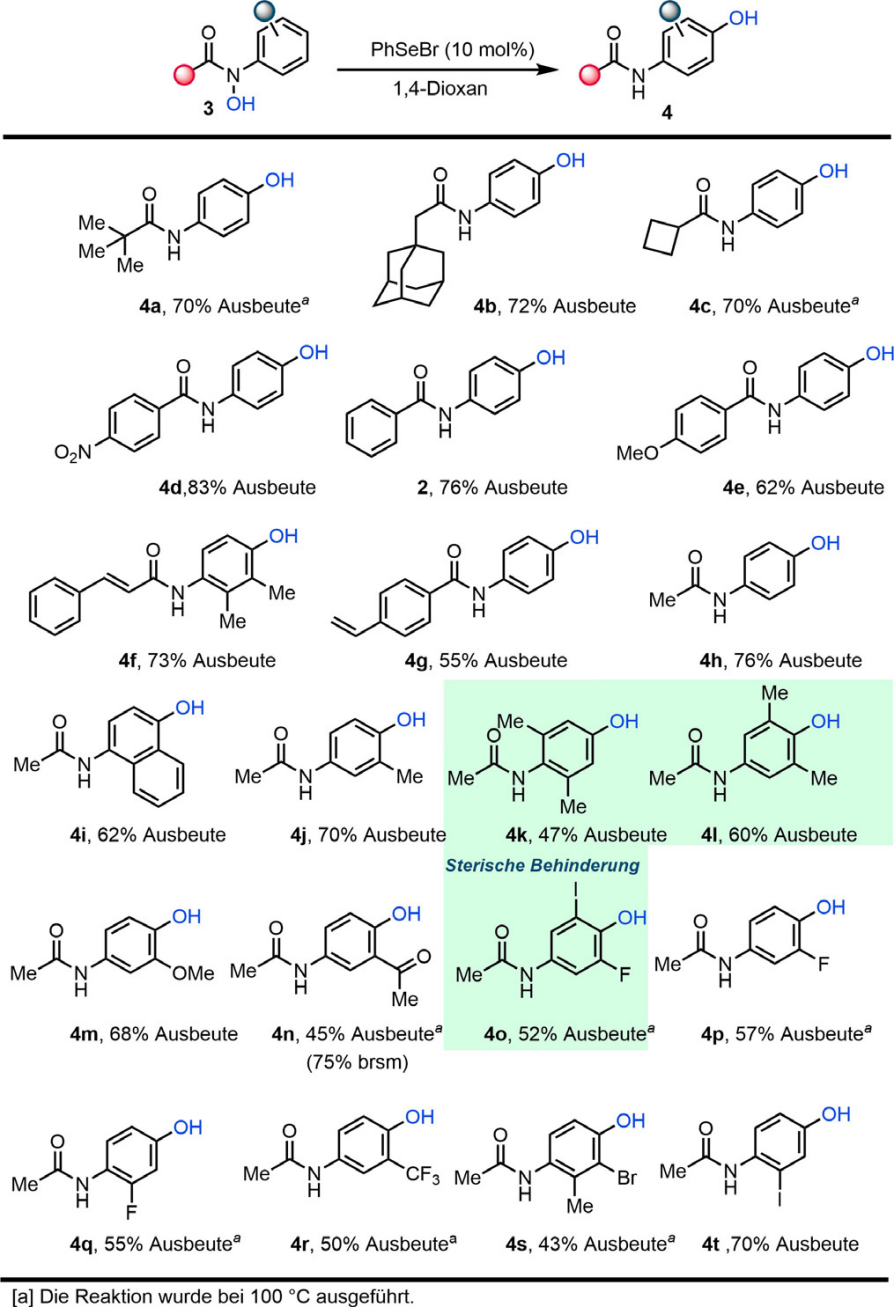
Reichweite der Selen‐Katalysierten *para*‐Hydroxylierung. Ausbeuten beziehen sich auf isolierte, reine Verbindungen.

Um den Mechanismus der vorgestellten Reaktion zu untersuchen wurden nachfolgend ^18^O‐Markierungsexperimente als auch quantenchemische Berechnungen durchgeführt (weitere Informationen in den Hintergrundinformationen). In einem Überkreuzungsexperiment wurden 3 h* und **1** in einem 1:1 Verhältnis unter den optimierten Reaktionsbedingungen umgesetzt, wobei keine Übertragung von ^18^‐O in das Produkt **2** erfolgte (Schema 3). Dieses Ergebnis liefert einen starken Hinweis, dass der vorliegende Prozess *intramolekular* verläuft.

**Scheme 3:**
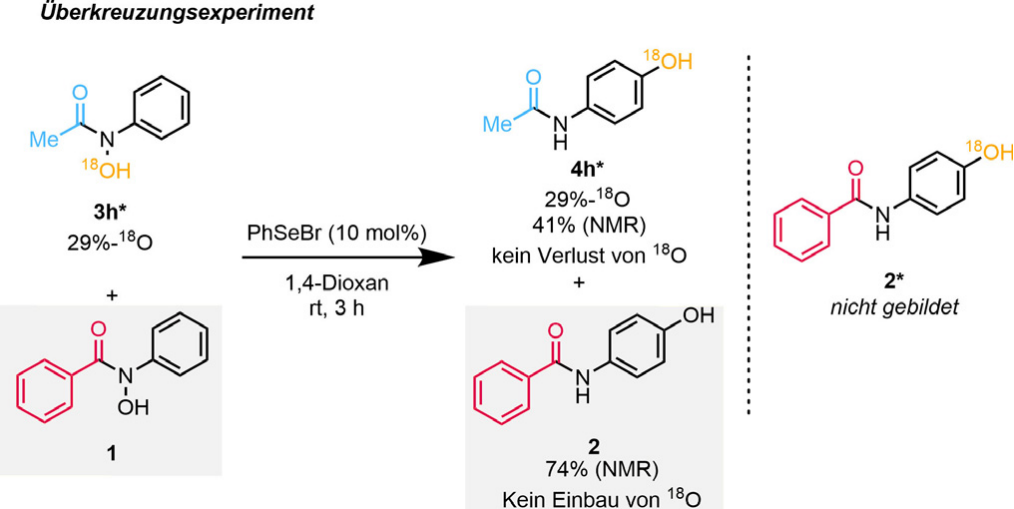
^18^O‐Überkreuzungsexperiment. Die angegebenen Ausbeuten wurden mittels NMR bestimmt unter Einsatz von Trimethoxybenzol als internem Standard.

Quantenchemische Berechnungen wurden durchgeführt, um den Mechanismus der vorliegenden Reaktion zu verstehen. Der berechnete katalytische Zyklus wird in Schema 4 gezeigt (weitere Informationen in den Hintergrundinformationen). Die aktive Spezies **A** wird durch Kombination des Substrates **3 h** mit PhSeBr und internem Protonentransfer erhalten, wie anhand der bekannten Reaktivität von elektrophilen Selenreagenzien zu erwarten ist.^[18]^ Der erste Schritt **A→B** ist eine exergonische [2,3]‐sigmatrope Umlagerung mittels N‐O‐Bindungsbruch und *ortho*‐Angriff von Selen, gefolgt von einem barrierelosen Protonentransfer von **B→C**. Das Intermediat **C** durchläuft nachfolgend einen zweiten Protonentransfer, welcher der zweiten [2,3]‐sigmatropen Umlagerung **D→E** vorausgeht. Dieser Schritt verläuft über eine konzertierte Se‐C‐Bindungsspaltung und Bildung einer neuen C‐O‐Bindung, welche zum *para*‐O‐Arylintermediat **E** führt. Der fünfte Schritt **E→F** ist die äußert thermodynamisch und kinetisch begünstigte (Δ*G*=−28 kcal mol^−1^, Δ*G* ^*≠*^=7 kcal mol^−1^) Rearomatisierung, welche durch ein zweites Substratmolekül unterstützt wird. Der letzte Schritt schließt schlussendlich den katalytischen Zyklus, wobei das Produkt **4 h** gebildet und das Intermediat **A** regeneriert wird. Interessanterweise ist die scheinbare Aktivierungsenergie des Zyklus, Δ*G* ^*≠*^=25 kcal mol^−1^, durch den letzten Schritt, den intermolekularen Protonentransfer, bestimmt.

**Scheme 4:**
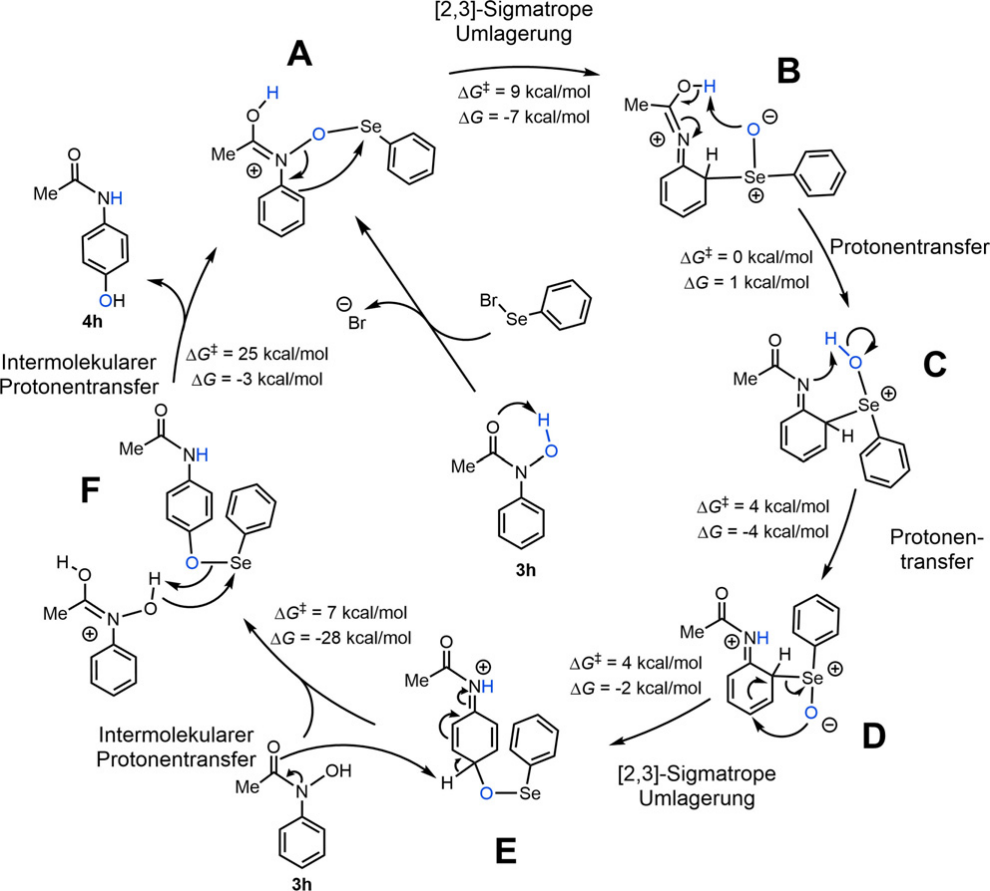
Berechneter katalytische Zyklus mittels PBE0‐D3BJ‐SMD(THF)/def2‐TZVP//PBE0‐D3BJ‐SMD(THF)/def2‐SVP Theorie‐niveau. Die freie Gibbs‐Energien (Δ*G*) und Aktivierungsenergien (Δ*G* ^*≠*^) sind für die jeweiligen Einzelschritte angegeben.

Der vorgeschlagene Mechanismus hebt die kritische Rolle des Substrates bei der Deprotonierung des Intermediates **E** hervor, was im Einklang mit den vorliegenden basenfreien Reaktionsbedingungen ist.

Die vorgestellte redox‐neutrale, regioselektive Hydroxylierung kann zudem in einer Reihe von synthetisch relevanten Bezügen eingesetzt werden (Schema 5). Practolol (Schema 5 a, Verbindung **5**) ist ein bekannter Beta‐Adrenorezeptor‐Antagonist, welcher oft zur Behandlung kardiovaskulärer Krankheiten eingesetzt wird und bereits über verschiedene Routen synthetisiert wurde.[^19^, ^20^] Bei unserer Synthese im Gram‐Maßstab wurde Hydroxamsäure **3 h** der Selen‐katalysierten *para*‐Hydroxylierung unterworfen um das *para*‐Aminophenol **4 h** in 72 % Ausbeute zu erhalten. Anschließende Ethersynthese mit Epichlorhydrin, gefolgt von Epoxidöffnung mittels Isopropylamin führte zu Practolol **5** in einer Ausbeute von 56 % über zwei Stufen. Nachfolgend unternahmen wir die Synthese von Diloxanidfuroat **8**, einem luminalem Amöbizid, welches zur Behandlung von Amöbeninfektionen eingesetzt wird (Schema 5 b).^[21]^ Die leicht zugängliche Dichloroacetylhydroxamsäure **6** wurde mittels Selen‐Katalyse zum entsprechenden *para*‐Dichloroacetylaminophenol **7** mit 57 % Ausbeute umgesetzt. Das Einführen einer Furoylgruppe gefolgt von *N*‐Methylierung vervollständigen nachfolgend die Synthese von **8**.

**Scheme 5:**
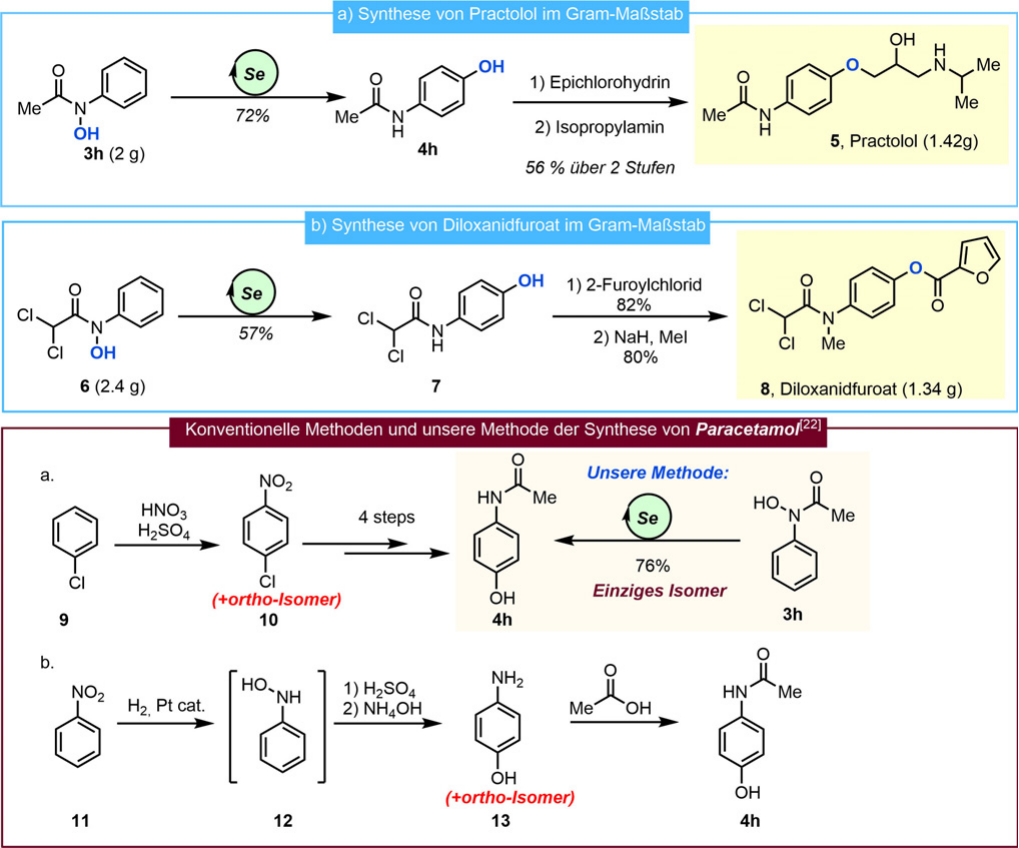
Gram‐Maßstab Synthese von a) Practolol und b) Dioxanidfuroat mittels Selen‐Katalyse. c) Vergleich der Methoden zur Herstellung von Paracetamol und unserer einstufigen, regioselektiven Methode.

Paracetamol/*para*‐Acetoaminophenol **4 h**, eines der weitverbreitetsten und meistproduzierten Medikamente weltweit, wird konventionell über mehrere Stufen synthetisiert. Einige repräsentative Synthesemethoden sind in Schema 5 c gezeigt.^[22]^ Die erste Route verläuft mittels Nitrierung von Chlorobenzol **9**, wobei auch das *ortho*‐Chloronitrobenzol als Nebenprodukt gebildet wird.^[22]^ Prozesse, welche mittels Bamberger Umlagerung verlaufen erzeugen auch signifikante Mengen von *ortho*‐Aminophenol.^[22]^ Im Gegensatz hierzu ermöglicht unsere Selen‐katalysierte *para*‐Hydroxylierung eine außerordentlich regioselektive Alternativlösung, da *para*‐Aminophenol **4 h** ausgehend vom einfachen Startmaterial **3 h** als exklusives Regioisomer in herausragender Ausbeute hergestellt werden kann.

Zusammenfassend haben wir eine katalytische Methode zur Synthese von *para*‐Aminophenolen ausgehend von den entsprechenden Hydroxamsäuren vorgestellt. Diese katalytische Reaktion verläuft mittels eines einzigartigen, elektrophilem Selen‐induzierten N‐O‐Bindungsbruchprozesses welcher durch nachfolgende zweifache [2,3]‐sigmatrope Umlagerung unter Zuhilfenahme eines weiteren Substratmoleküls zum *para*‐Aminophenol führt. Der vorgestellte Mechanismus wird durch ^18^O‐Überkreuzungsexperimente als auch quantenchemische Berechnungen gestützt. Dieser operativ einfache Prozess toleriert eine außerordentliche Breite an funktionellen Gruppen und kann leicht zur Synthese von z. B. Practolol **5** und Diloxanidfuroat **8** im Gram‐Maßstab eingesetzt werden.

## Conflict of interest

Die Autoren erklären, dass keine Interessenkonflikte vorliegen.

## Supporting information

As a service to our authors and readers, this journal provides supporting information supplied by the authors. Such materials are peer reviewed and may be re‐organized for online delivery, but are not copy‐edited or typeset. Technical support issues arising from supporting information (other than missing files) should be addressed to the authors.

Supplementary

Supplementary

Supplementary
