# Supplementary material for: Redox‐Neutrale Selen‐katalysierte Isomerisierung von para‐Hydroxamsäuren zu para‐Aminophenolen
Source: Angew Chem Weinheim Bergstr Ger. 2021 Mar 24;133(25):13896–901. doi: 10.1002/ange.202100801 (PMC10946912; doi:10.1002/ange.202100801)
Supplement: Supplementary file 3 — Supplementary [file ANGE-133-13896-s003.zip › SI_ACIE_Hydroxylation_12Feb2021_woTitle.pdf]

Table of Contents

1. General Information 2
2. Synthesis of Starting Materials 3
   2.1. Preparation of hydroxamic acids: 3
3. Synthesis of *para*-Hydroxylated Amides 10
   3.1. General Procedure: 10
   3.2. ^{18}O-labeling and crossover experiment 27
4. NMR Spectra 29
   4.1. NMR of Starting Materials: 29
   4.2. NMR of Products: 36
5. Computational Details 65
6. References 66

1. General Information

All solvents were distilled from appropriate drying agents prior to use or used as received, if anhydrous. All reagents were used as received from commercial suppliers unless otherwise stated. Reaction progress was monitored by thin layer chromatography (TLC) performed on aluminum plates coated with silica gel F254 with 0.2 mm thickness. Chromatograms were visualized by fluorescence quenching with UV light at 254 nm or by staining using potassium permanganate. Flash column chromatography was performed using silica gel 60 (230-400 mesh, Merck and co.) or prepacked columns (Chromabond silica) using a Biotage Selekt Flash Purification System. Neat infrared spectra were recorded using a Perkin-Elmer Spectrum 100 FT-IR spectrometer. Wavenumbers (ν_{max}) are reported in cm^{-1}. Mass spectra were obtained using a Finnigan MAT 8200 or (70 eV) or an Agilent 5973 (70 eV) spectrometer, using electrospray ionization (ESI). All ^{1}H-NMR and ^{13}C-NMR spectra were recorded using Bruker AV-400, AV-600 or AV-700 spectrometers at 300K. Chemical shifts are given in parts per million (ppm, δ), referenced to the solvent peak of $CDCl_3$, defined at δ = 7.26 ppm (^{1}H-NMR) and δ = 77.16 ppm (^{13}C-NMR), MeOH-d_4, defined at δ = 3.31 ppm (^{1}H-NMR) and δ = 49.00 ppm (^{13}C-NMR), and DMSO-d_6, defined at δ = 2.50 (^{1}H-NMR) and δ = 39.52 ppm (^{13}C-NMR). Coupling constants are quoted in Hz (*J*). ^{1}H , ^{13}C and ^{19}F NMR splitting patterns are designated as singlet (s), doublet (d), triplet (t), quartet (q) as they appeared in the spectrum. If the appearance of a signal differs from the expected splitting pattern, the observed pattern is designated as apparent (app). Splitting patterns that could not be interpreted or easily visualized are designated as multiplet (m) or broad (br).

2. Synthesis of Starting Materials

2.1. Preparation of hydroxamic acids:

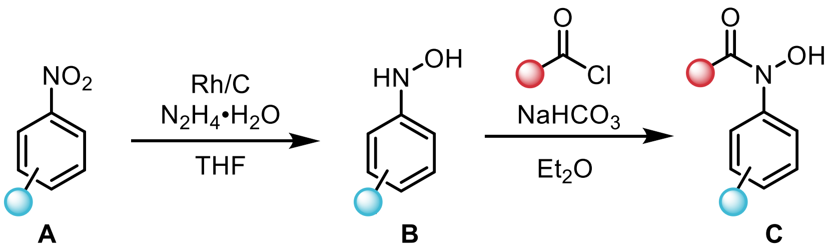

To a solution of nitrobenzene **A** (1.00 equiv.) in THF (0.3 M) at 0°C was added 5-wt% Rh/C (0.30 mol% Rh) and hydrazine monohydrate (1.20 equiv.). The reaction was allowed to warm to room temperature and stirred until all starting material was consumed (as indicated by TLC analysis). The reaction mixture was filtered through a short pad of *Celite*® and the solvent was evaporated under reduced pressure to afford crude hydroxylamine **B**. The crude hydroxylamine was used in the next step without further purification.

To solution of crude hydroxylamine **B** in Et_2O (0.3 M) at 0 °C were added sodium bicarbonate (1.20 equiv.) and acyl chloride (1.20 equiv.). The reaction was allowed to warm to room temperature and stirred until all the starting material was consumed (as indicated by TLC analysis). The reaction mixture was filtered through a filter paper to remove the solids before being concentrated under reduced pressure to afford crude hydroxamic acid **C**. The crude mixture was purified by flash column chromatography (SiO_2, heptane/EtOAc) to give pure hydroxamic acid.

- Characterization of the hydroxamic acids (**3a**, **h**, **j**, **m**[1]; **3d**, **e**[2]; **3i**[3]; **3k**[4]; **3l**[5]; **3p**, **r**[6]; **3q**, **t**[7];**3s**[8]) were in accordance with the reported literature.
- Hydroxamic acid **3b** and **3c** were prepared by EDC coupling.

a) **2-((3r,5r,7r)-adamantan-1-yl)-*N*-hydroxy-*N*-phenylacetamide (3b)**

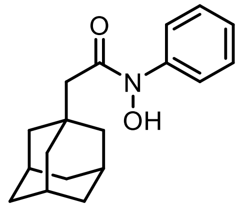

A solution of phenylhydroxylamine (200 mg, 1.87 mmol, 1.00 equiv.) and 1-adamantaneacetic acid (371 mg, 2.06 mmol, 1.10 equiv.) in dichloromethane (0.35 M) at 0 °C was added EDC*HCl (394 mg, 2.06 mmol, 1.10 equiv.). The reaction mixture was allowed to warm to room temperature and stirred for 12 h. Water (5 mL) was added and the layers were separated. The aqueous layer was extracted with dichloromethane (3x5 mL) and the combined organic layers were dried over anhydrous $MgSO_4$. The solvents were removed under reduced pressure and the crude product was purified by flash column chromatography (SiO_2, heptane/EtOAc) to afford 201 mg of hydroxamic acid **3b** (0.71 mmol, 38%). (NMR)

^{1}H NMR (600 MHz, $CDCl_3$): δ 7.26-7.42 (m, 5H), 2.10 (s, 2H) 1.57-1.97 (m, 16H).

^{13}C NMR (150 MHz, $CDCl_3$): δ 177.3, 129.4, 125.6 (2C), 122.5 (2C), 48.9, 42.6, 42.4, 36.8 (3C), 28.71 (3C), 28.69 (3C).

HRMS (ESI$^+$): exact mass calculated for $[M+H]^+$ ($C_{18}H_{24}NO_2$) required *m/z* 286.1802; found *m/z* 286.1802.

FT-IR (neat) ν_{max}: 3100, 2900, 1590, 1250, 1021, 795.

b) ***N*-hydroxy-*N*-phenylcyclobutanecarboxamide (3c)**

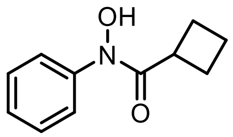

A solution of phenylhydroxylamine (218 mg, 2.00 mmol, 1.00 equiv.) and cyclobutanecarboxylic acid (220 mg, 2.20 mmol, 1.10 equiv.) in dichloromethane (0.35 M) at 0 °C was added EDC*HCl (422 mg, 2.20 mmol, 1.10 equiv.). The reaction mixture was allowed to warm to room temperature and stirred for 12 h. Water (5 mL) was added and the layers were separated. The aqueous layer was extracted with dichloromethane (3x5 mL) and the combined organic layers were dried over anhydrous $MgSO_4$. The solvents were removed under reduced pressure and the crude product was purified by flash column chromatography (SiO_2, heptane/EtOAc) to afford hydroxamic acid **3c** as a yellow oil (145 mg, 38%). (NMR)

^{1}H NMR (600 MHz, DMSO-d$_6$): δ 10.34 (s, 1H), 7.63-7.60 (br. s, 2H), 7.36 (t, *J* = 7.9 Hz, 2H), 7.14 (br. s, 1H), 3.63 (br. s, 1H), 2.26-2.20 (m, 2H), 2.15-2.05 (br. s, 2H), 1.95-1.90 (m, 1H), 1.80-1.77 (m, 1H).

^{13}C NMR (150 MHz, DMSO-d$_6$): δ 173.5 (minor rotamer), 172.9 (major rotamer), 142.0, 128.4 (3C), 124.4, 119.8, 37.6, 24.6 (2C), 17.6.

HRMS (ESI$^+$): exact mass calculated for $[M+H]^+$ ($C_{11}H_{14}NO_2$) required *m/z* 192.1019; found *m/z* 192.1012.

FT-IR (neat) ν_{max}: 3190, 2984, 2945, 2868, 1627, 1592, 1493, 1454, 1444, 1392, 1287, 1245, 1224, 1209, 1177, 1098, 1071, 756, 692, 615.

c) ***N*-hydroxy-*N*-phenyl-4-vinylbenzamide (3g)**

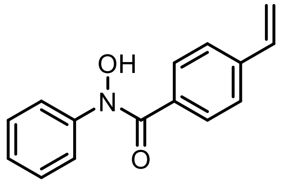

Following the general procedure, phenylhydroxylamine (164 mg, 1.50 mmol, 1.00 equiv.), 4-vinylbenzoyl chloride (300 mg, 1.80 mmol, 1.20 equiv.) and sodium bicarbonate (151 mg, 1.80 mmol, 1.20 equiv.) were stirred at room temperature for 18 h to yield as an off-white solid (137 mg, 38%). (NMR)

^{1}H NMR (400 MHz, DMSO-d$_6$): δ 10.7 (s, 1H), 7.62 (d, *J* = 8.2 Hz, 1H), 7.53 (dd, *J* = 13.3, 8.1 Hz, 2H), 7.39 (t, *J* = 7.9 Hz, 1H), 7.20 (t, *J* = 7.4 Hz, 1H), 6.77 (dd, *J* = 17.7, 11.0 Hz, 1H), 5.93 (d, *J* = 17.7 Hz, 1H), 5.36 (d, *J* = 11.0 Hz, 1H).

^{13}C NMR (100 MHz, DMSO-d$_6$): δ 167.5, 142.1, 138.9, 136.0, 134.7, 129.7, 128.9 (2C), 128.5 (2C), 126.2, 125.5 (2C), 122.2, 116.0.

HRMS (ESI$^+$): exact mass calculated for $[M+Na]^+$ ($C_{15}H_{13}NO_2Na$) required *m/z* 262.0838; found *m/z* 262.0844.

FT-IR (neat) ν_{max}: 3181, 1677, 1617, 1433, 1404, 1385, 991, 752.

d) ***N*-(3-acetylphenyl)-*N*-hydroxyacetamide (3n)**

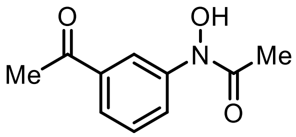

Following the general procedure, 3-acetyl phenylhydroxylamine (203 mg, 2.00 mmol, 1.00 equiv.), acetyl chloride (0.171 ml, 2.40 mmol, 1.20 equiv.) and sodium bicarbonate (202 mg, 2.40 mmol, 1.20 equiv.) were stirred at room temperature for 18 h to yield a yellow oil (226 mg, 58%). (NMR)

^{1}H NMR (600 MHz, DMSO-d$_6$): δ 10.78 (s, 1H), 8.19 (s, 1H), 7.90 (dd, *J* = 8.1, 1.3 Hz, 1H), 7.75 (d, *J* = 7.7 Hz, 1H), 7.52 (t, *J* = 7.9 Hz, 1H), 2.58 (s, 3H), 2.24 (s, 3H).

^{13}C NMR (150 MHz, DMSO-d$_6$): δ 197.6, 170.3, 142.0, 137.0, 128.9 (2C), 124.5, 118.9, 26.8, 22.5.

HRMS (ESI$^+$): exact mass calculated for $[M+H]^+$ ($C_{10}H_{12}NO_3$) required *m/z* 194.0812; found *m/z* 194.0812.

FT-IR (neat) ν_{max}: 3171, 3001, 2917, 1682, 1638, 1582, 1484, 1444, 1372, 1357, 1274, 1212, 1171, 1105, 1082, 1022, 977, 959, 904, 795, 686, 633, 587, 574.

e) ***N*-(3-fluoro-5-iodophenyl)-*N*-hydroxyacetamide (3o)**

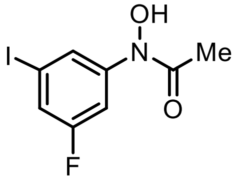

Following the general procedure, 3-fluoro-5-iodo phenylhydroxylamine (506 mg, 2.00 mmol, 1.00 equiv.), acetyl chloride (0.171 ml, 2.40 mmol, 1.20 equiv.) and sodium bicarbonate (202 mg, 2.40 mmol, 1.20 equiv.) were stirred at room temperature for 18 h to yield as an yellow solid (513 mg, 87%). (NMR)

^{1}H NMR (600 MHz, DMSO-d$_6$): δ 10.88 (s, 1H), 7.92 (br. s, 1H), 7.54 (dt, *J* = 11.6, 2.2 Hz, 1H), 7.40 (ddd, *J* = 7.8, 2.3, 1.4 Hz, 1H), 2.23 (s, 3H).

^{13}C NMR (150 MHz, DMSO-d$_6$): δ 170.9, 161.5 (d, J_{CF} = 246.7 Hz), 143.8 (d, J_{CF} = 11.0 Hz), 123.2, 119.4 (d, J_{CF} = 23.7 Hz), 105.6 (d, J_{CF} = 27.3 Hz), 94.1 (d, J_{CF} = 9.4 Hz), 22.8.

^{19}F NMR (565 MHz, DMSO-d$_6$): δ –110.59 (dd, *J* = 11.6, 7.8 Hz).

HRMS (ESI$^+$): exact mass calculated for $[M+H]^+$ ($C_8H_8NO_2FI$) required *m/z* 295.9578; found *m/z* 295.9569.

FT-IR (neat) ν_{max}: 3161, 3107, 3087, 2917, 1627, 1592, 1575, 1450, 1424, 1367, 1336, 1267, 1233, 1152, 1110, 1034, 995, 867, 843, 751, 671, 637, 614, 595, 580, 527, 519, 484, 412.

f) ***N*-hydroxy-*N*-phenylacetamide (3h*)**

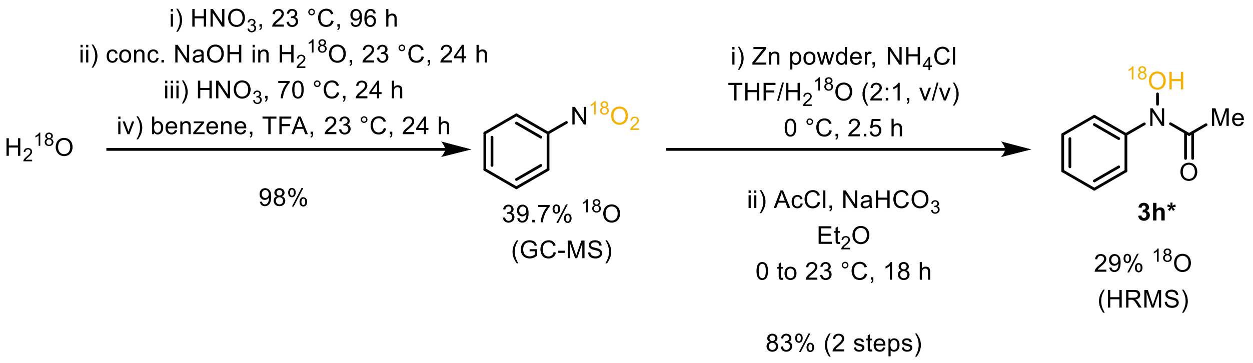

To a 10 mL round-bottom flask was added ^{18}O-water (0.300 mL, 15.0 mmol, 9.74 equiv.) and fuming nitric acid (0.101 mL, 2.40 mmol, 1.56 equiv.). The flask was tightly sealed and stirred at room temperature for 4 d. A solution of NaOH (382 mg, 8.2 mmol, 5.3 equiv.) in ^{18}O-water (0.344 mL, conc. = 24 M) was added and the resulting mixture was stirred additional 24 h at room temperature. Afterwards, the ^{18}O-water was distilled off by heating to 100 °C and fuming HNO_3 (0.016 mL, 0.38 mmol, 0.59 equiv.) was added. The flask was again tightly sealed and the resulting mixture was stirred for 24 h at 70 °C. After this time, the pH of the solution was checked (pH > 7) and the remaining liquids were distilled off by heating to 100 °C to yield the crude $NaN^{18}O_3$ which was used in the next step without further purification.

Crude $NaN^{18}O_3$ was dissolved in trifluoroacetic acid (TFA, 7.65 mL, 103 mmol, 66.9 equiv.) and benzene (0.14 mL, 1.54 mmol, 1.00 equiv.) was added (*Nota bene: the use of fresh TFA/ampule-sealed HPLC-TFA is recommended, as atmospheric water as an impurity in TFA can lead to lower ^{18}O-content of the final product*). The resulting mixture was stirred for 24 h at room temperature before being quenched by the addition of saturated NaOH solution in ^{18}O-water until pH > 10 was reached. The mixture was then extracted with Et_2O (3x10 mL), the combined organic layers were dried over Na_2SO_4 and the solvents were removed under reduced pressure to yield ^{18}O-nitrobenzene (185 mg, 1.50 mmol, 98%; ^{18}-O content = 39.7 % by GC-MS) as a yellow oil which was used without further purification.[9]

To a solution of ^{18}O-nitrobenzene (86.2 mg, 0.700 mmol) in THF/^{18}O-H_2O (2.30 mL, 2:1 (v:v), 0.3 M) was added NH_4Cl (150 mg, 2.8 mmol, 4.00 equiv.). The resulting mixture was cooled to 0 °C before Zn powder (183 mg, 2.8 mmol, 4.00 equiv.) was added. The resulting mixture was stirred

at 0 °C for 2.5 h and subsequently filtered through a pad of *Celite®* and eluded with EtOAc. The solvents were removed under reduced pressure and the crude was used in the next step without further purification.

To a solution of ^{18}O-hydroxylamine (76.4 mg, 0.700 mmol, 1.00 equiv.) in Et_2O (3.50 mL, 0.2 M) at 0 °C was added $NaHCO_3$ (70.6 mg, 0.840 mmol, 1.20 equiv.) and dropwise AcCl (59.7 µL, 0.840 mmol, 1.20 equiv.). The resulting mixture was allowed to warm to room temperature over the course of 18 h. After all starting material had been consumed (as indicated by TLC analysis), the solids were filtered off and the filtrate was washed with dichloromethane. The solvents were removed under reduced pressure and the crude product was purified by column chromatography (SiO_2, heptane/EtOAc) to afford the labeled hydroxamic acid as a dark orange oil (83 mg, 0.55 mmol, 78%) with an ^{18}O-content of 29% (calculated from HRMS [M+H$^+$]) and residual methylanilidine as an inseparable impurity. (NMR)

^{1}H NMR (400 MHz, DMSO-d$_6$): δ 10.61 (s, br, 1H), 7.62 (d, *J* = 7.8 Hz, 2H), 7.36 (t, *J* = 7.9 Hz, 2H), 7.14 (t, *J* = 7.1 Hz, 1H), 2.20 (s, 3H).

^{13}C NMR (100 MHz, DMSO-d$_6$): δ 168.3, 141.7, 128.4 (2C), 124.6, 119.0 (2C), 22.5.

HRMS (ESI$^+$): exact mass calculated for [M+H]$^+$ ($C_8H_{10}NO^{18}O$) required *m/z* 154.0749; found *m/z* 154.0746.

FT-IR (neat) ν_{max}: 2918, 1643, 1595, 1547, 1308, 1100, 1033, 906, 69.

3. Synthesis of *para*-Hydroxylated Amides

3.1. General Procedure:

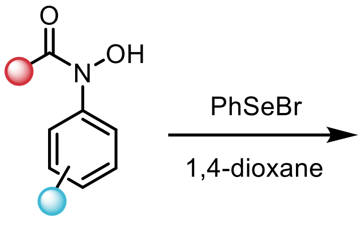

C **D**

To a solution of arylhydroxamic acid **C** (1.00 equiv.) in 1,4-dioxane (0.2 M) was added phenylselenyl bromide (10.0 mol%). The reaction mixture was stirred at room temperature or at 100 °C until all starting material was consumed (as indicated by TLC analysis). The solvents were evaporated under reduced pressure and the crude *para*-aminophenol was purified by flash column chromatography (SiO_2, heptane/EtOAc) to yield the pure *para*-aminophenol **D**.

a) ***N*-(4-hydroxyphenyl)pivalamide (4a):**

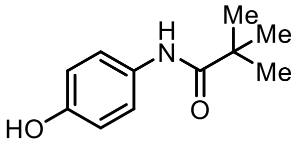

Following the general procedure using *N*-hydoxy-*N*-phenylpivalamide **3a** (60.0 mg, 0.31 mmol, 1.00 equiv.). The reaction was stirred at 100 °C for 8 h. A white solid was obtained (42 mg, 70%). (NMR)

^{1}H NMR (600 MHz, DMSO-d$_6$): δ 9.11 (s, br, 1H), 8.92 (s, br, 1H), 7.36-7.34 (m, 2H), 6.68-6.65 (m, 2H), 1.19 (s, 9H).

^{13}C NMR (150 MHz, DMSO-d$_6$): δ 175.9, 153.3, 130.8, 122.3 (2C), 114.7 (2C), 38.9, 27.4 (3C).

HRMS (ESI$^+$): exact mass calculated for $[M+H]^+$ ($C_{11}H_{16}NO_2$) required *m/z* 194.1176; found *m/z* 194.1176.

FT-IR (neat) ν_{max}: 3299, 1666, 1614, 1594, 1554, 1489, 1371, 1300, 1264, 783, 692.

b) **2-((3r,5r,7r)-adamantan-1-yl)-*N*-(4-hydroxyphenyl)acetamide (4b):**

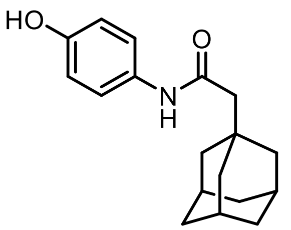

Following the general procedure using adamantyl hydroxamic acid **3b** (60 mg, 0.22 mmol, 1.00 equiv.). The reaction was stirred at 60 °C for 7 h. A white solid was obtained (43 mg, 72%). (NMR)

^{1}H NMR (600 MHz, DMSO-d$_6$): δ 9.46 (s, br, 1H), 9.12 (s, 1H), 7.34 (d, *J* = 12.0 Hz, 2H), 6.66 (d, *J* = 6.0 Hz, 2H), 1.98 (s, 2H), 1.91 (s, br, 3H), 1.67-1.57 (m, 12H).

^{13}C NMR (150 MHz, DMSO-d$_6$): δ 168.4, 153.1, 131.0, 121.0 (2C), 115.0 (2C), 66.4, 50.8, 42.1 (3C), 36.5 (3C), 28.1 (3C).

HRMS (ESI$^+$): exact mass calculated for $[M+H]^+$ ($C_{18}H_{24}NO_2$) required *m/z* 286.1802; found *m/z* 286.1795.

FT-IR (neat) ν_{max}: 3188, 2922, 1605, 1254, 1028, 839.

c) ***N*-(4-hydroxyphenyl)cyclobutanecarboxamide (4c):**

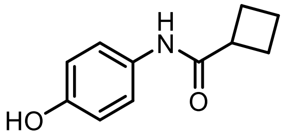

Following the general procedure using *N*-hydroxy-*N*-phenylcyclobutanecarboxamide (1.01 g, 5.28 mmol, 1.00 equiv.). The reaction was stirred at 23 °C for one hour and then heated at 100 °C for 2 h. An off-white solid was obtained (711 mg, 70%). (NMR)

^{1}H NMR (600 MHz, DMSO-d$_6$): δ 9.42 (s, 1H), 9.11 (s, 1H), 7.37-7.35 (m, 2H), 6.68-6.65 (m, 2H), 3.15 (pd, *J* = 8.6, 0.7 Hz, 1H), 2.23-2.16 (m, 2H), 2.09-2.03 (m, 2H), 1.95-1.87 (m, 1H), 1.81-1.75 (m, 1H).

^{13}C NMR (150 MHz, DMSO-d$_6$): δ 172.1, 153.1, 131.0, 120.9 (2C), 115.0 (2C), 39.5, 24.6 (2C), 17.8.

HRMS (ESI$^+$): exact mass calculated for $[M+H]^+$ ($C_{11}H_{14}NO_2$) required *m/z* 192.1019; found *m/z* 192.1016.

FT-IR (neat) ν_{max}: 3300, 2946, 1738, 1643, 1605, 1546, 1511, 1440, 1367, 1219, 833.

d) ***N*-(4-hydroxyphenyl)-4-nitrobenzamide (4d):**

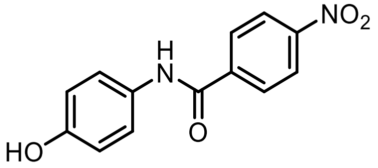

Following the general procedure using *N*-hydroxy-4-nitro-*N*-phenylbenzamide (60 mg, 0.23 mmol, 1.00 equiv.). The reaction was stirred at 23 °C for 4 h. A white solid was obtained (50 mg, 83%). (NMR)

^{1}H NMR (600 MHz, DMSO-d$_6$): δ 10.34 (s, br, 1H), 9.38 (s, br, 1H), 8.33 (d, *J* = 8.7 Hz, 2H), 8.15 (d, *J* = 8.6 Hz, 2H), 7.54 (d, *J* = 8.8 Hz ,2H), 6.76 (d, *J* = 8.9 Hz ,2H).

^{13}C NMR (150 MHz, DMSO-d$_6$): δ 163.4, 154.2, 149.0, 140.9, 130.3, 129.1 (2C), 123.6 (2C), 122.5 (2C), 115.2 (2C).

HRMS (ESI$^+$): exact mass calculated for $[M+H]^+$ ($C_{13}H_{11}N_2O_4$) required *m/z* 259.0713; found *m/z* 259.0709.

FT-IR (neat) ν_{max}: 3247, 1625, 1584, 1521, 1348, 834, 718, 693.

e) ***N*-(4-hydroxyphenyl)-4-nitrobenzamide (2):**

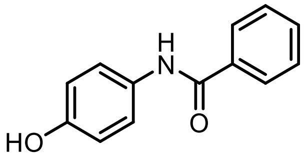

Following the general procedure using *N*-hydroxy-*N*-phenylbenzamide (50 mg, 0.23 mmol, 1.00 equiv.). The reaction was stirred at 23 °C for 3 h. A white solid was obtained (37 mg, 76%). (NMR)

^{1}H NMR (600 MHz, DMSO-d$_6$): δ 10.01 (s, br, 1H), 9.26 (s, br, 1H), 7.92 (d, *J* = 7.8 Hz, 2H), 7.57-7.49 (m, 5 H), 6.73 (d, *J* = 8.7, 2H).

^{13}C NMR (150 MHz, DMSO-d$_6$): δ 165.0, 153.7, 135.2, 131.3, 130,7, 128.4 (2C), 127.5 (2C), 122.3 (2C), 115.0 (2C).

HRMS (ESI$^+$): exact mass calculated for $[M+H]^+$ ($C_{13}H_{12}NO_2$) required *m/z* 214.0863; found *m/z* 214.0867.

FT-IR (neat) ν_{max}: 3169, 2191, 1631, 1507, 1384, 1098, 818.

f) ***N*-(4-hydroxyphenyl)-4-methoxybenzamide (4e):**

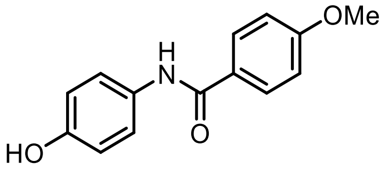

Following the general procedure using *N*-hydroxy-4-methoxy-*N*-phenylbenzamide (60 mg, 0.25 mmol, 1.00 equiv.). The reaction was stirred at 23 °C for 2 h. A white solid was obtained (37 mg, 62%). (NMR)

^{1}H NMR (600 MHz, DMSO-d$_6$): δ 9.85 (s, br, 1H), 9.22 (s, br, 1H), 7.93-7,91 (m, 2H), 7.51-7.48 (m, 2H), 7.05-7.02 (m, 2H), 6.73-6,71 (m, 2H), 3.83 (s, 3H).

^{13}C NMR (150 MHz, DMSO-d$_6$): δ 164.0, 161.7, 153.6, 130.8, 129.4 (2C), 127.2, 122.3 (2C), 115.0 (2C), 113.6 (2C), 55.4.

HRMS (ESI$^+$): exact mass calculated for $[M+H]^+$ ($C_{14}H_{14}NO_3$) required *m/z* 244.0968; found *m/z* 244.0966.

FT-IR (neat) ν_{max}: 3188, 2922, 1605, 1254, 1028, 839.

g) ***N*-(4-hydroxy-2,3-dimethylphenyl)cinnamamide (4f):**

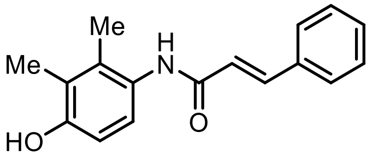

Following the general procedure using *N*-(2,3-dimethylphenyl)-*N*-hydroxycinnamamide (60 mg, 0.22 mmol, 1.00 equiv.). The reaction was stirred at 23 °C for 8 h. A white solid was obtained (44 mg, 73%). (NMR)

^{1}H NMR (600 MHz, DMSO-d$_6$): δ 9.43 (s, 1H), 9.22 (s, 1H), 7.61, (d, *J* = 7.2 Hz, 2H), 7.53 (d, *J* = 15.8 Hz, 1H), 7.4-7.37 (m, 3H), 6.98 (d, *J* = 8.5 Hz, 1H), 6.89 (d, *J* = 15.8 Hz, 1H), 6.66 (d, *J* = 8.5 Hz, 1H), 2.08 (s, 3H), 2.06 (s, 3H).

^{13}C NMR (150 MHz, DMSO-d$_6$): δ 164.0, 153.1, 139.5, 135.1, 133.0, 129.6, 129.1 (2C), 127.7 (2C), 127.6, 124.0, 122.8, 122.5, 112.0, 14.7, 12.3.

HRMS (ESI$^+$): exact mass calculated for $[M+H]^+$ ($C_{17}H_{18}NO_2$) required *m/z* 268.1332; found *m/z* 268.1322.

FT-IR (neat) ν_{max}: 3360, 1660, 1620, 1578, 1528, 1269, 1203, 1048, 1022, 795, 539.

h) ***N*-(4-hydroxyphenyl)-4-vinylbenzamide (4g):**

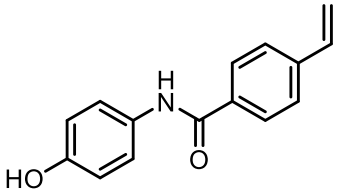

Following the general procedure using *N*-hyrdoxy-*N*-phenyl-4-vinylbenzamide (55 mg, 0.23 mmol, 1.00 equiv.). The reaction was stirred at 23 °C for 8 h. An off-white solid was obtained (31 mg, 56%). (NMR)

^{1}H NMR (400 MHz, DMSO-d$_6$): δ 9.99 (s, 1H), 9.24 (s, 1H), 7.92 (d, *J* = 8.3 Hz, 2H), 7.60 (d, *J* = 8.3 Hz, 2H), 7.52 (d, *J* = 8.8 Hz, 2H), 6.81 (dd, *J* = 17.7, 11.0 Hz, 1H), 6.73 (d, *J* = 8.9 Hz, 2H), 6.98 (d, *J* = 17.8 Hz, 1H), 5.39 (d, *J* = 11.1 Hz, 1H).

^{13}C NMR (100 MHz, DMSO-d$_6$): δ 164.4, 153.7, 139.8, 135.9, 134.2, 130.7, 127.9 (2C), 126.0 (2C), 122.3 (2C), 116.3, 115.0 (2C).

HRMS (ESI$^+$): exact mass calculated for $[M+Na]^+$ ($C_{15}H_{13}NNaO_2$) required *m/z* 262.0839; found *m/z* 262.0844.

FT-IR (neat) ν_{max}: 3316, 2925, 2362, 1692, 1641, 1608, 1539, 1515, 1258, 1125, 918.

i) ***N*-(4-hydroxyphenyl)acetamide (4h):**

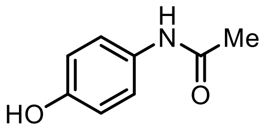

Following the general procedure using *N*-hyrdoxy-*N*-phenylacetamide (62 mg, 0.41 mmol, 1.00 equiv.). The reaction was stirred at 23 °C for 3 h. A white solid was obtained (47 mg, 76%). (NMR)

^{1}H NMR (600 MHz, DMSO-d$_6$): δ 9.66 (s, 1H), 9.20 (s, 1H), 7.33 (d, *J* = 8.6 Hz, 2H), 6.67 (d, *J* = 8.6 Hz, 2H), 1.97 (s, 3H).

^{13}C NMR (150 MHz, DMSO-d$_6$): δ 167.9, 153.3, 131.1, 121.1 (2C), 115.2 (2C), 23.8.

HRMS (ESI$^+$): exact mass calculated for $[M+H]^+$ ($C_8H_{10}NO_2$) required *m/z* 152.0706; found *m/z* 152.0697.

FT-IR (neat) ν_{max}: 3256, 1657, 1553, 1511, 1257, 1236, 1047, 1023, 833.

j) ***N*-(4-hydroxynaphthalen-1-yl)acetamide (4i):**

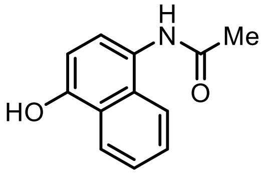

Following the general procedure using *N*-hydroxy-*N*-(naphthalene-1-yl)acetamide (90 mg, 0.44 mmol, 1.00 equiv.). The reaction was stirred at 23 °C for 12 h. An off-white solid was obtained (57 mg, 62%). (NMR)

^{1}H NMR (600 MHz, DMSO-d$_6$): δ 10.08 (s, 1H), 9.62 (s, 1H), 8.14 (d, *J* = 8.0 Hz, 1H), 7.88 (d, *J* = 8.3 Hz, 1H), 7.51 (ddd, *J* = 8.3, 6.8, 1.3 Hz, 1H), 7.46 (ddd, *J* = 7.9, 6.8, 1.0 Hz, 1H), 7.30 (d, *J* = 8.0 Hz, 1H), 6.83 (d, *J* = 8.0 Hz, 1H).

^{13}C NMR (150 MHz, DMSO-d$_6$): δ 168.9, 151.2, 129.8, 126.0, 125.0, 124.7, 124.6, 123.6, 122.8, 122.3, 107.3, 23.1.

HRMS (ESI$^+$): exact mass calculated for $[M+H]^+$ ($C_{12}H_{12}NO_2$) required *m/z* 202.0863; found *m/z* 202.0868.

FT-IR (neat) ν_{max}: 3256, 1657, 1553, 1511, 1257, 1236, 1047, 1023, 833.

k) ***N*-(4-hydroxy-3-methylphenyl)acetamide (4j):**

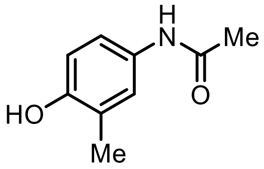

Following the general procedure using *N*-hydroxy-*N*-(*m*-tolyl-1-yl)acetamide (60 mg, 0.36 mmol, 1.00 equiv.). The reaction was stirred at 23 °C for 8 h. A white solid was obtained (42 mg, 70%). (NMR)

^{1}H NMR (600 MHz, MeOH-d$_4$): δ 7.17 (d, *J* = 2.2 Hz, 1H), 7.14 (dd, *J* = 8.5, 2.2 Hz, 1H), 6.67 (d, *J* = 8.5 Hz, 1H), 2.16 (s, 3H), 2.07 (s, 3H).

^{13}C NMR (150 MHz, MeOH-d$_4$): δ 171.3, 153.5, 131.4, 125.8, 124.7, 120.6, 115.4, 23.5, 16.3.

HRMS (ESI$^+$): exact mass calculated for $[M+H]^+$ ($C_9H_{12}NO_2$) required *m/z* 166.0863; found *m/z* 166.0866.

FT-IR (neat) ν_{max}: 3284, 1636, 1617, 1503, 1415, 1263, 1209, 1114, 818.

l) ***N*-(4-hydroxy-2,6-dimethylphenyl)acetamide (4k):**

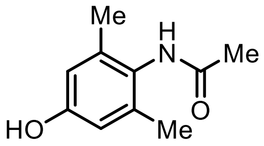

Following the general procedure using *N*-(2,6-dimethylphenyl)-*N*-hydroxyacetamide (60 mg, 0.36 mmol, 1.00 equiv.). The reaction was stirred at 100 °C for 8 h. A white solid was obtained (28 mg, 47%). (NMR)

^{1}H NMR (600 MHz, MeOH-d$_4$): δ 6.51 (s, 2H), 2.13 (s, 3H), 2.12 (s, 6H).

^{13}C NMR (150 MHz, MeOH-d$_4$): δ 172.6, 157.3, 138.0 (2C), 127.4, 115.2 (2C), 22.3, 18.4 (2C).

HRMS (ESI$^+$): exact mass calculated for $[M+H]^+$ ($C_{10}H_{14}NO_2$) required *m/z* 180.1019; found *m/z* 180.1023.

FT-IR (neat) ν_{max}: 3254, 2954, 1704, 1637, 1597, 1523, 1291, 1150, 1028, 830.

m) ***N*-(4-hydroxy-3,5-dimethylphenyl)acetamide (4l):**

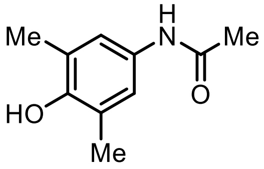

Following the general procedure using *N*-(3,5-dimethylphenyl)-*N*-hydroxyacetamide (75mg, 0.42 mmol, 1.00 equiv.). The reaction was stirred at 23 °C for 8 h. A white solid was obtained (32 mg, 43%). (NMR)

^{1}H NMR (600 MHz, MeOH-d$_4$): δ 7.06 (s, 2H), 2.18 (s, 6H), 2.06 (s, 3H).

^{13}C NMR (150 MHz, MeOH-d$_4$): δ 171.3, 151.1, 131.5, 126.0 (2C), 122.1 (2C), 23.5, 16.8 (2C).

HRMS (ESI$^+$): exact mass calculated for $[M+H]^+$ ($C_{10}H_{14}NO_2$) required *m/z* 180.1019; found *m/z* 180.1019.

FT-IR (neat) ν_{max}:3297, 2954, 2923, 2853, 1639, 1619, 1552, 1508, 1325, 1114, 964, 794, 757.

n) ***N*-(4-hydroxy-3-methoxylphenyl)acetamide (4m):**

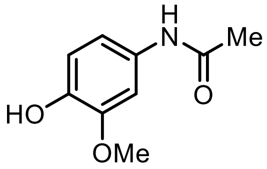

Following the general procedure using *N*-hydroxy-*N*-(3-methoxyphenyl)acetamide (56 mg, 0.31 mmol, 1.00 equiv.). The reaction was stirred at 23 °C for 18 h. A purple solid was obtained (38 mg, 68%). (NMR)

^{1}H NMR (700 MHz, DMSO-d$_6$): δ 9.66 (s, 1H), 8.66 (s, 1H); 7.23 (dd, *J* = 1.8 Hz, 1H), 6.91 (dd, *J* = 8.5, 1.9 Hz, 1H), 6.66 (d, *J* = 8.5 Hz, 1H), 3.71 (s, 3H), 1.97 (s, 3H).

^{13}C NMR (176 MHz, DMSO-d$_6$): δ 167.5, 147.1, 142.3, 131.5, 115.1, 111.6, 104.7, 55.5, 23.8.

HRMS (ESI$^+$): exact mass calculated for $[M+H]^+$ ($C_9H_{12}NO_3$) required *m/z* 182.0812; found *m/z* 182.0812.

FT-IR (neat) ν_{max}: 3304, 3169, 2965, 2841, 1658, 1620, 1549, 1513, 1452, 1418, 967.

o) ***N*-(3-acetyl-4-hydroxyphenyl)acetamide (4n):**

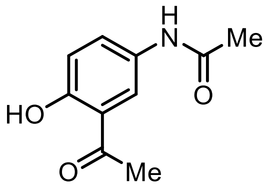

Following the general procedure using *N*-(3-acetylphenyl)-*N*-hydroxyacetamide (48 mg, 0.25 mmol, 1.00 equiv.). The reaction was stirred at 100 °C for 10 h. A white solid was obtained (22 mg, 45%). (NMR)

^{1}H NMR (600 MHz, DMSO-d$_6$): δ 10.1 (s, 1H), 8.14 (t, *J* = 1.8 Hz, 1H), 7.84 (ddd, *J* = 8.1, 2.0, 0.8 Hz, 1H), 7.64 (ddd, *J* = 7.7, 1.5, 1.1 Hz, 1H), 7.44 (t, *J* = 7.9 Hz, 1H), 2.55 (s, 3H), 2.06 (s, 3H).

^{13}C NMR (150 MHz, DMSO-d$_6$): δ 197.7, 168.6, 139.7, 137.3, 129.1, 123.5, 123.1, 118.1, 26.7, 24.0.

HRMS (ESI$^+$): exact mass calculated for $[M+H]^+$ ($C_{10}H_{12}NO_3$) required *m/z* 194.0812; found *m/z* 194.0815.

FT-IR (neat) ν_{max}: 3245, 2922, 1681, 1372, 1275, 797, 687.

p) ***N*-(3-fluoro-4-hydroxy-5-iodophenyl)acetamide (4o):**

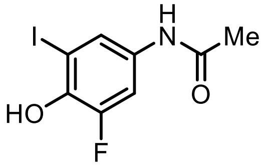

Following the general procedure using *N*-(2-fluoro-6-iodophenyl)-*N*-hydroxyacetamide (60 mg, 0.20 mmol, 1.00 equiv.). The reaction was stirred at 100 °C for 18 h. A white solid was obtained (31 mg, 52%). (NMR)

^{1}H NMR (600 MHz, MeOH-d$_4$): δ 7.63-7.61 (m, 1H), 7.46 (dd, *J* = 12.5, 4.4 Hz, 1H), 2.08 (s, 3H).

^{13}C NMR (150 MHz, MeOH-d$_4$): δ 171.4, 151.0 (d, J_{CF} = 241.2 Hz), 142.9 (d, J_{CF} = 16.0 Hz), 133.4 (d, J_{CF} = 10.0 Hz), 126.6 (d, J_{CF} = 3.3 Hz), 109.7 (d, J_{CF} = 24.2 Hz), 85.7, 23.6.

^{19}F NMR (565 MHz, MeOH-d$_4$): δ –133.41 - –133.43 (m).

HRMS (ESI⁺): exact mass calculated for $[M+H]^+$ ($C_8H_8NO_2FI$) required *m/z* 295.9578; found *m/z* 295.9575.

FT-IR (neat) ν_{max}: 3245, 2922, 1681, 1372, 1275, 797, 687.

q) ***N*-(3-fluoro-4-hydroxyphenyl)acetamide (4p):**

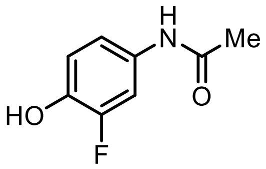

Following the general procedure using *N*-(3-fluorophenyl)-*N*-hydroxyacetamide (60 mg, 0.36 mmol, 1.00 equiv.). The reaction was stirred at 100 °C for 12 h. A white solid was obtained (34 mg, 57%). (NMR)

^{1}H NMR (700 MHz, DMSO-d$_6$): δ 9.82 (s, br, 1H), 9.50 (s, br, 1H), 7.50 (dd, *J* = 13.6, 2.4 Hz, 1H), 7.04 (dd, *J* = 8.7, 1.3 Hz, 1H), 6.85 (t, *J* = 9.3 Hz, 1H), 1.99 (s, 3H).

^{13}C NMR (176 MHz, DMSO-d$_6$): δ 167.9, 150.2 (d, J_{CF} = 238.6 Hz), 140.3 (d, J_{CF} = 12.3 Hz), 131.5 (d, J_{CF} = 9.2 Hz), 117.5 (d, J_{CF} = 3.8 Hz), 115.2 (d, J_{CF} = 3.1 Hz), 107.6 (d, J_{CF} = 22.8 Hz), 23.8.

^{19}F NMR (659 MHz, DMSO-d$_6$): δ –135.00.

HRMS (ESI⁺): exact mass calculated for $[M+H]^+$ ($C_8H_9NO_2F$) required *m/z* 170.0612; found *m/z* 170.0611.

FT-IR (neat) ν_{max}: 3314, 1642, 1517, 1287, 755, 694.

r) ***N*-(2-fluoro-4-hydroxyphenyl)acetamide (4q):**

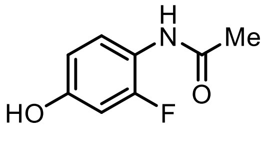

Following the general procedure using *N*-(2-fluorophenyl)-*N*-hydroxyacetamide (60 mg, 0.36 mmol, 1.00 equiv.). The reaction was stirred at 100 °C for 12 h. A white solid was obtained (33 mg, 55%). (NMR)

^{1}H NMR (600 MHz, MeOH-d$_4$): δ 7.39 (td, *J* = 8.9, 0.6 Hz, 1H), 6.58-6.55 (m, 2H), 2.11 (s, 3H).

^{13}C NMR (150 MHz, MeOH-d$_4$): δ 172.2, 157.7 (d, J_{CF} = 11.0 Hz), 157.4 (d, J_{CF} = 245.5 Hz), 127.9 (d, J_{CF} = 3.0 Hz), 118.1 (d, J_{CF} = 12.7 Hz), 111.9 (d, J_{CF} = 2.9 Hz), 103.8 (d, J_{CF} = 22.6 Hz), 22.9.

^{19}F NMR (470 MHz, MeOH-d$_4$): δ –112.67.

HRMS (ESI$^+$): exact mass calculated for $[M+H]^+$ ($C_8H_9NO_2F$) required *m/z* 170.0612; found *m/z* 170.0612.

FT-IR (neat) ν_{max}: 3163, 1634, 1591, 1437, 1378, 1262, 1108, 862, 846, 775, 680.

s) ***N*-(4-hydroxy-3-(trifluoromethyl)phenyl)acetamide (4r):**

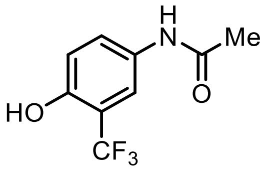

Following the general procedure using *N*-hydroxy-*N*-(3-(trifluoromethyl)phenyl)acetamide (74 mg, 0.34 mmol, 1.00 equiv.). The reaction was stirred at 100 °C for 12 h. A white solid was obtained (37 mg, 50%). (NMR)

^{1}H NMR (600 MHz, MeOH-d$_4$): δ 7.39 (td, *J* = 8.9, 0.6 Hz, 1H), 6.58-6.55 (m, 2H), 2.11 (s, 3H).

^{13}C NMR (150 MHz, MeOH-d$_4$): δ 171.5, 153.6 (d, J_{CF} = 1.6 Hz), 131.5, 126.6, 125.2 (d, J_{CF} = 271.4 Hz), 120.0 (q, J_{CF} = 5.4 Hz), 117.9, 117.7 (q, J_{CF} = 30.8 Hz), 23.5.

^{19}F NMR (565 MHz, MeOH-d$_4$): δ –63.95.

HRMS (ESI$^+$): exact mass calculated for $[M+H]^+$ ($C_9H_9NO_2F_3$) required *m/*z 220.0580; found *m/z* 220.0585.

FT-IR (neat) ν_{max}: 3125, 1630, 1580, 1410, 1251, 1100, 810, 732, 580.

t) ***N*-(3-bromo-4-hydroxy-2-methylphenyl)acetamide (4s):**

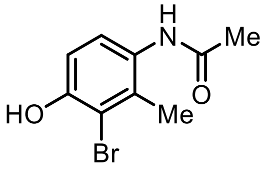

Following the general procedure using *N*-(3-bromo-2-methylphenyl)-*N*-hydroxyacetamide (225 mg, 0.92 mmol, 1.00 equiv.). The reaction was stirred at 23 °C for 3 h and the heated to 100 °C for one hour. A white solid was obtained (109 mg, 48%). (NMR)

^{1}H NMR (600 MHz, MeOH-d$_4$): δ 7.02 (d, *J* = 8.6 Hz, 1H), 6.76 (d, *J* = 8.6 Hz, 1H), 2.28 (s, 3H), 2.12 (s, 3H).

^{13}C NMR (150 MHz, MeOH-d$_4$): δ 172.7, 154.3, 136.8, 127.8 (2C), 114.1 (2C), 22.7, 18.9.

HRMS (ESI$^+$): exact mass calculated for $[M+H]^+$ ($C_9H_{11}NO_2{}^{79}Br$) required *m/z* 243.9968; found *m/z* 243.9969.

FT-IR (neat) ν_{max}: 3297, 3251, 1738, 1642, 1524, 1433, 1371, 1290, 1217, 1026, 811.

u) ***N*-(4-hydroxy-2-iodophenyl)acetamide (4t):**

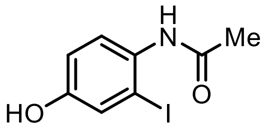

Following the general procedure using *N*-hydroxy-*N*-(2-iodophenyl)acetamide (199 mg, 0.72 mmol, 1.00 equiv.). The reaction was stirred at 23 °C for 12 h. A white solid was obtained (140 mg, 70%). (NMR)

^{1}H NMR (600 MHz, MeOH-d$_4$): δ 7.30 (d, *J* = 2.7 Hz, 1H), 7.13 (d, *J* = 8.6 Hz, 1H), 6.79 (dd, *J* = 8.6, 2.7 Hz, 1H), 2.12 (s, 3H).

^{13}C NMR (150 MHz, MeOH-d$_4$): δ 172.6, 157.9, 132.1, 129.5, 126.4, 116.8, 97.9, 22.9.

HRMS (ESI$^+$): exact mass calculated for $[M+H]^+$ ($C_8H_9NO_2I$) required *m/z* 277.9672; found *m/z* 277.9674.

FT-IR (neat) ν_{max}: 3244, 1702, 1655, 1519, 1425, 1346, 1250, 1029, 806, 503.

v) **Synthesis of Practolol (5):**

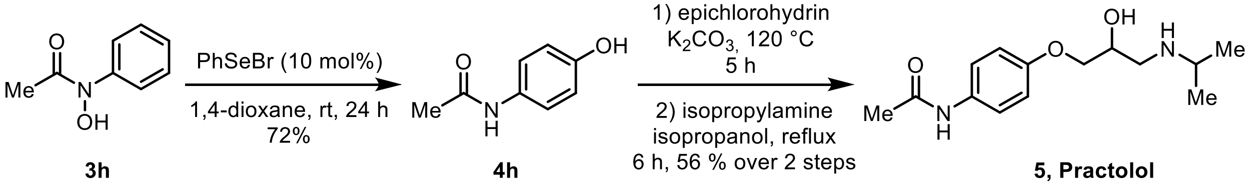

To a solution of hydroxamic acid **3h** (2.00 g, 13.2 mmol, 1.00 equiv.) in 1,4-dioxane (66.0 mL, 0.2 M) was added phenylselenyl bromide (312 mg, 1.32 mmol, 0.10 equiv.) at room temperature. The resulting mixture was then stirred at room temperature for 24 h. After this time, the solvent was removed under reduced pressure and the crude product was purified by column chromatography (SiO_2, heptane/EtOAc) to give acetylated *para*-aminophenol **4h** (1.44 g, 9.50 mmol, 72%) as a white solid.

4h (1.44 g, 9.50 mmol, 1.00 equiv.) was dissolved in epichlorhydrin (2.18 mL, 27.8 mmol, 3.00 equiv.). To the solution was then added K_2CO_3 (306 mg, 2.20 mmol, 0.23 equiv.) and the resulting mixture was heated to 120 °C for 5 h. After this time, the solution was allowed to cool to room temperature and was then concentrated under reduced pressure and the crude epoxide was used in the next step without further purification.

To a solution of the crude epoxide (1.97 g, 9.50 mmol, 1.00 equiv.) in isopropanol (30 mL, 0.3 M) was added isopropylamine (7.60 mL, 92.8 mmol, 9.77 equiv.) at room temperature. The resulting mixture was heated to 100 °C for 6 h. After cooling to room temperature, the solution was concentrated under reduced pressure and the crude product was purified by column chromatography (SiO_2, heptane/EtOAc) to afford practolol **5** (1.42 g, 5.18 mmol, 56% over 2 steps) as a white solid. (NMR)

^{1}H NMR (600 MHz, MeOH-d$_4$): δ 7.40 (d, *J* = 8.9 Hz, 2H), 6.90 (d, *J* = 9.0 Hz, 2H), 4.05-4.01 (m, 1H), 3.96-3.91 (m, 2H), 2.86-2.81 (m, 2H), 2.67-2.64 (dd, *J* = 11.9, 8.6 Hz, 1H), 2.09 (s, 3H), 1.11-1.09 (m, 6H).

^{13}C NMR (150 MHz, MeOH-d$_4$): δ 171.3, 157.0, 133.2, 123.0 (2C), 115.7 (2C), 72.2, 69.8, 50.9, 49.9, 23.6, 22.7, 22.5.

HRMS (ESI$^+$): exact mass calculated for $[M+H]^+$ ($C_{14}H_{23}N_2O_3$) required *m/z* 267.1703; found *m/z* 267.1707.

FT-IR (neat) ν_{max}: 3250, 2952, 1700, 1632, 1540, 1280, 1120, 1021, 829.

w) **Synthesis of Diloxanide Furoate (8):**

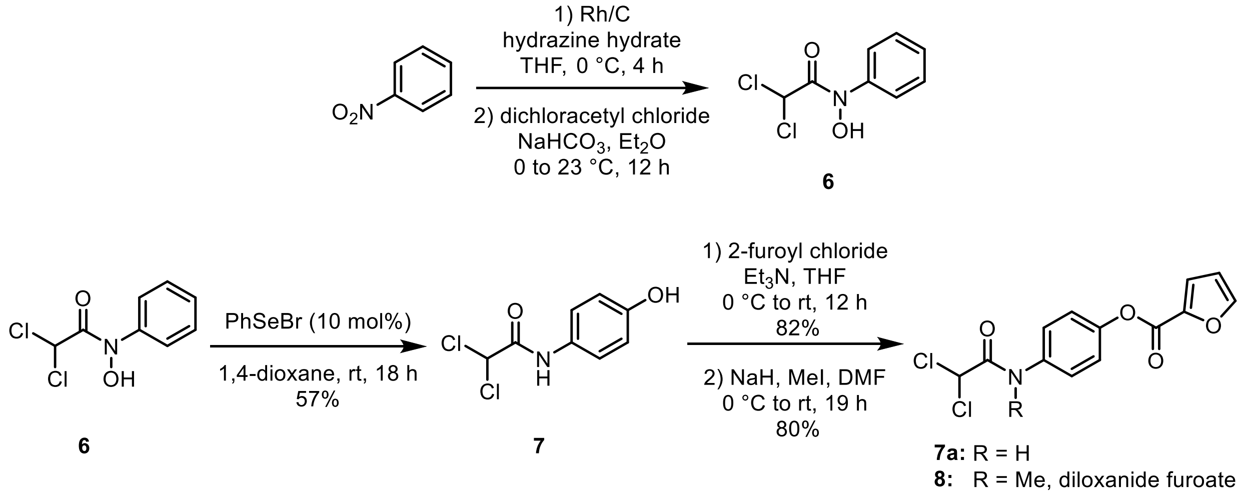

To a solution of nitrobenzene (2.00 g, 16.2 mmol, 1.00 equiv.) in tetrahydrofuran (80 mL, 0.2 M) at 0 °C was added Rh/C (5-wt%) (100 mg, 0.3 mol%) followed by hydrazine hydrate (0.96 mL, 19.5 mmol, 1.20 equiv.). The resulting mixture was stirred at 0 °C for 4 h before being filtered through a pad of *Celite®*. The filtrate was concentrated under reduced pressure to afford crude phenylhydroxylamine, which was dissolved in Et_2O (80 mL, 0.2 M) and cooled to 0 °C. $NaHCO_3$ (1.64 g, 19.5 mmol, 1.20 equiv.) and dichloroacetyl chloride (1.90 mL, 19.5 mmol, 1.20 equiv.) were then added sequentially and the resulting mixture was allowed to warm to room temperature, after which it was stirred for an additional 12 h. After all starting material had been consumed (as indicated by TLC analysis), the mixture was filtered through a paper filter. The solvents were removed under reduced pressure to give crude hydroxamic acid **6** (2.40 g, 10.9 mmol, 66% over two steps). Hydroxamic acid **6** was not stable and was therefore used in the next step without further purification.

To a solution of hydroxamic acid **6** (2.40 g, 10.9 mmol, 1.00 equiv.) in 1,4-dioxane (56 mL, 0.2 M) at room temperature was added phenylselenyl bromide (264 mg, 1.09 mmol, 0.10 equiv.). The resulting mixture was stirred at room temperature for 18 h. After this time, the solvent was removed under reduced pressure and the crude product was purified by column chromatography

(SiO_2, heptane/EtOAc) to afford acylated *para*-aminophenol **7** (1.40 g, 6.21 mmol, 57%) as a white solid. (NMR)

To a solution of **7** (1.40 g, 6.21 mmol, 1.00 equiv.) in tetrahydrofuran (36.5 mL, 0.17 M) at 0 °C was added triethylamine (1.08 mL, 7.82 mmol, 1.26 equiv.) and 2-furoyl chloride (0.77 mL, 7.82 mmol, 1.26 equiv.). The resulting mixture was allowed to warm to room temperature and stirred for an additional 12 h. After this time, water (20 mL) and EtOAc (20 mL) were added and the layers were separated. The aqueous layer was extracted with EtOAc (3x20 mL), the combined organic layers were dried over $MgSO_4$, the solids were filtered off and the solvents were removed under reduced pressure to afford the crude furoate. The crude product was purified by column chromatography (SiO_2, heptane/EtOAc) to afford pure furoate **7a** (1.60 g, 5.09 mmol, 82%). (NMR)

The furoate **7a** (1.60 g, 5.09 mmol, 1.00 equiv.) was dissolved in dimethylformamide (24.2 mL, 0.21 M). The resulting solution was cooled to 0 °C before NaH (60-wt% in paraffin oil, 244 mg, 6.11 mmol, 1.20 equiv.) was added. The reaction mixture was stirred for 30 min at 0 °C before iodomethane (0.38 mL, 6.11 mmol, 1.20 equiv.) was added dropwise. The reaction was allowed to warm to room temperature and stirred for 18 h before the careful addition of water (20 mL) and EtOAc (20 mL). The layers were separated and the aqueous layer was extracted with EtOAc (3x20 mL). The combined organic layers were dried over $MgSO_4$, the solids were filtered off and the solvents were removed under reduced pressure to afford crude diloxanide furoate **8**. The crude product was purified by column chromatography (SiO_2, heptane/EtOAc) to afford pure diloxanide furoate **8** (1.34 g, 4.07 mmol, 80%) as a white solid. (NMR)

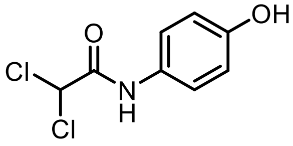

7

^{1}H NMR (600 MHz, MeOH-d$_4$): δ 7.39-7.37 (m, 2H), 6.79-6.76 (m, 2H), 6.34 (s, 1H).

^{13}C NMR (150 MHz, MeOH-d$_4$): δ 164.1, 156.2, 130.3, 123.4 (2C), 116.4 (2C), 68.2.

HRMS (ESI$^+$): exact mass calculated for $[M+H]^+$ ($C_8H_8NO_2{}^{35}Cl_2$) required *m/z* 219.9927; found *m/z* 219.9925.

FT-IR (neat) ν_{max}: 3290, 1669, 1608, 1513, 1439, 1363, 1222, 1167, 832, 812, 664.

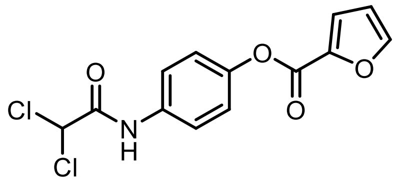

7a

^{1}H NMR (600 MHz, MeOH-d$_4$): δ 7.86 (dd, *J* = 1.7, 0.8 Hz, 1H), 7.69-7.67 (m, 2H), 7.46 (dd, *J* = 3.5, 0.8 Hz, 1H), 7.25-7.22 (m, 2H), 6.70 (dd, *J* = 3.5, 1.8 Hz, 1H), 6.39 (s, 1H).

^{13}C NMR (150 MHz, MeOH-d$_4$): δ 164.4, 158.4, 149.3, 148.5, 145.1, 136.7, 123.3 (2C), 122.5 (2C), 120.9, 113.4, 68.2.

HRMS (ESI$^+$): exact mass calculated for $[M+H]^+$ ($C_{13}H_{10}NO_4{}^{35}Cl_2$) required *m/z* 313.9981; found *m/z* 313.9979.

FT-IR (neat) ν_{max}: 1697, 1611, 1567, 1537, 1509, 1470, 1393, 1295, 1198, 1173, 1089, 1015, 929, 885, 806, 764.

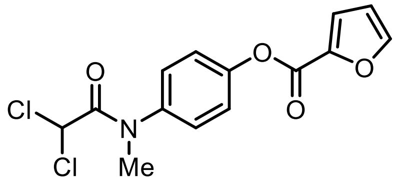

8

^{1}H NMR (600 MHz, CDCl$_3$): δ 8.00 (d, *J* = 3.2 Hz, 1H), 7.71 (d, *J* = 4.7 Hz, 1H), 7.37-7.33 (m, 4H), 7.21-7.20 (m, 1H), 5.91 (s, 1H), 3.35 (s, 3H).

^{13}C NMR (150 MHz, CDCl$_3$): δ 164.1, 160.2, 150.8, 139.2, 135.3, 134.3, 132.2, 128.43 (2C), 128.35, 123.8 (2C), 63.6, 38.8.

HRMS (ESI$^+$): exact mass calculated for $[M+H]^+$ ($C_{14}H_{12}NO_4{}^{35}Cl_2$) required *m/z* 328.0138; found *m/z* 328.0137.

FT-IR (neat) ν_{max}: 1739, 1682, 1503, 1470, 1391, 1234, 1232, 1199, 1170, 1086, 929, 884, 805, 769, 753, 664.

3.2. ^{18}O-labeling and crossover experiment

a) Crossover between 1 and 3h*:

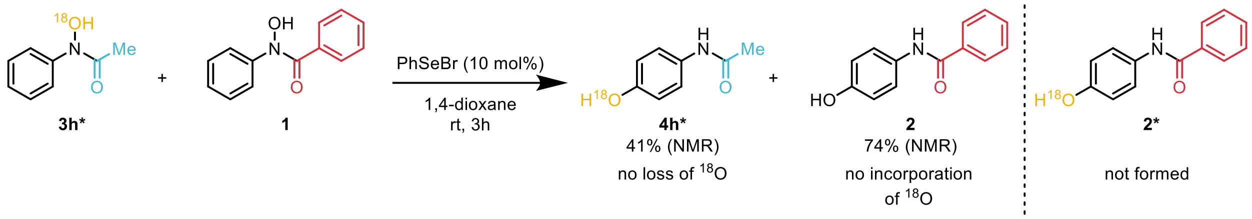

The starting materials **3h*** (27.0 mg, 0.18 mmol, 1.00 equiv.) and **1** (38.4 mg, 0.18 mmol, 1.00 equiv.) were dissolved in 1,4-dioxane (2.00 mL). To the solution was added phenylselenyl bromide (8.49 mg, 0.036 mmol, 20 mol%; 10 mol% for each substrate) and the resulting mixture was stirred for 3 h at room temperature. Afterwards, an internal standard (1,3,5-trimethoxybenzene) was added and the NMR yields were determined (41% **4h***, 74% **2**). The crude mixture was analyzed by HRMS to check for ^{18}O-incorporation.

HRMS (ESI$^+$): exact mass calculated for $[M+H]^+$

4h*: ($C_8H_{10}NO^{18}O$) required *m/z* 154.0749; found *m/z* 154.0748.

2: ($C_{13}H_{12}NO_2$) required *m/z* 214.0863; found *m/z* 214.0867.

2* (hypothetical): ($C_{13}H_{12}NO^{18}O$) required *m/z* 216.0905; not found within error range.

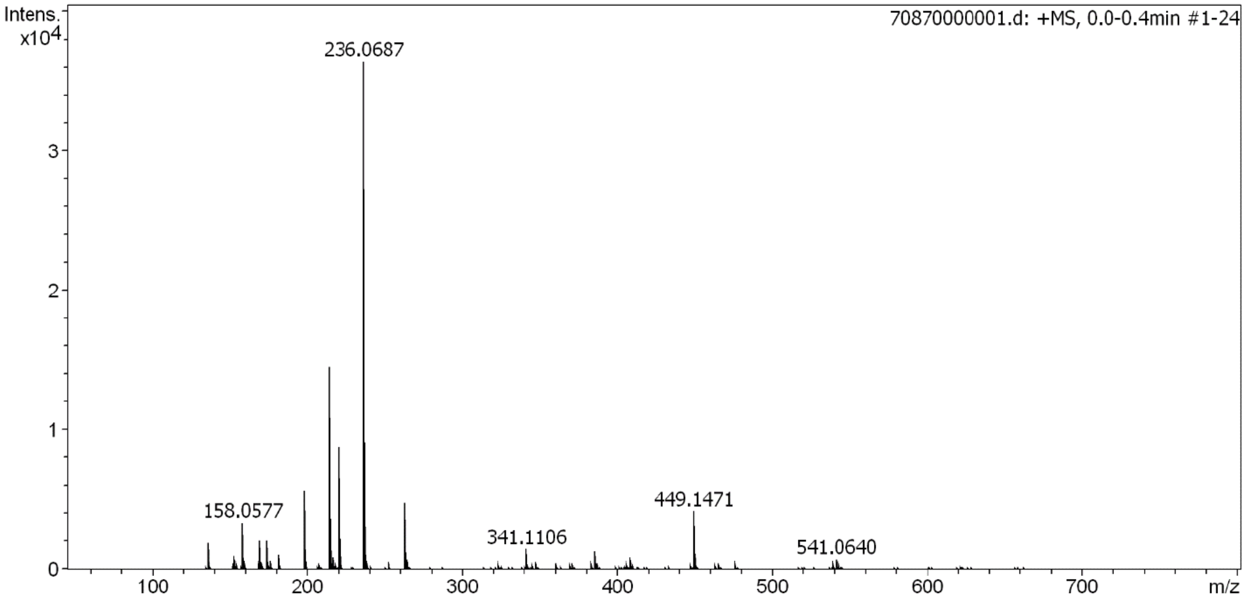

8	152.0708	975
9	153.0741	93
10	154.0748	396
11	157.0497	202
12	158.0577	3353
13	159.0608	334
14	168.0780	365
15	169.0861	2049
16	170.0893	200
17	171.0628	299
18	173.1323	39
19	174.0527	2052
20	175.0559	162
21	176.0569	654

35	214.0867	14519
36	215.0896	1840
37	216.0633	871
38	216.0915	175
39	217.0665	100
40	218.0672	309
41	219.0640	139

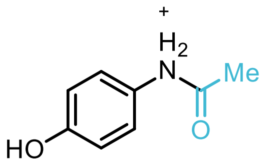

4h*

Chemical Formula: $C_8H_{10}NO_2^+$
Exact Mass: 152,0706

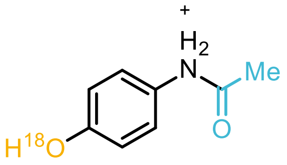

4h*

Chemical Formula: $C_8H_{10}NO^{18}O^+$
Exact Mass: 154,0749

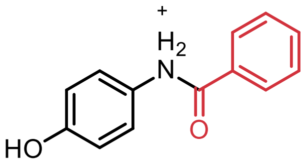

2

Chemical Formula: $C_{13}H_{12}NO_2^+$
Exact Mass: 214,0863

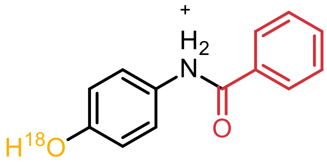

2

Chemical Formula: $C_{13}H_{12}NO^{18}O^+$
Exact Mass: 216,0905

The low intensity of the signal at 216.0915 does not allow for differentiation between the naturally occurring $C_{11}{}^{\mathbf{13}}\mathbf{C_2}H_{12}NO_2$ ($[M+H]^+$ requires *m/z* 216.0930) and the crossover product $C_{13}H_{12}NO^{\mathbf{18}}\mathbf{O}$ ($[M+H]^+$ requires *m/z* 216.0905). However, measuring the commercially available starting material **1,** as received from the supplier (TCI), reveals comparable relative intensities to the material subjected to the crossover. We therefore conclude that no crossover has taken place.

4. NMR Spectra

4.1. NMR of Starting Materials:

a) 2-((3r,5r,7r)-adamantan-1-yl)-*N*-hydroxy-*N*-phenylacetamide (3b):

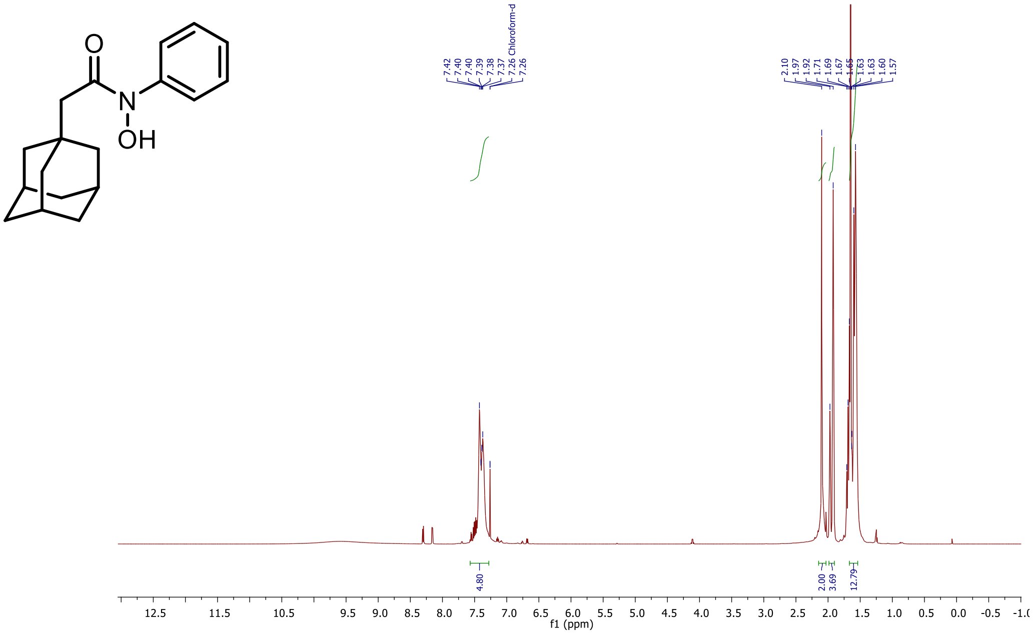

^1H
$CDCl_3$
600 MHz

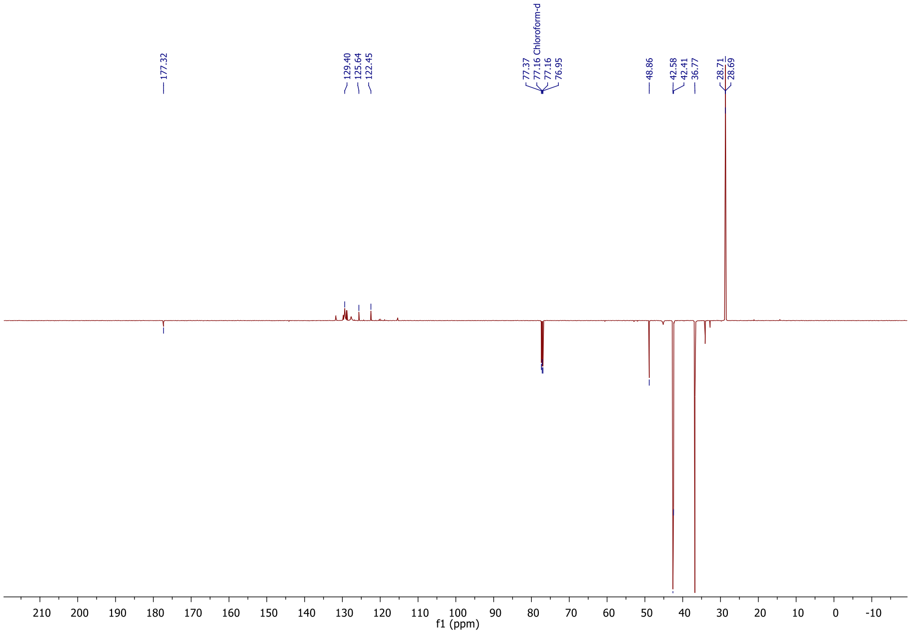

^{13}C
$CDCl_3$
150 MHz

b) ***N*-hydroxy-*N*-phenylcyclobutanecarboxamide (3c)**:

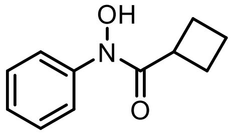

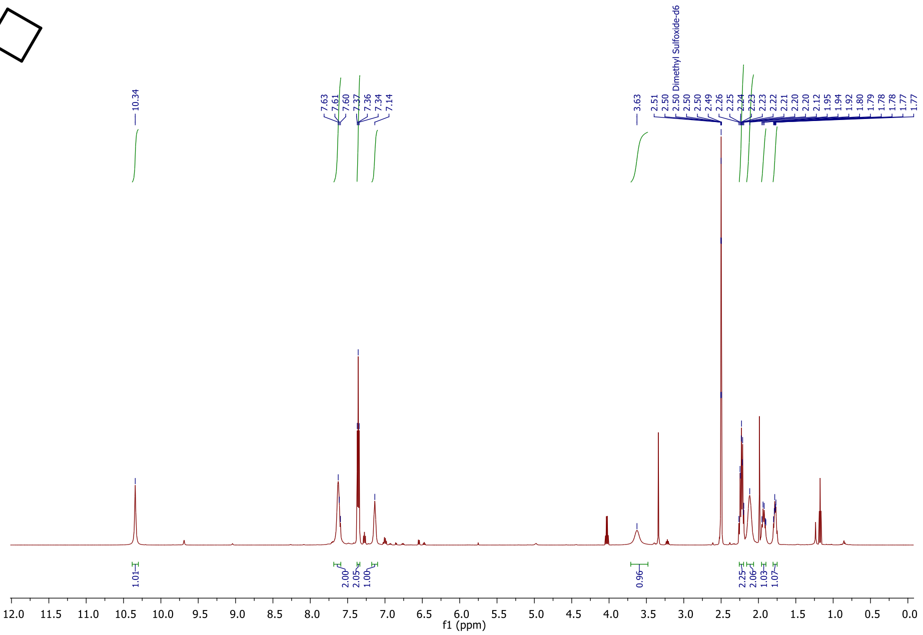

^{1}H
DMSO-d_6
600 MHz

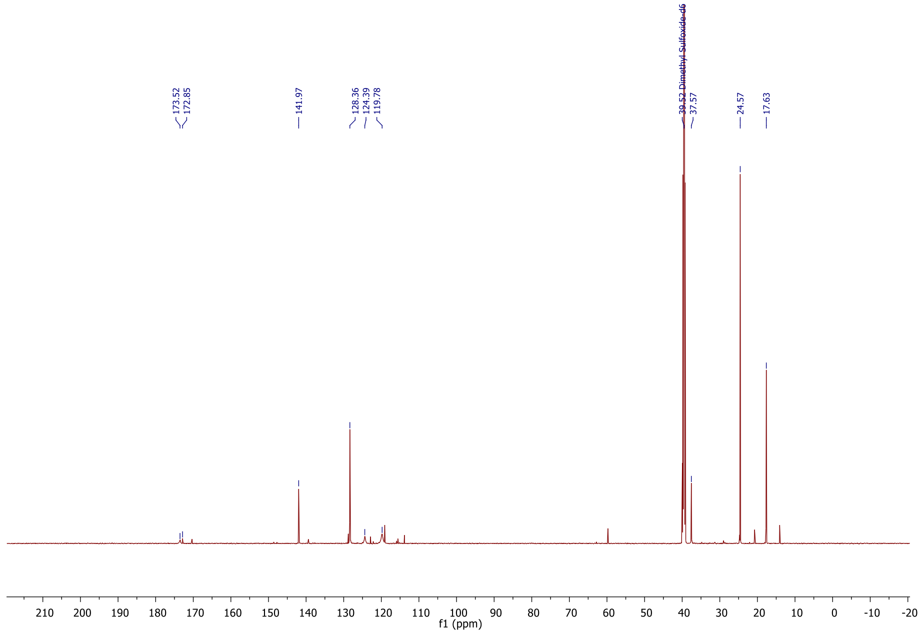

^{13}C
DMSO-d_6
150 MHz

c) ***N*-hydroxy-*N*-phenyl-4-vinylbenzamide (3g)**:

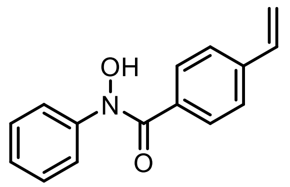

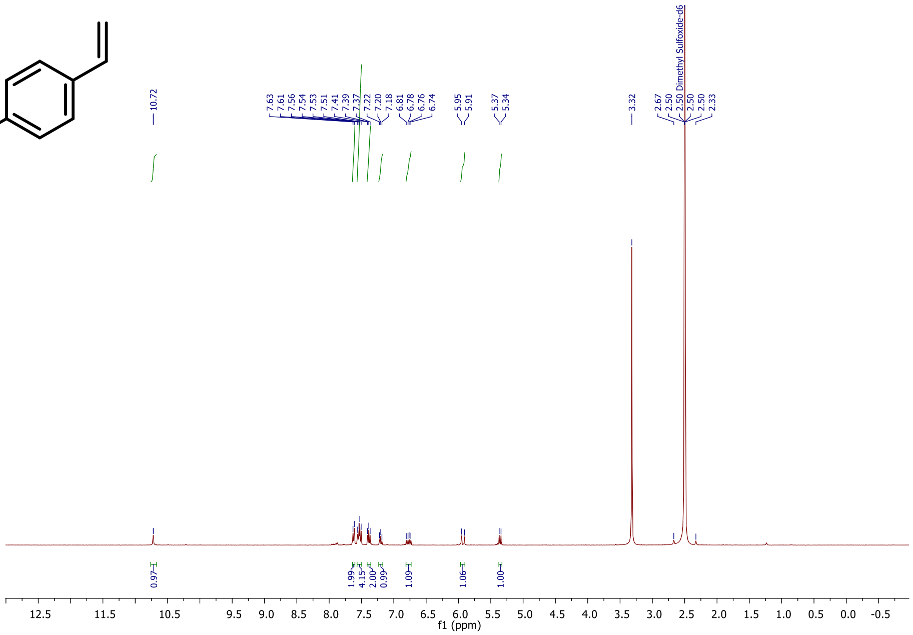

^{1}H
DMSO-d_6
400 MHz

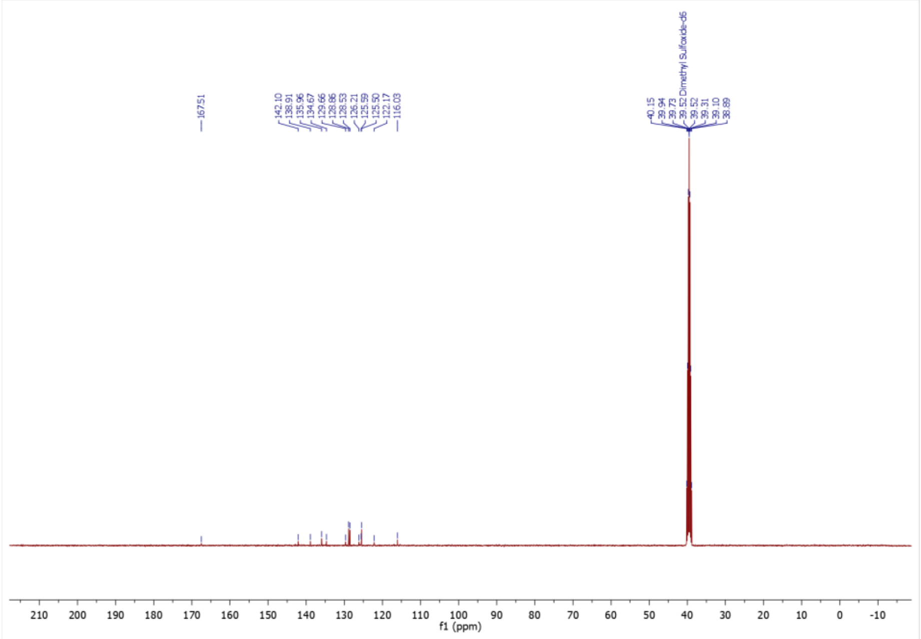

^{13}C
DMSO-d_6
100 MHz

d) ***N*-(3-acetylphenyl)-*N*-hydroxyacetamide (3n)**:

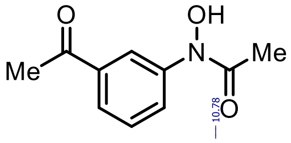

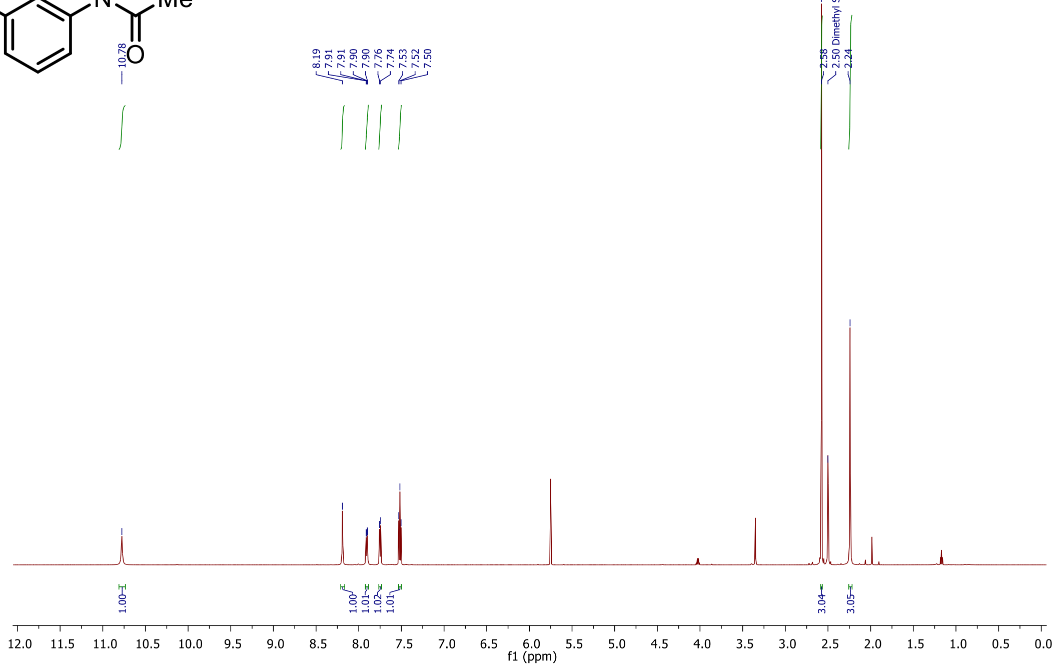

^{1}H
DMSO-d_6
600 MHz

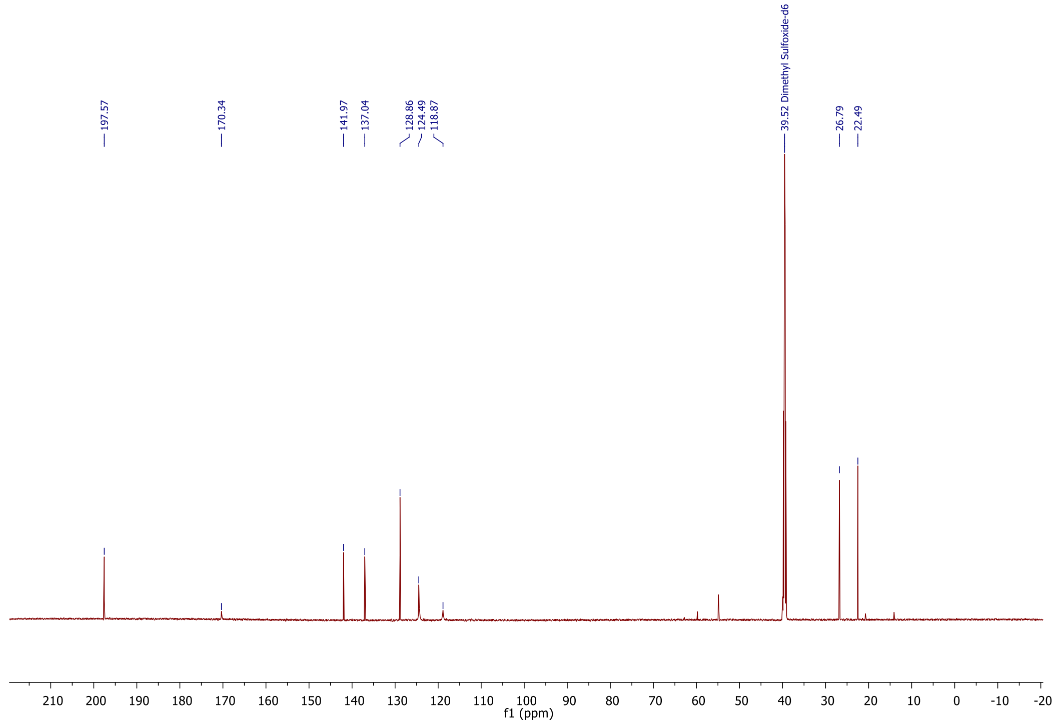

^{13}C
DMSO-d_6
150 MHz

e) ***N*-(3-fluoro-5-iodophenyl)-*N*-hydroxyacetamide (3o)**:

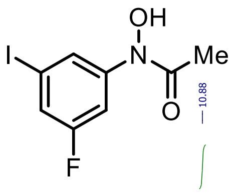

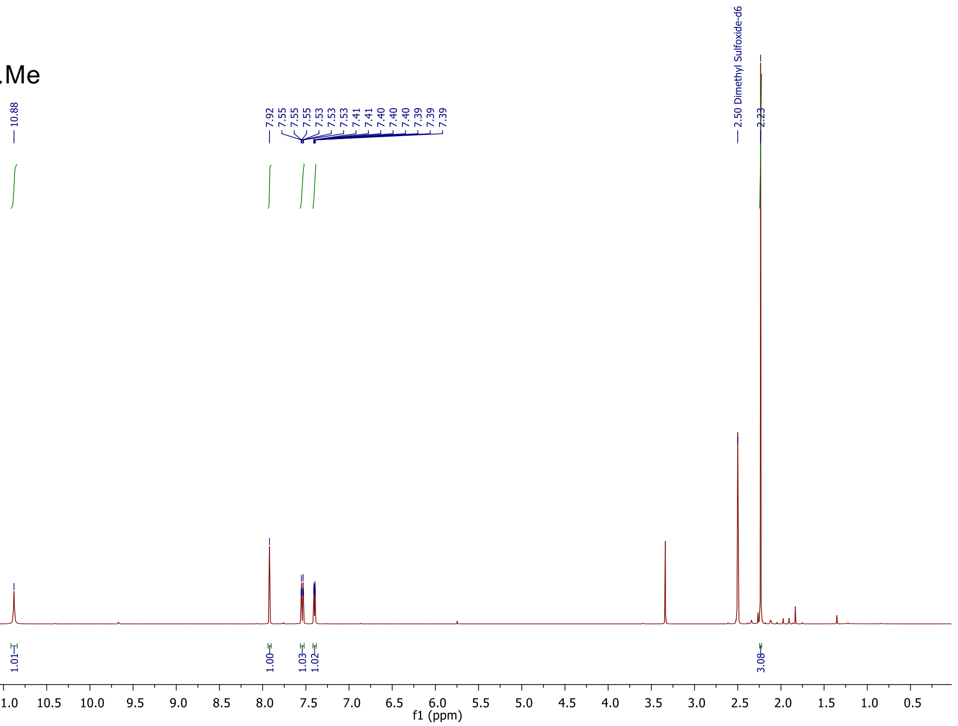

^{1}H
DMSO-d_6
600 MHz

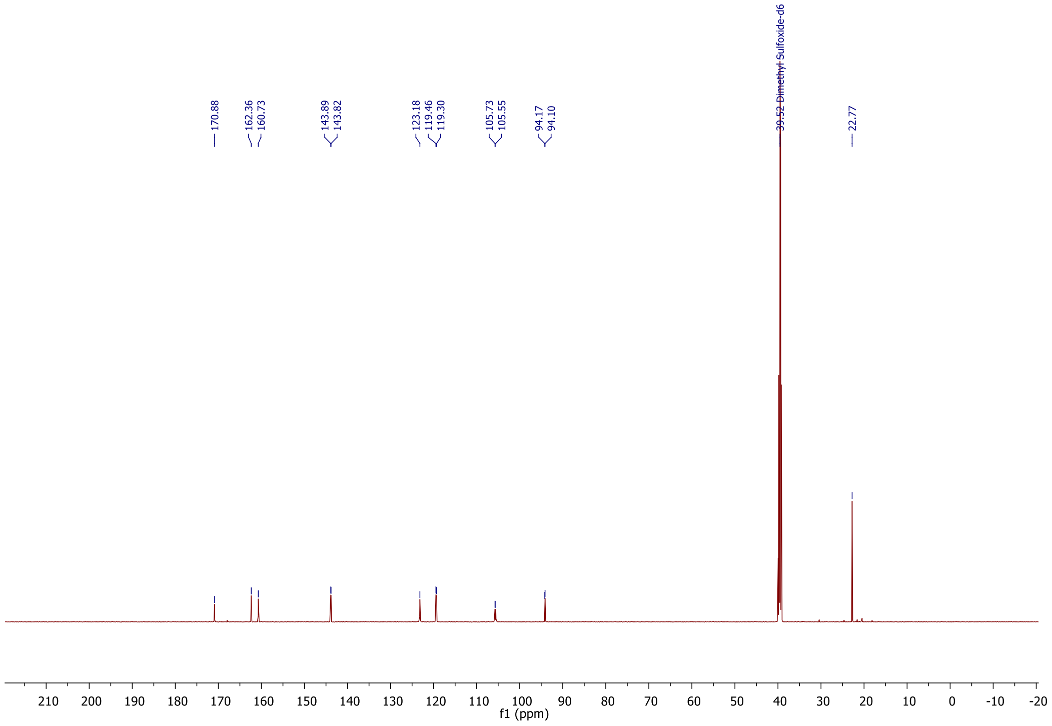

^{13}C
DMSO-d_6
150 MHz

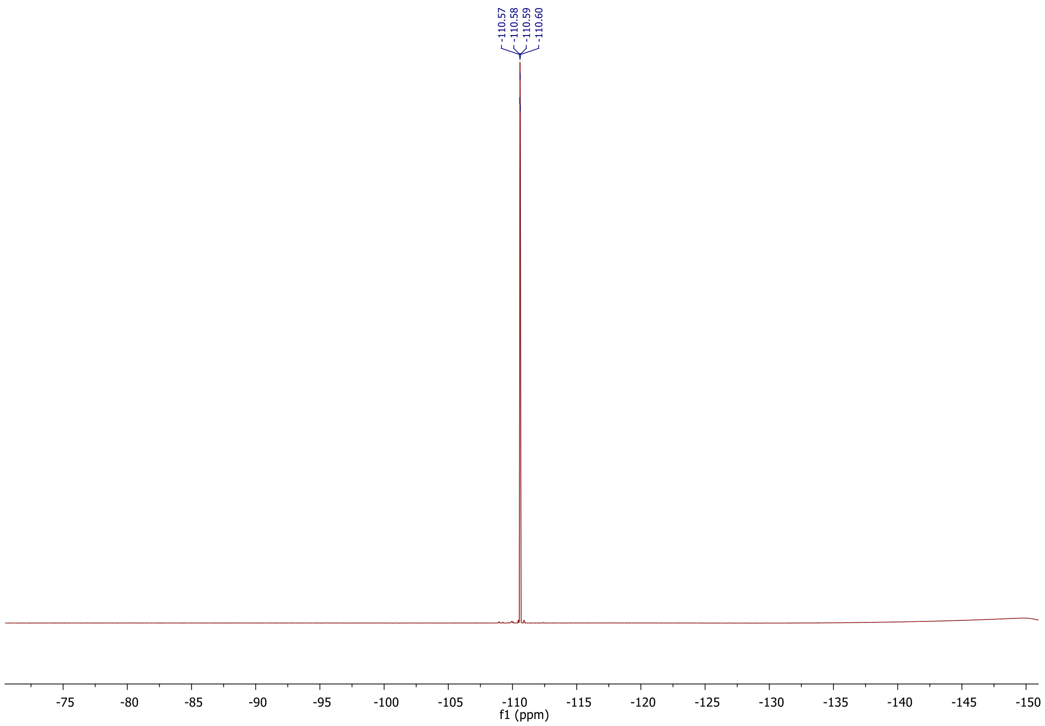

^{19}F
DMSO-d_6
565 MHz

f) ***N*-hydroxy-*N*-phenylacetamide-^{18}O (3h*)**:

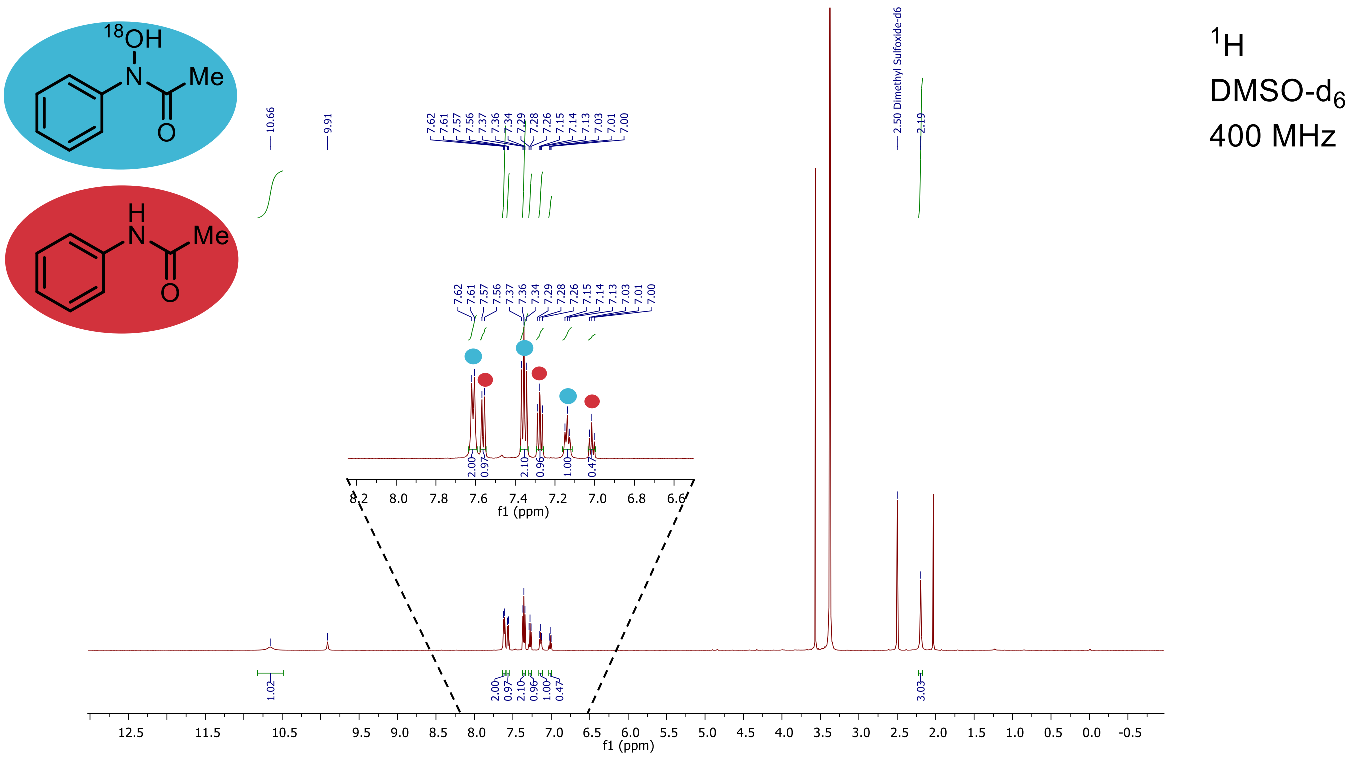

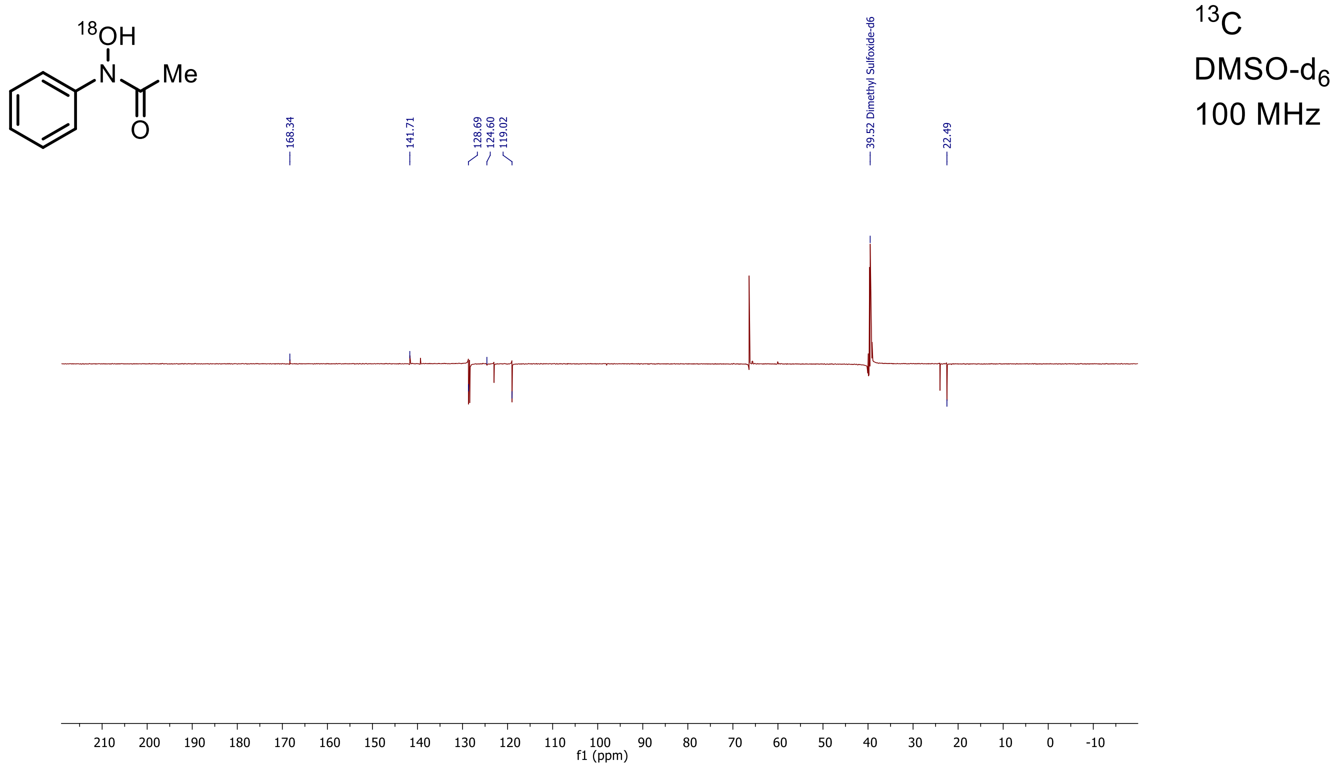

4.2. NMR of Products:

a) ***N*-(4-hydroxyphenyl)pivalamide (4a):**

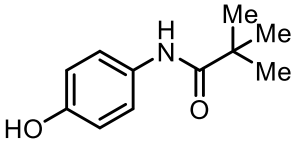

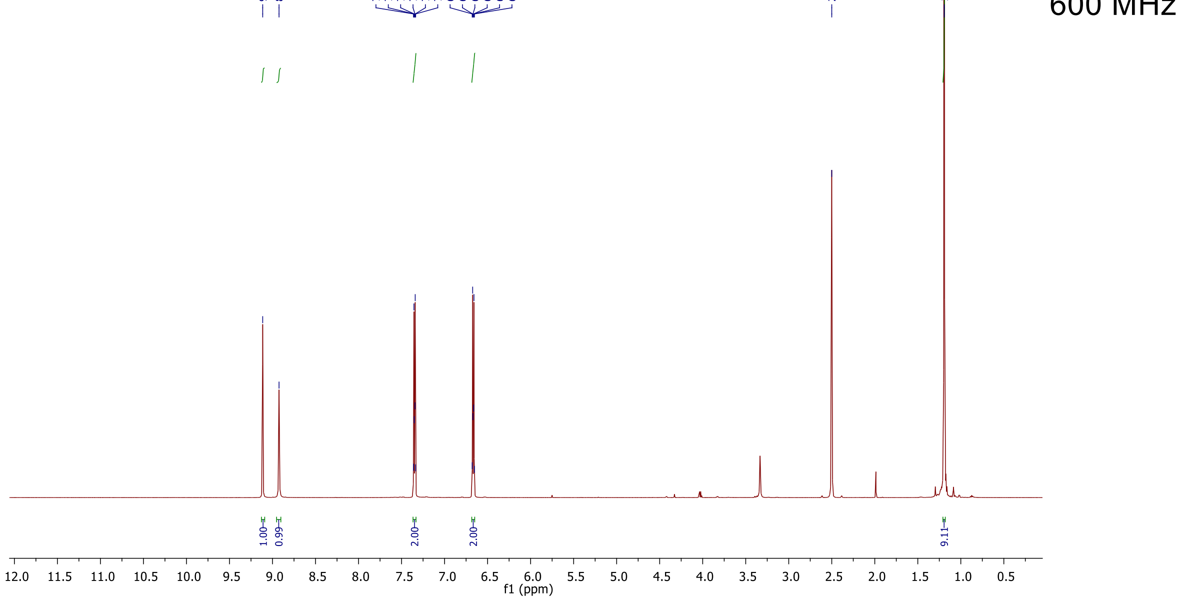

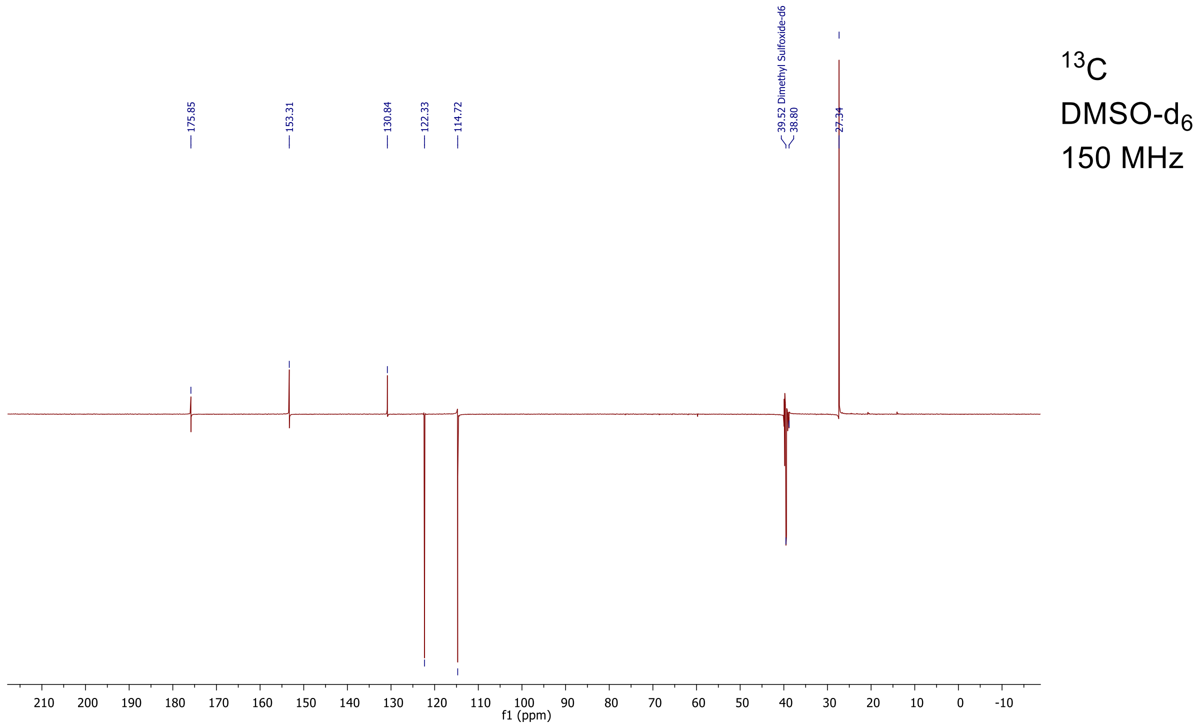

b) **2-((3r,5r,7r)-adamantan-1-yl)-*N*-(4-hydroxyphenyl)acetamide (4b)**:

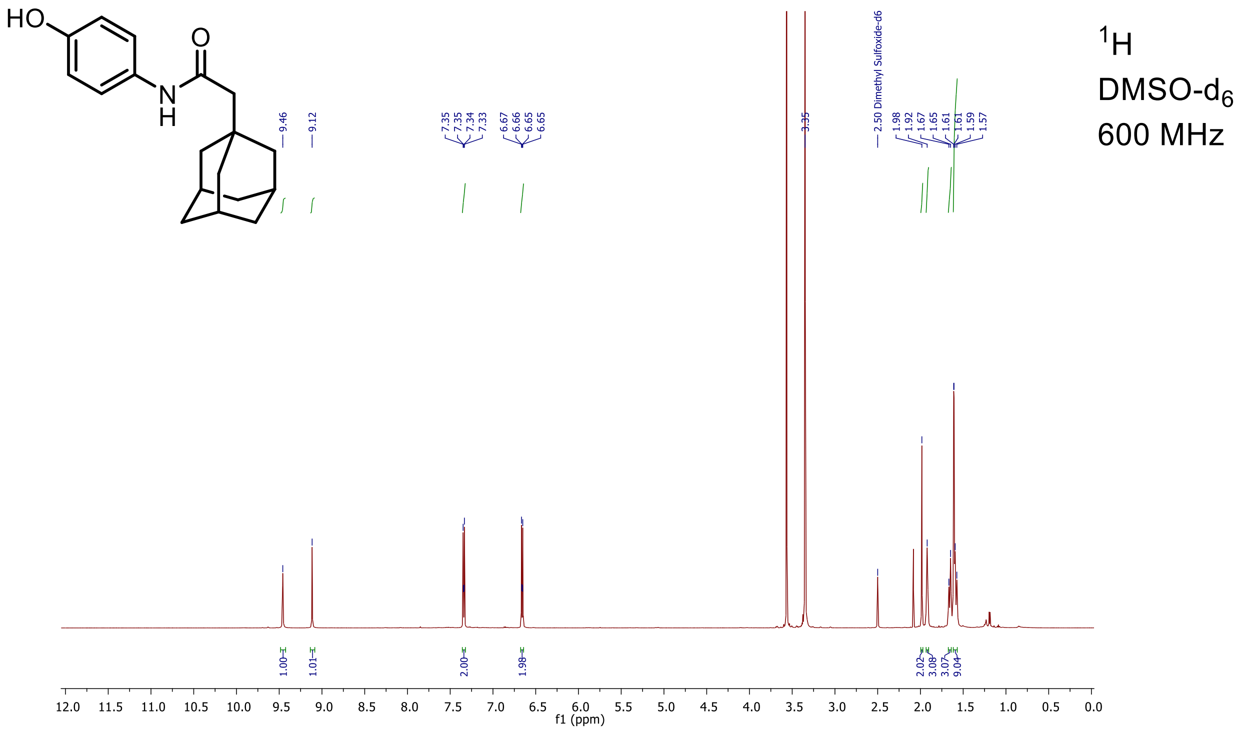

^{1}H
DMSO-d_6
600 MHz

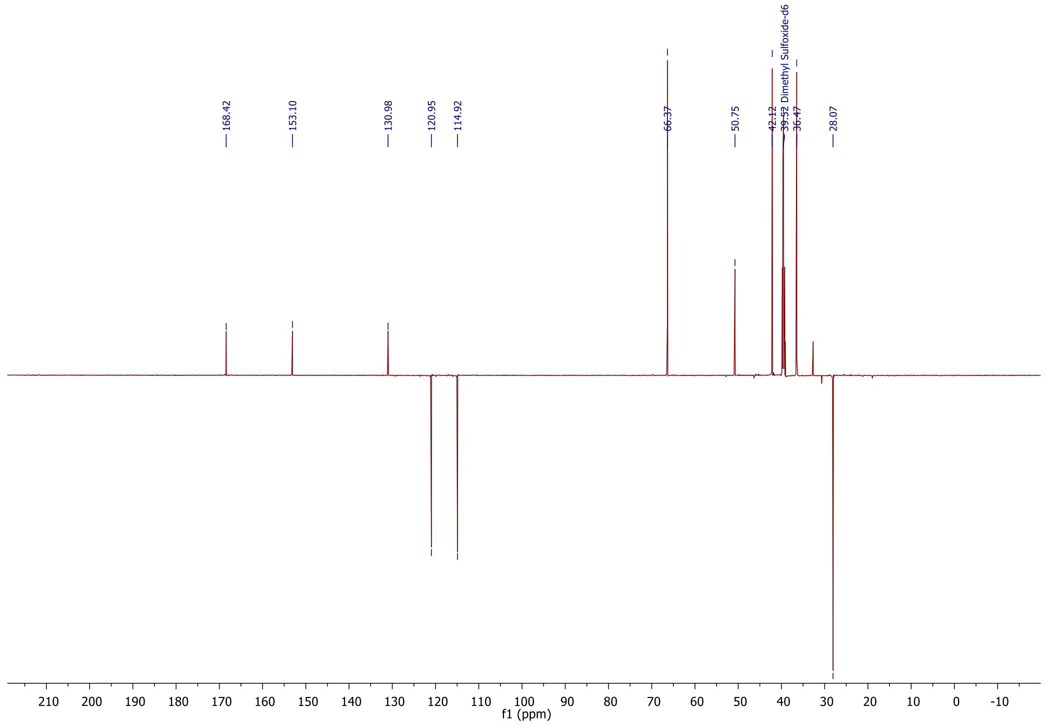

^{13}C
DMSO-d_6
150 MHz

c) *N*-(4-hydroxyphenyl)cyclobutanecarboxamide (4c):

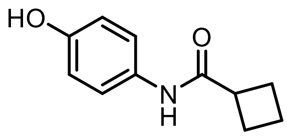

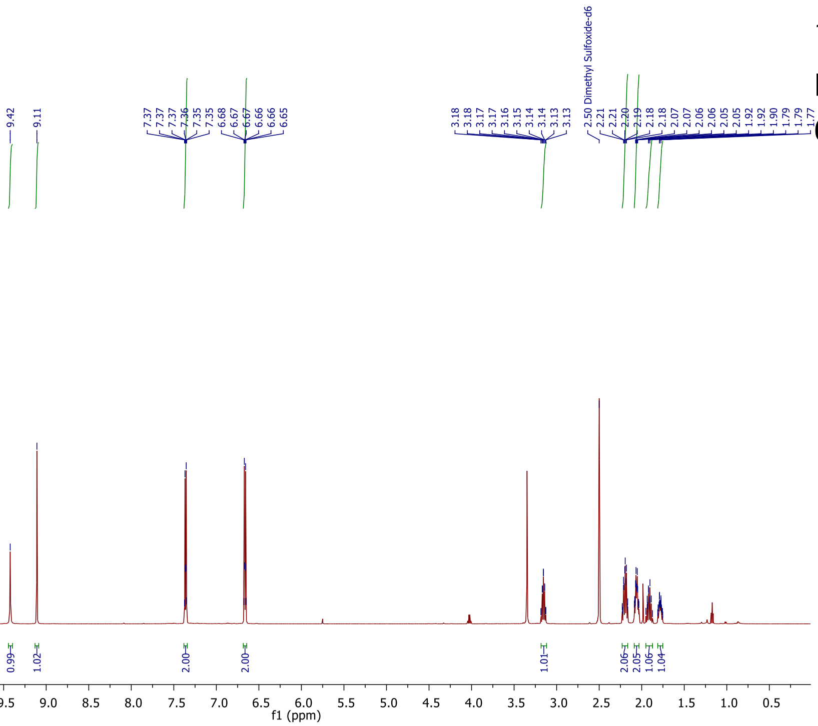

^{1}H
DMSO-d_6
600 MHz

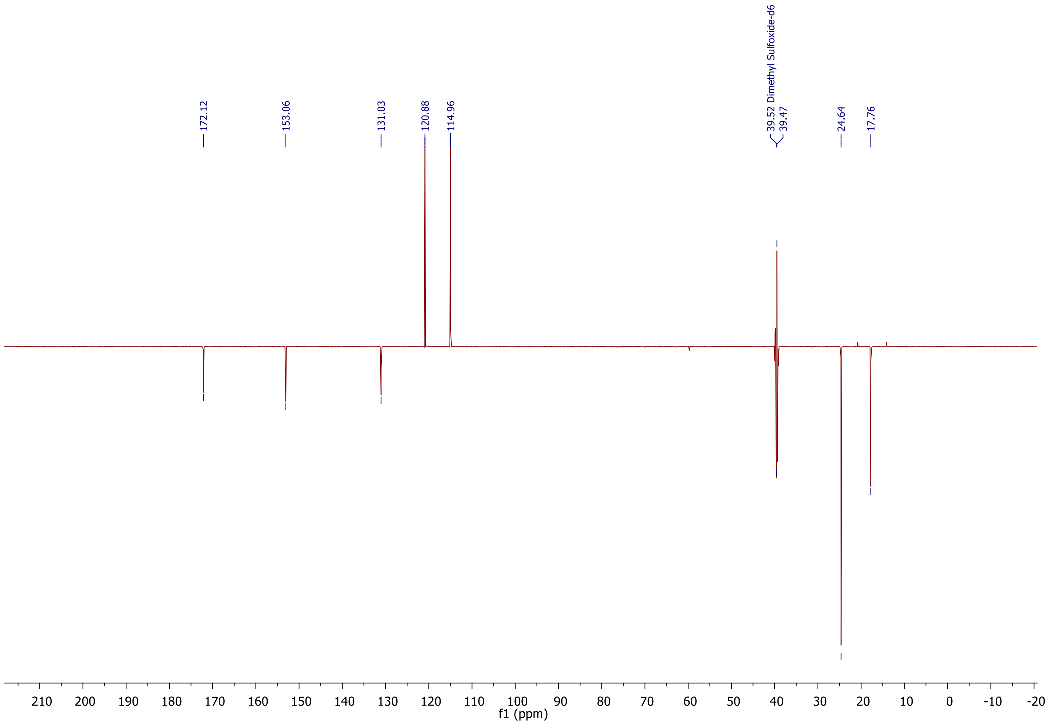

^{13}C
DMSO-d_6
150 MHz

d) ***N*-(4-hydroxyphenyl)-4-nitrobenzamide (4d)**:

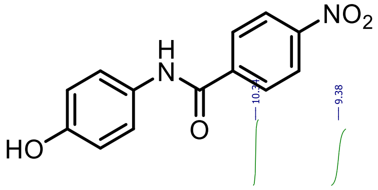

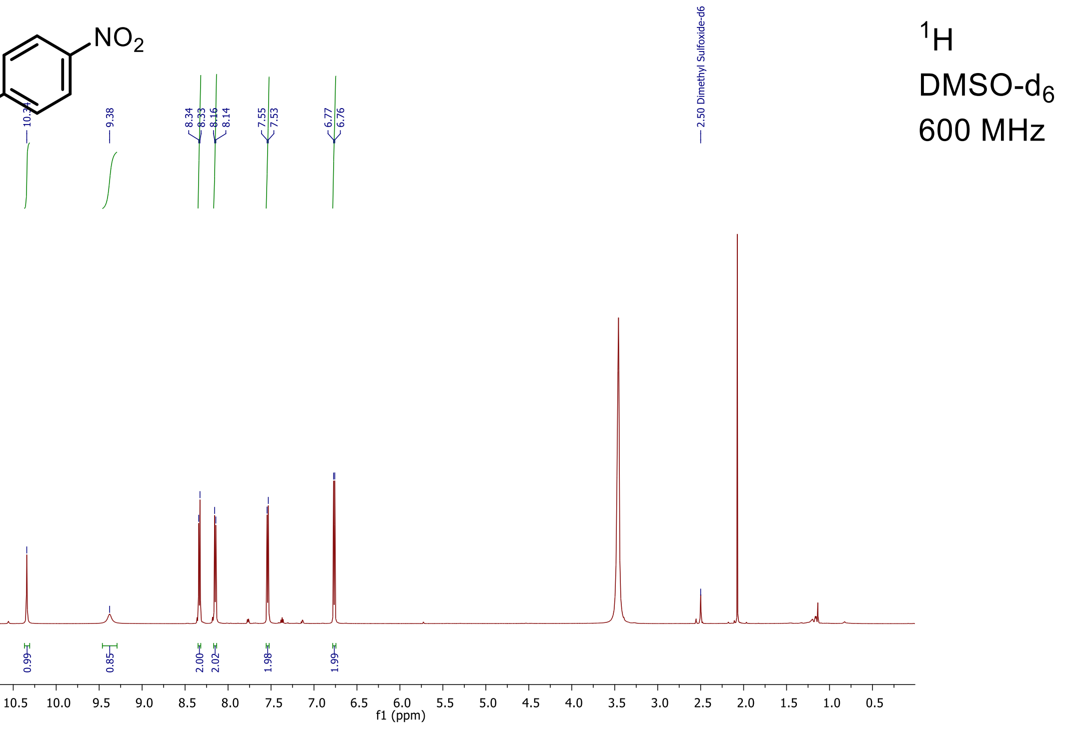

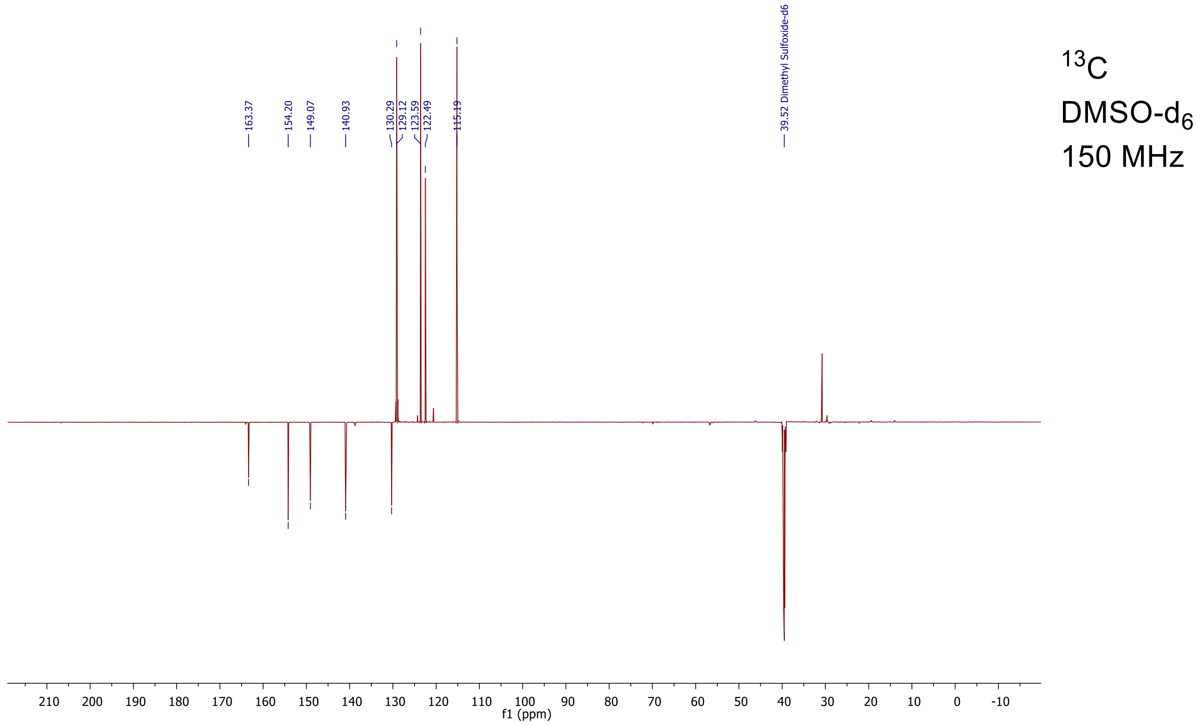

e) ***N*-(4-hydroxyphenyl)-4-nitrobenzamide (2)**:

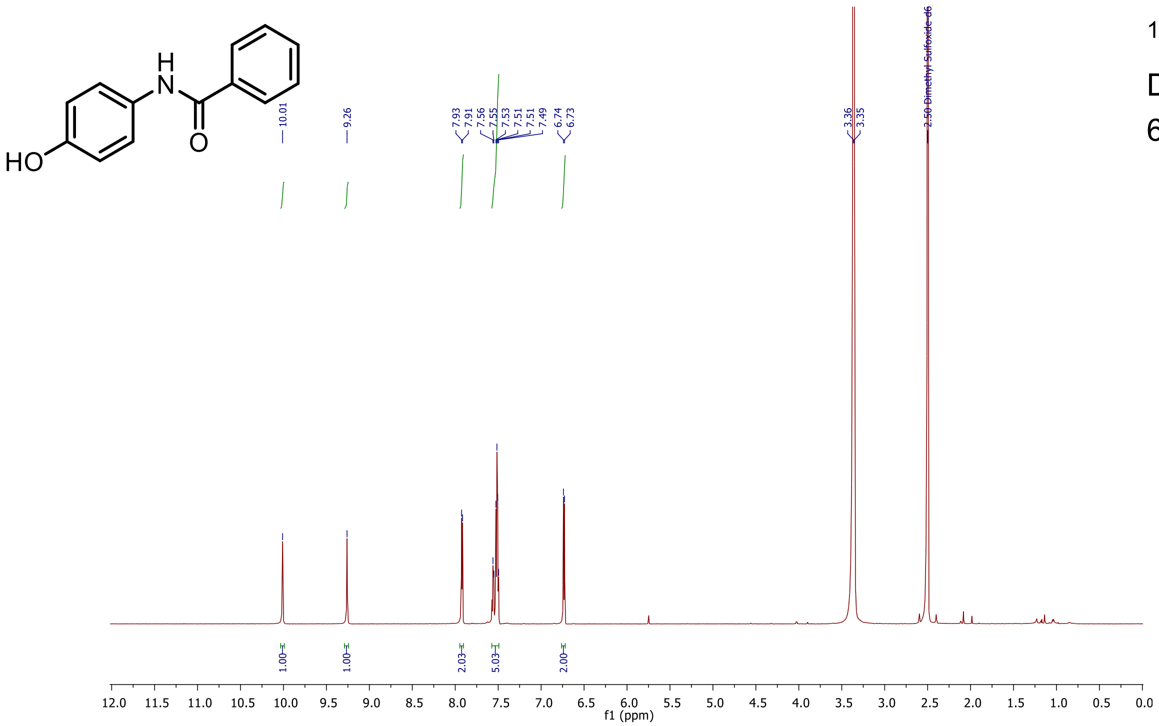

^{1}H
DMSO-d_6
600 MHz

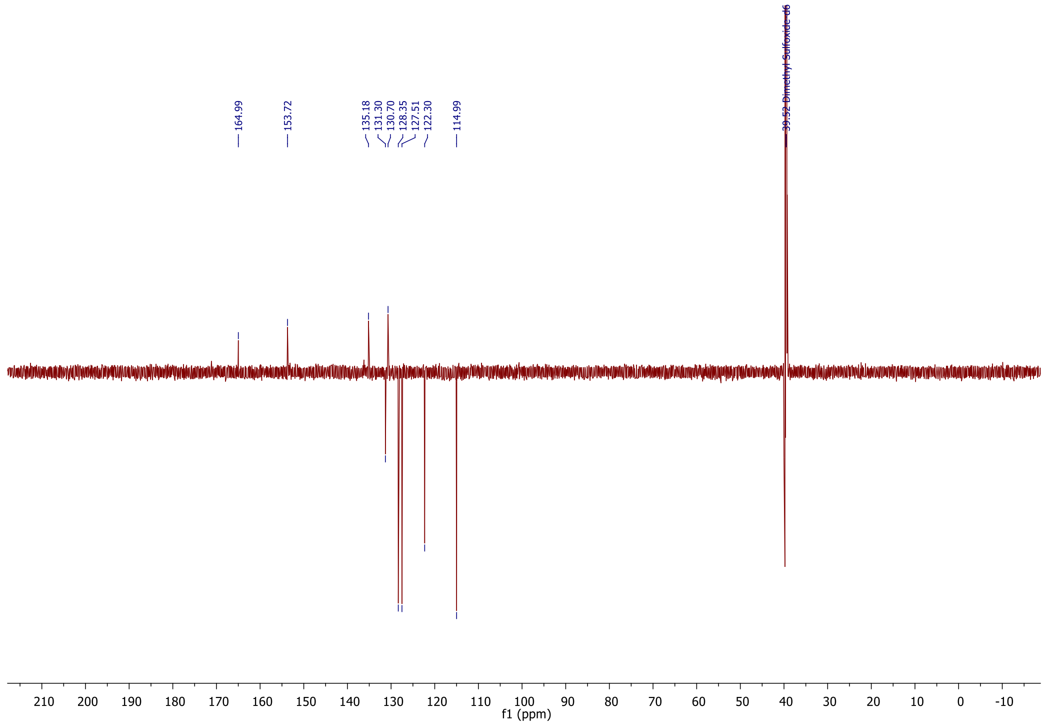

^{13}C
DMSO-d_6
150 MHz

f) ***N*-(4-hydroxyphenyl)-4-methoxybenzamide (4e)**:

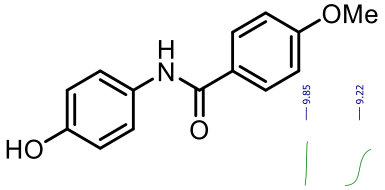

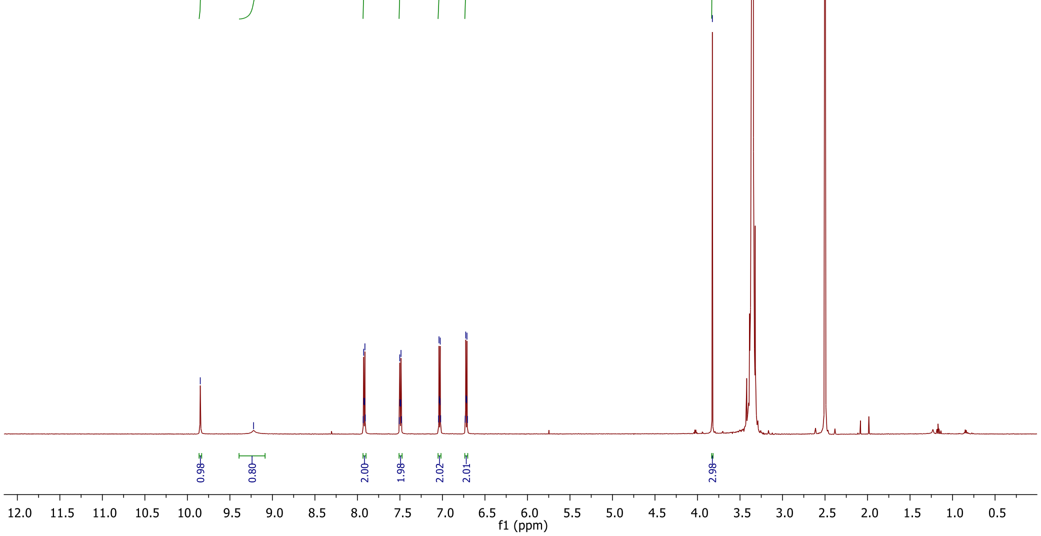

^{1}H
DMSO-d_6
600 MHz

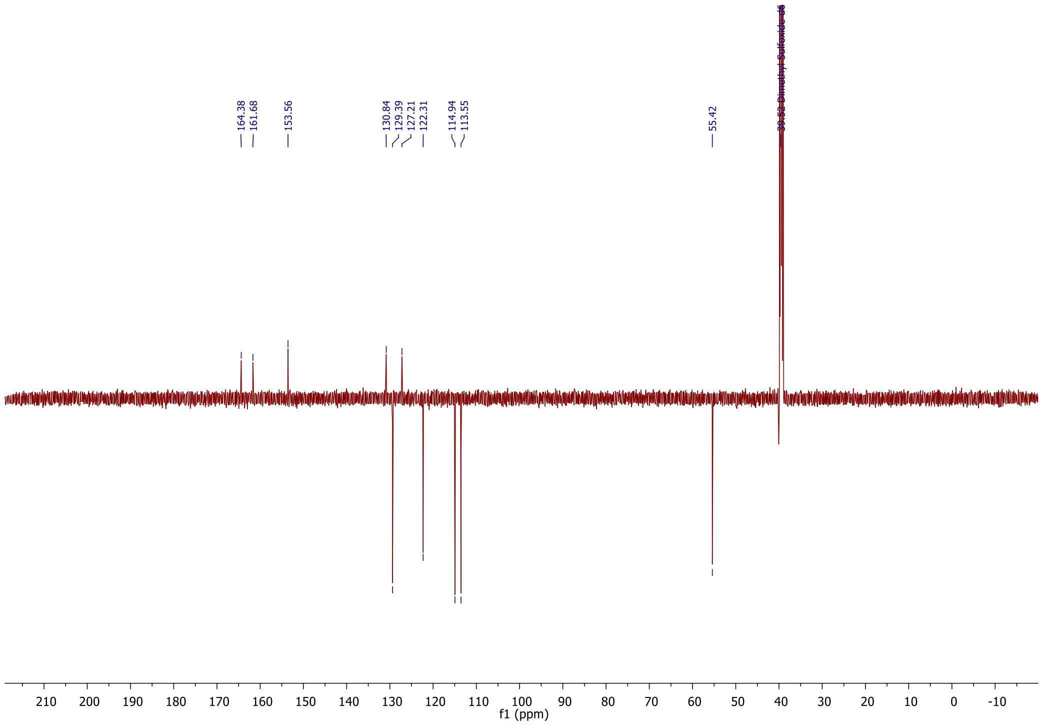

^{13}C
DMSO-d_6
150 MHz

g) ***N*-(4-hydroxy-2,3-dimethylphenyl)cinnamamide (4f)**:

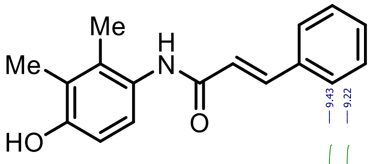

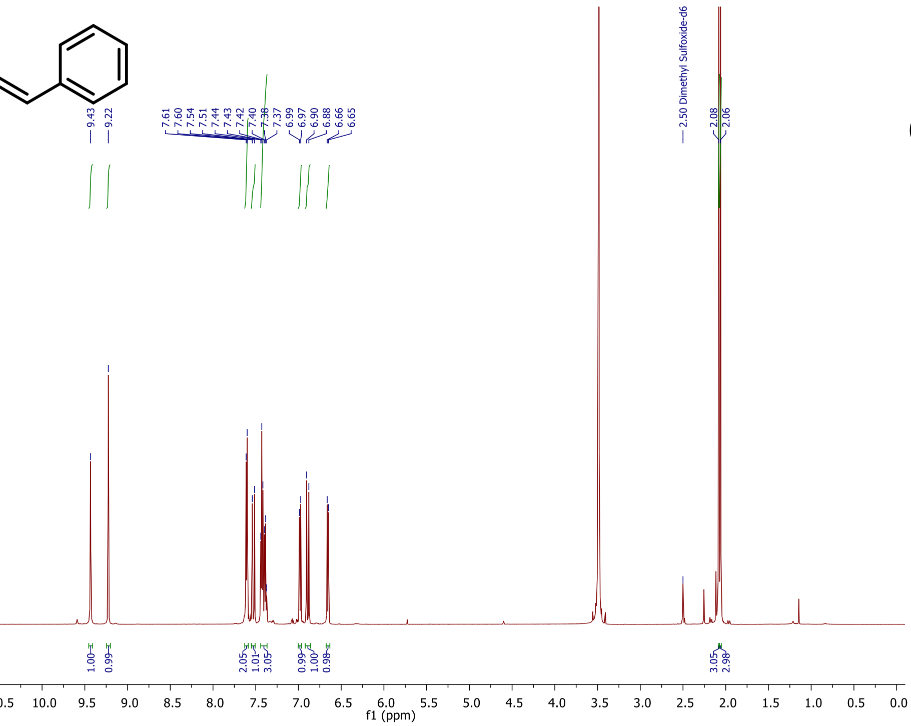

^{1}H
DMSO-d_6
600 MHz

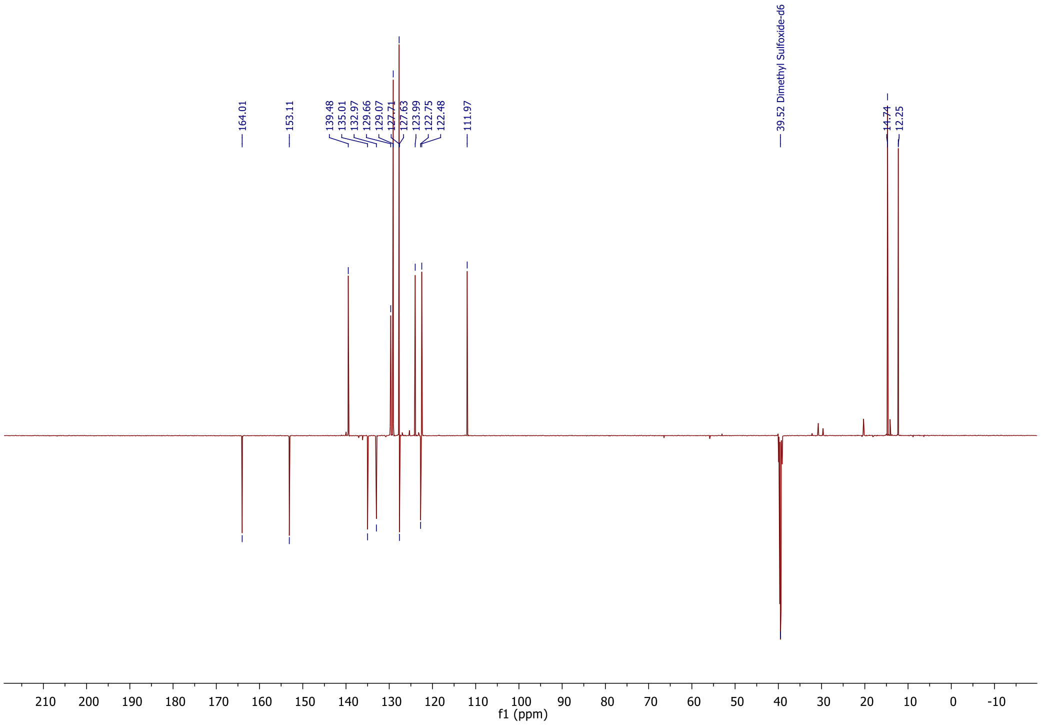

^{13}C
DMSO-d_6
150 MHz

h) ***N*-(4-hydroxyphenyl)-4-vinylbenzamide (4g)**:

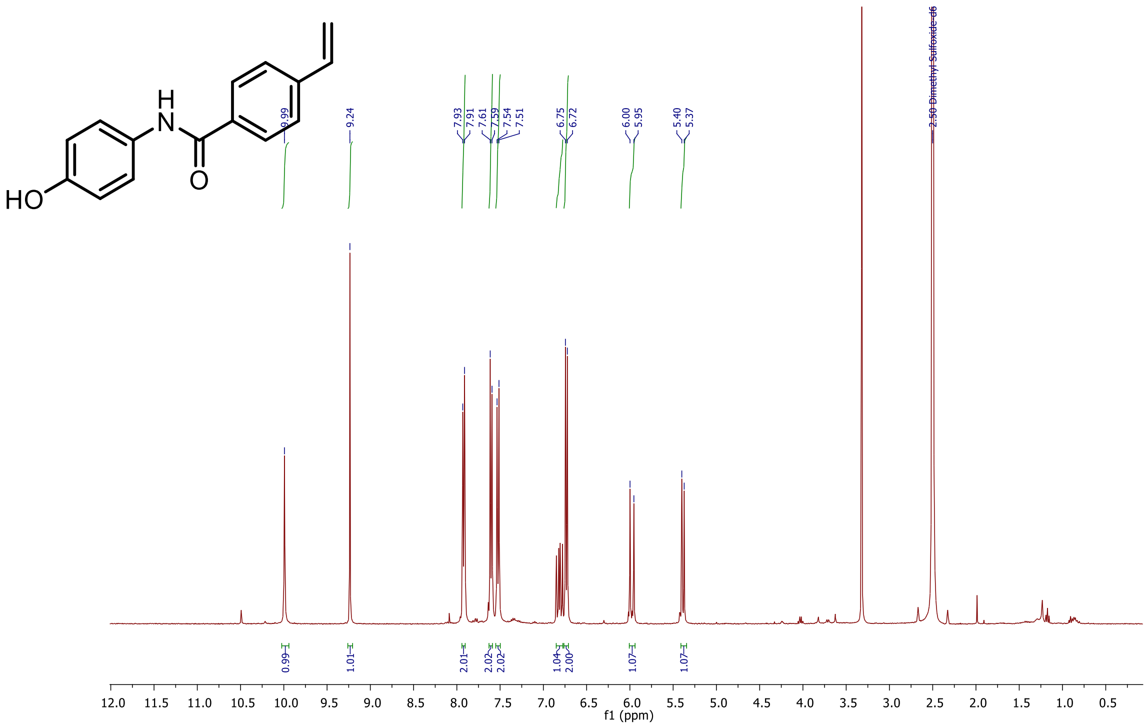

^{1}H
DMSO-d_6
400 MHz

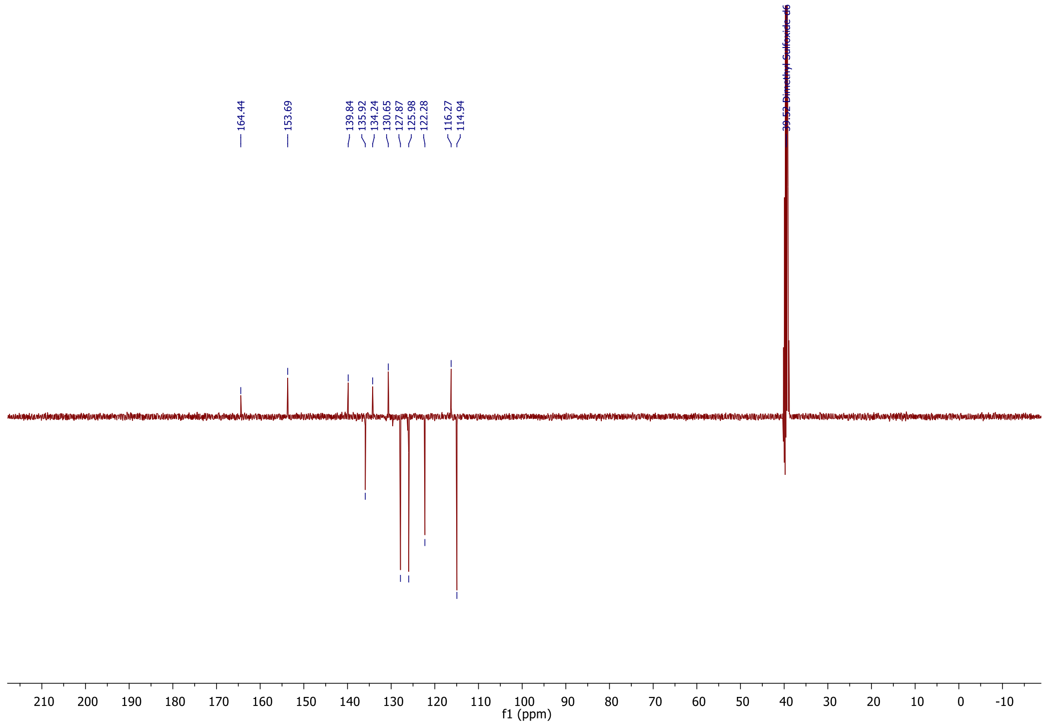

^{13}C
DMSO-d_6
100 MHz

i) ***N*-(4-hydroxyphenyl)acetamide (4h)**:

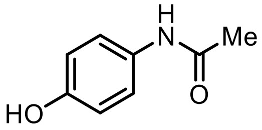

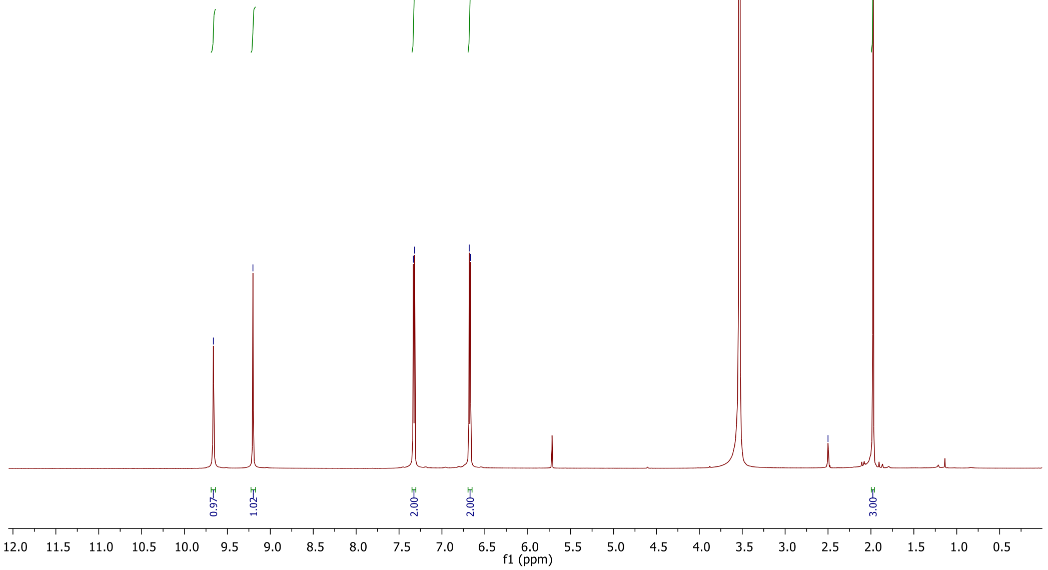

^{1}H
DMSO-d_6
600 MHz

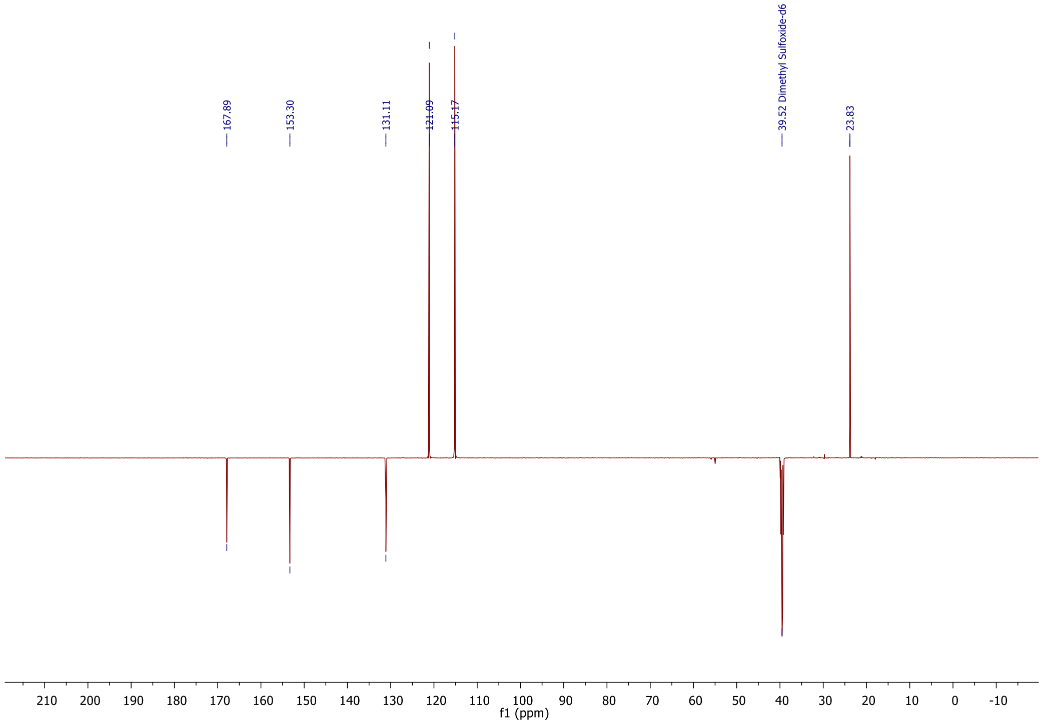

^{13}C
DMSO-d_6
150 MHz

j) ***N*-(4-hydroxynaphthalen-1-yl)acetamide (4i)**:

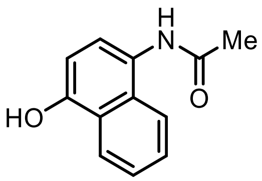

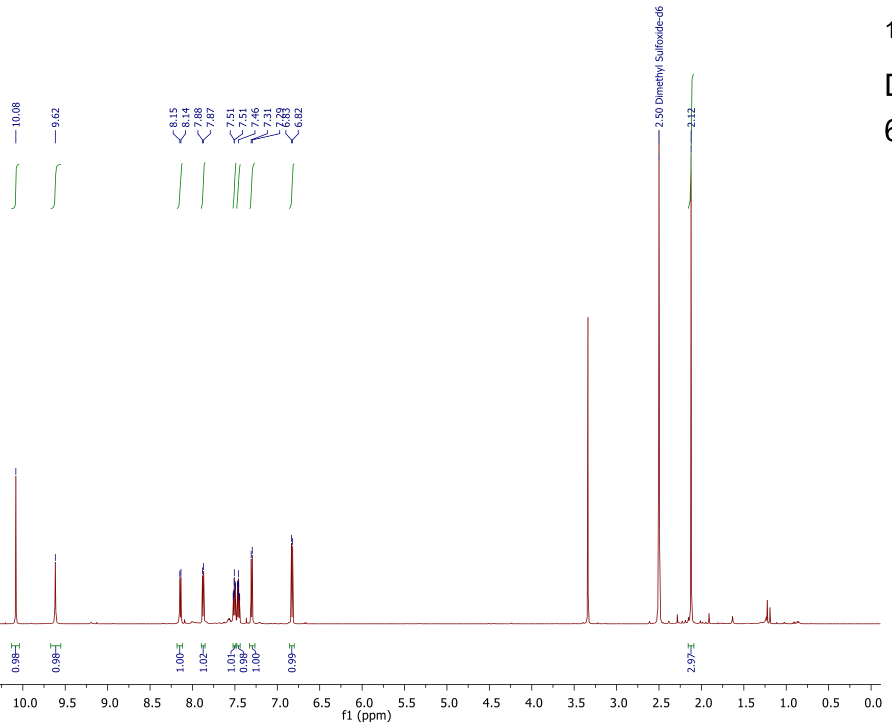

^{1}H
DMSO-d_6
600 MHz

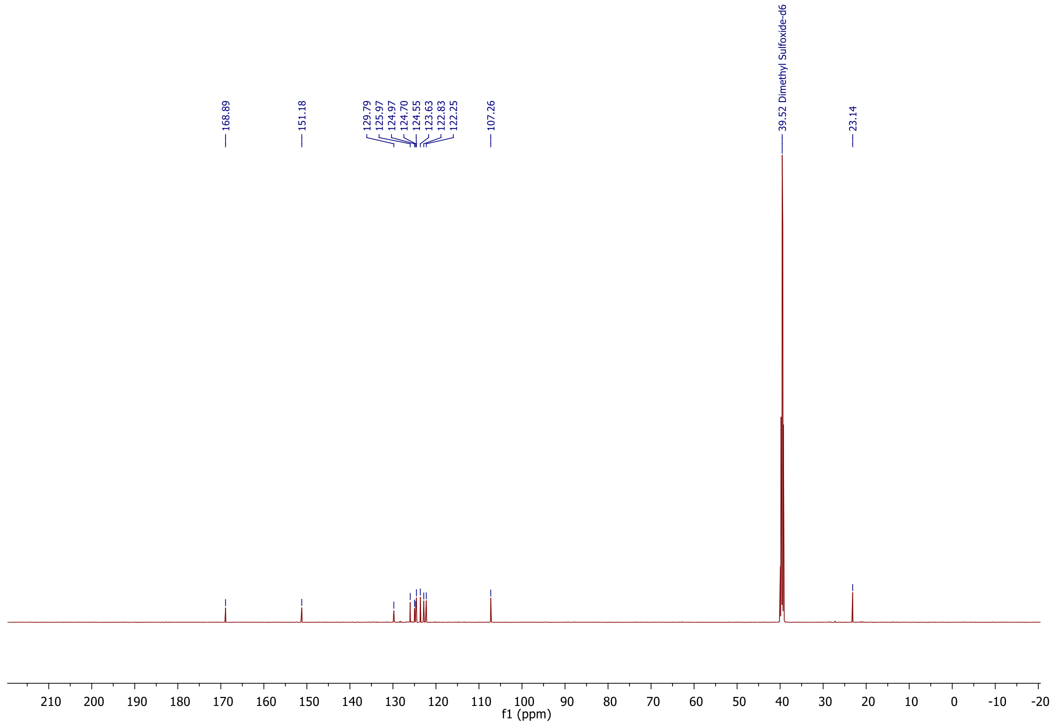

^{13}C
DMSO-d_6
150 MHz

k) ***N*-(4-hydroxy-3-methylphenyl)acetamide (4j)**:

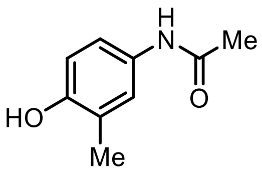

^1H
MeOH-d_4
600 MHz

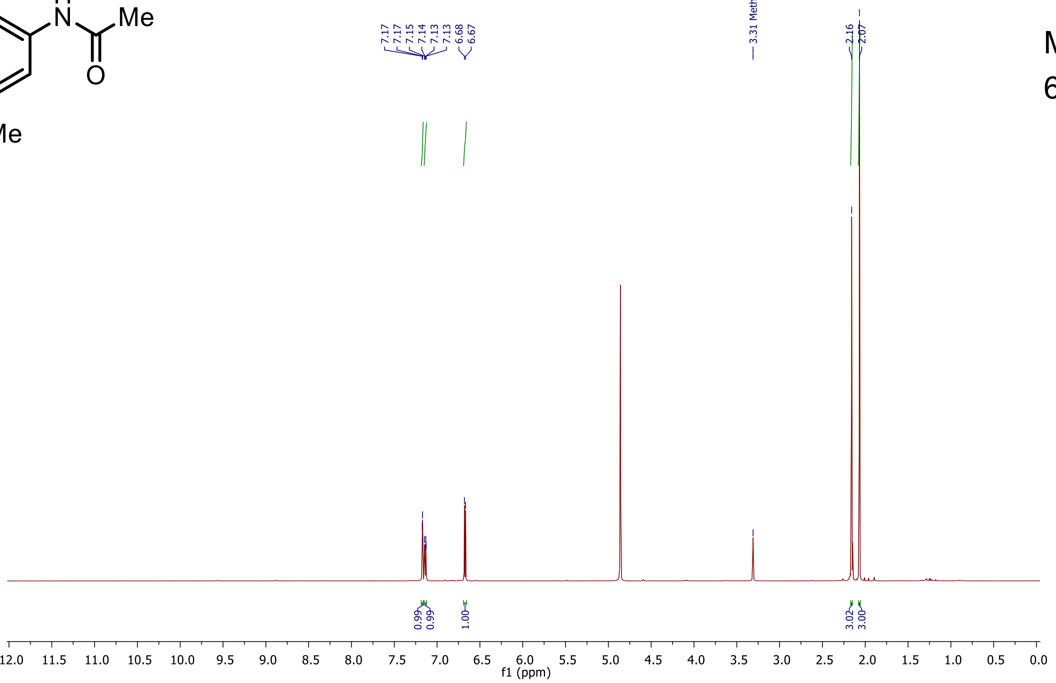

^{13}C
MeOH-d_4
150 MHz

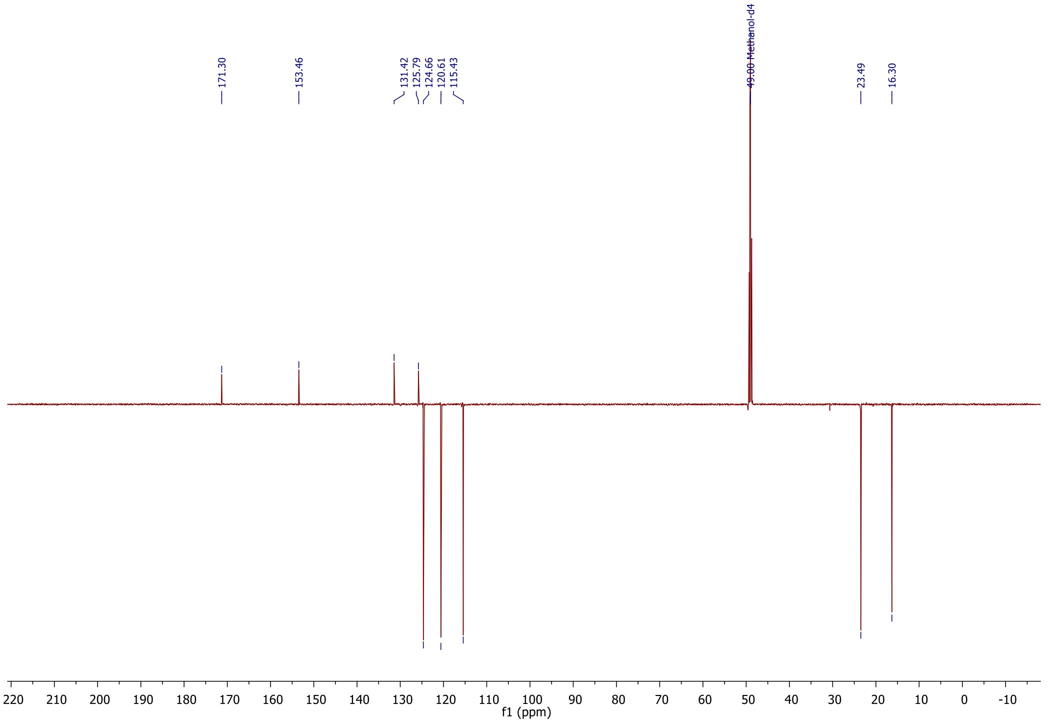

l) ***N*-(4-hydroxy-2,6-dimethylphenyl)acetamide (4k)**:

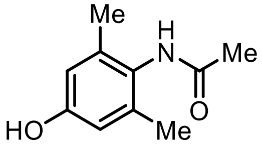

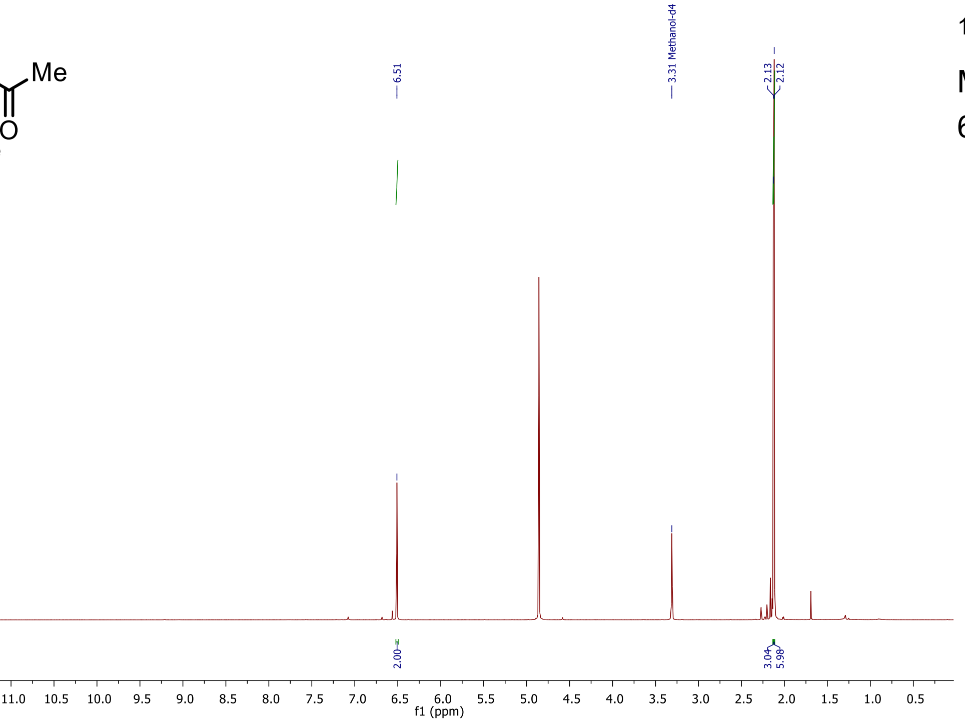

^{1}H
MeOH-d_4
600 MHz

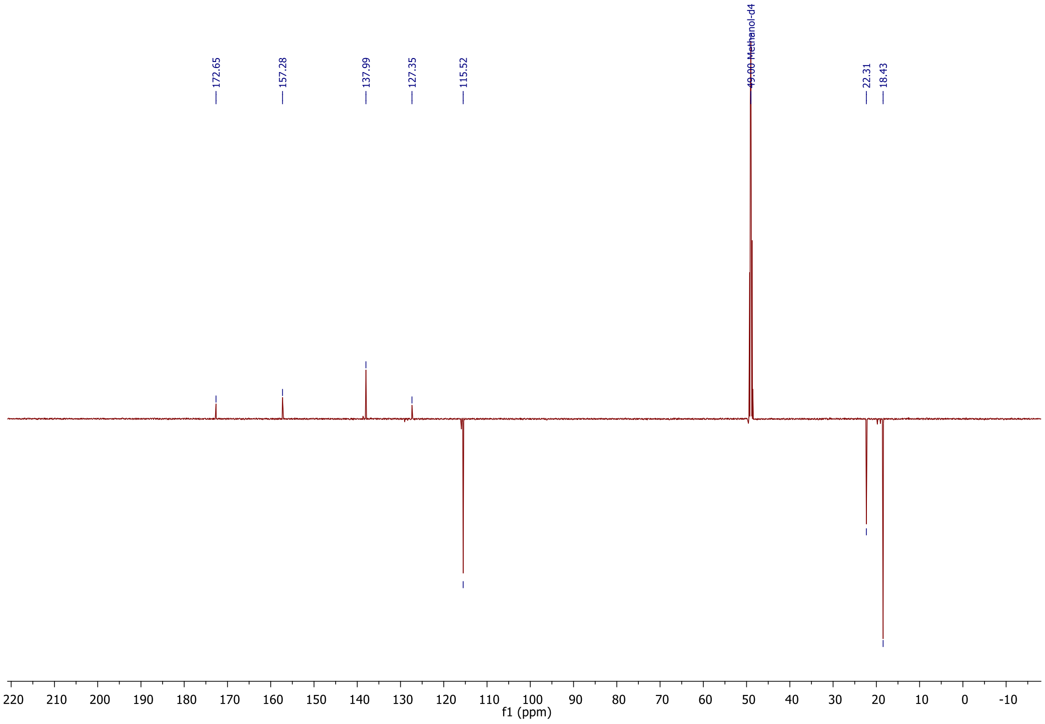

^{13}C
MeOH-d_4
150 MHz

m) *N*-(4-hydroxy-3,5-dimethylphenyl)acetamide (4l):

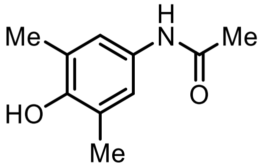

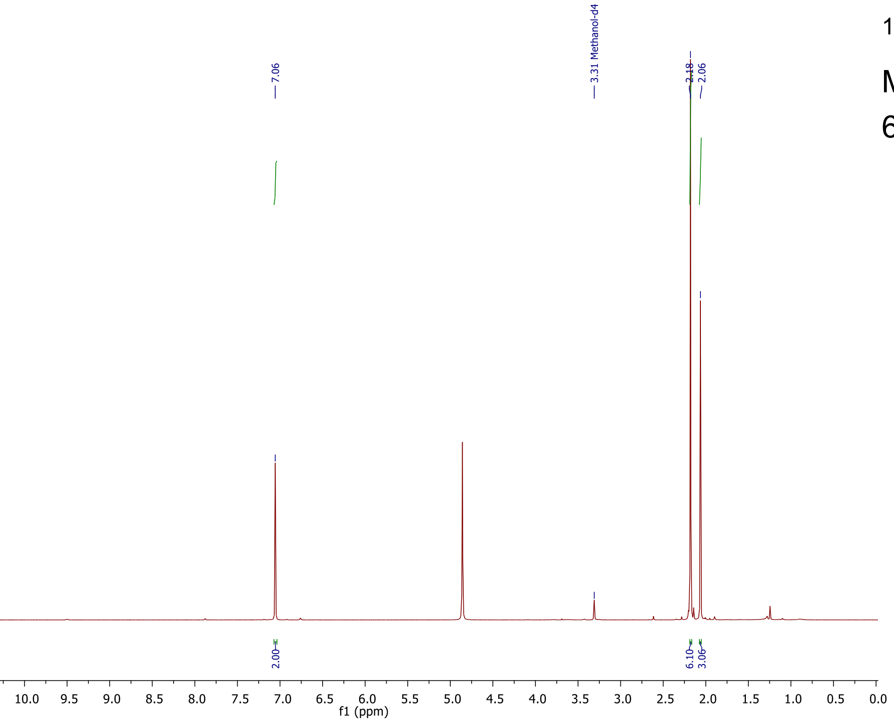

^{1}H
MeOH-d_4
600 MHz

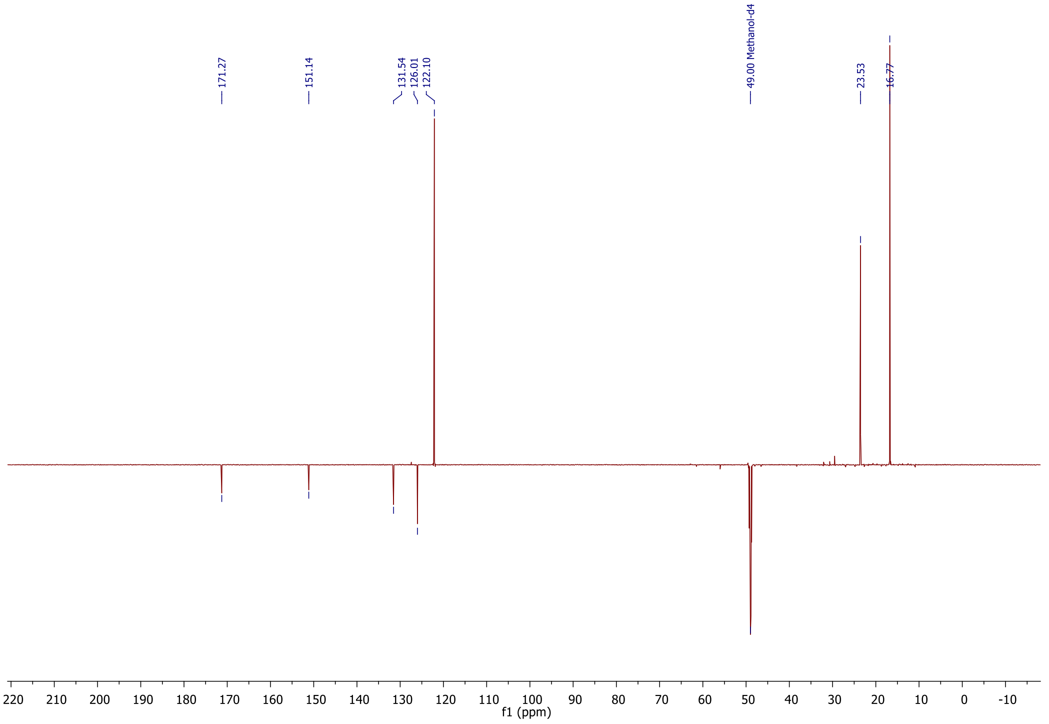

^{13}C
MeOH-d_4
150 MHz

n) *N*-(4-hydroxy-3-methoxylphenyl)acetamide (4m):

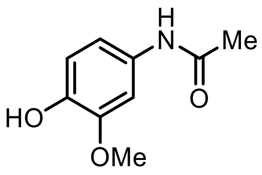

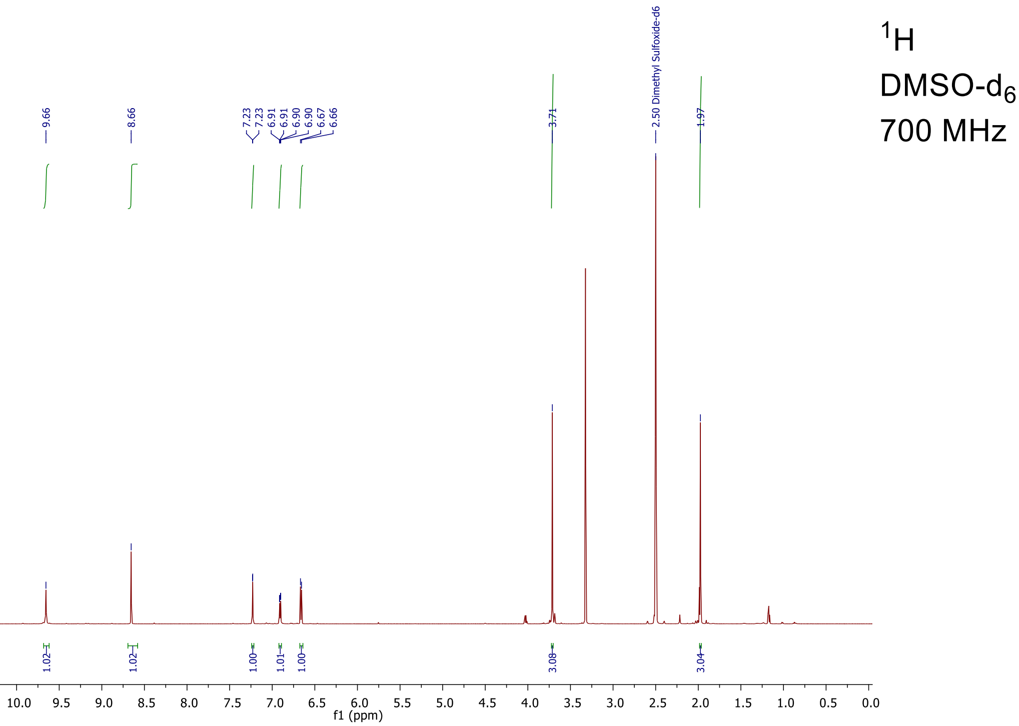

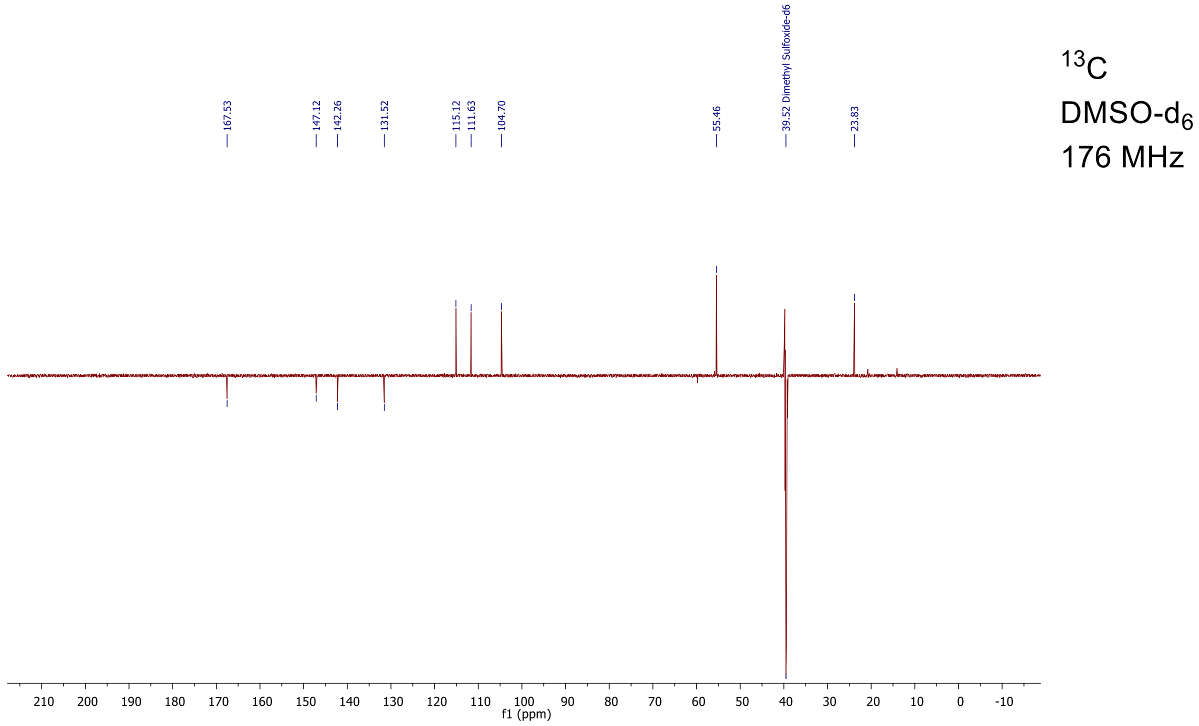

o) ***N*-(3-acetyl-4-hydroxyphenyl)acetamide (4n)**:

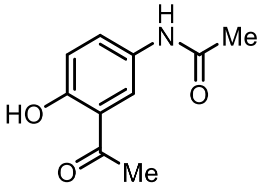

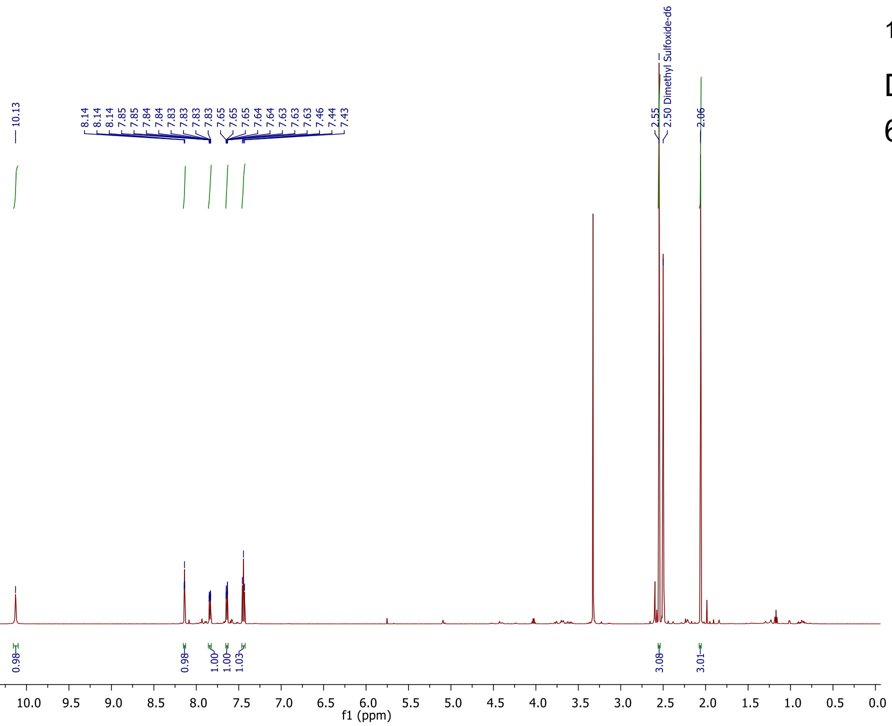

^{1}H
DMSO-d_6
600 MHz

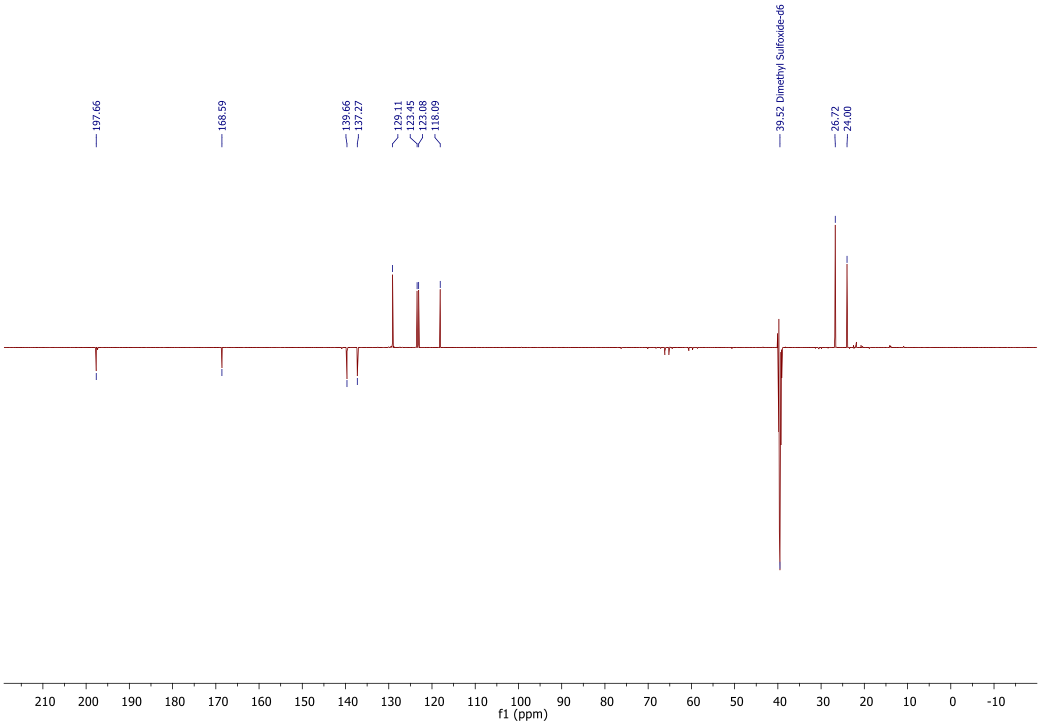

^{13}C
DMSO-d6
150 MHz

p) ***N*-(3-fluoro-4-hydroxy-5-iodophenyl)acetamide (4o)**:

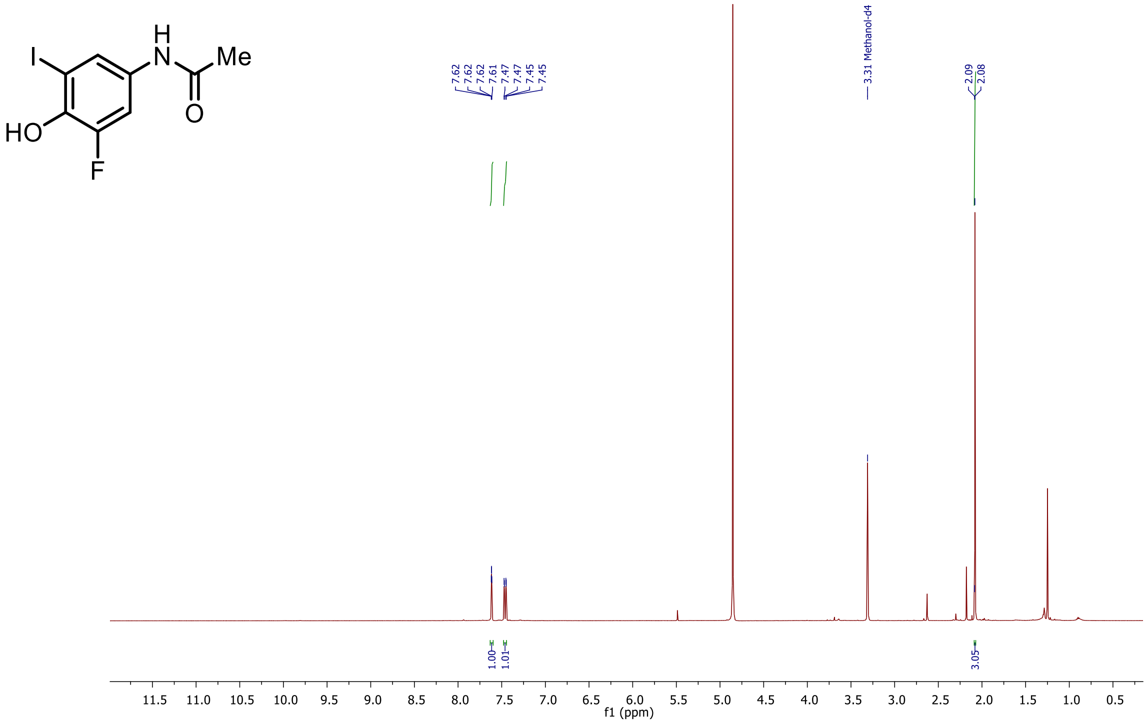

^{1}H
MeOH-d_4
600 MHz

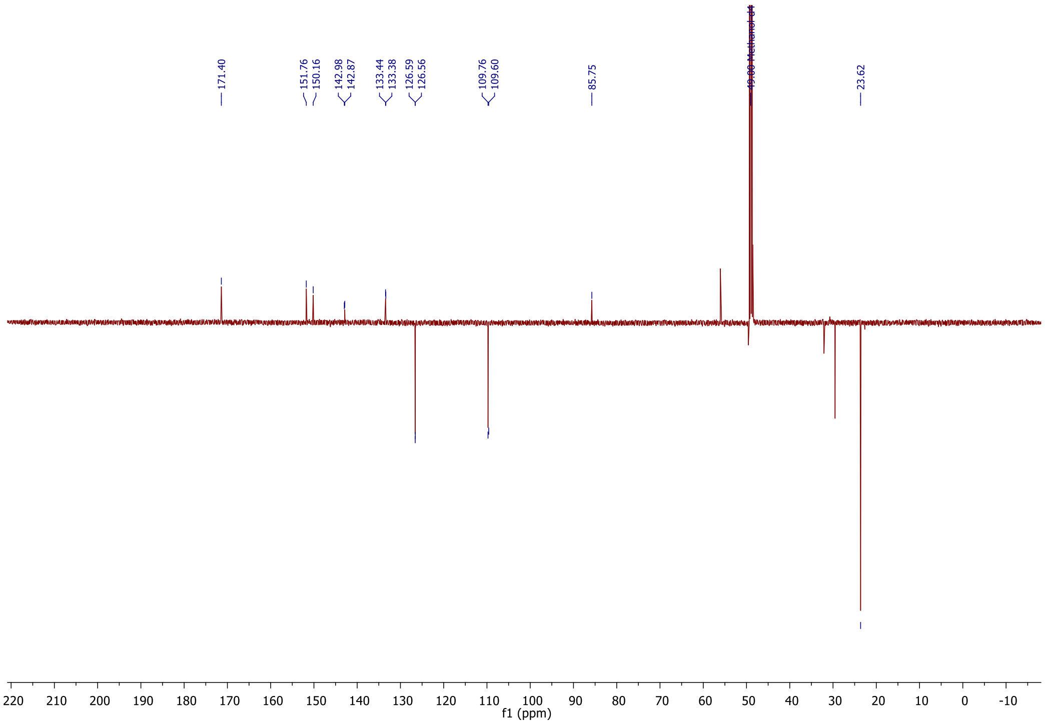

^{13}C
MeOH-d_4
150 MHz

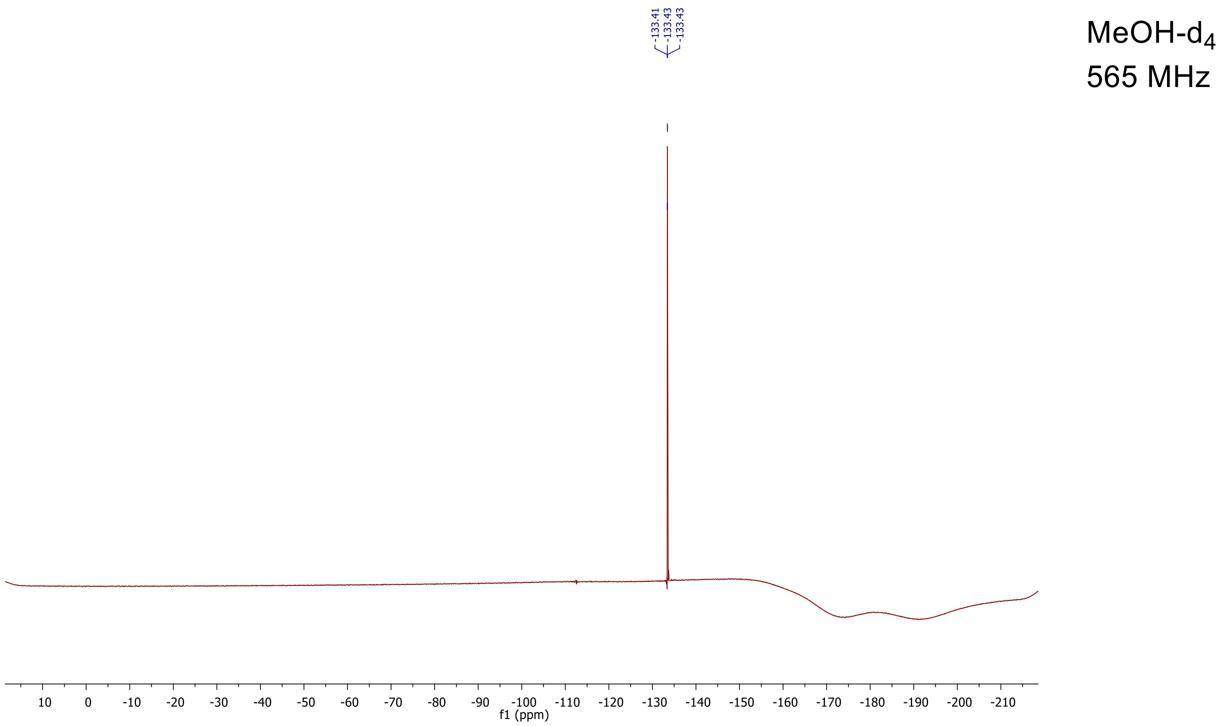

-133.41
-133.43
-133.43
^{19}F
MeOH-d4
565 MHz
10 0 -10 -20 -30 -40 -50 -60 -70 -80 -90 -100 -110 -120 -130 -140 -150 -160 -170 -180 -190 -200 -210
f1 (ppm)

q) ***N*-(3-fluoro-4-hydroxyphenyl)acetamide (4p)**:

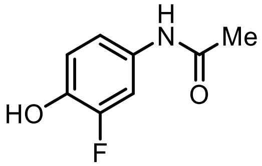

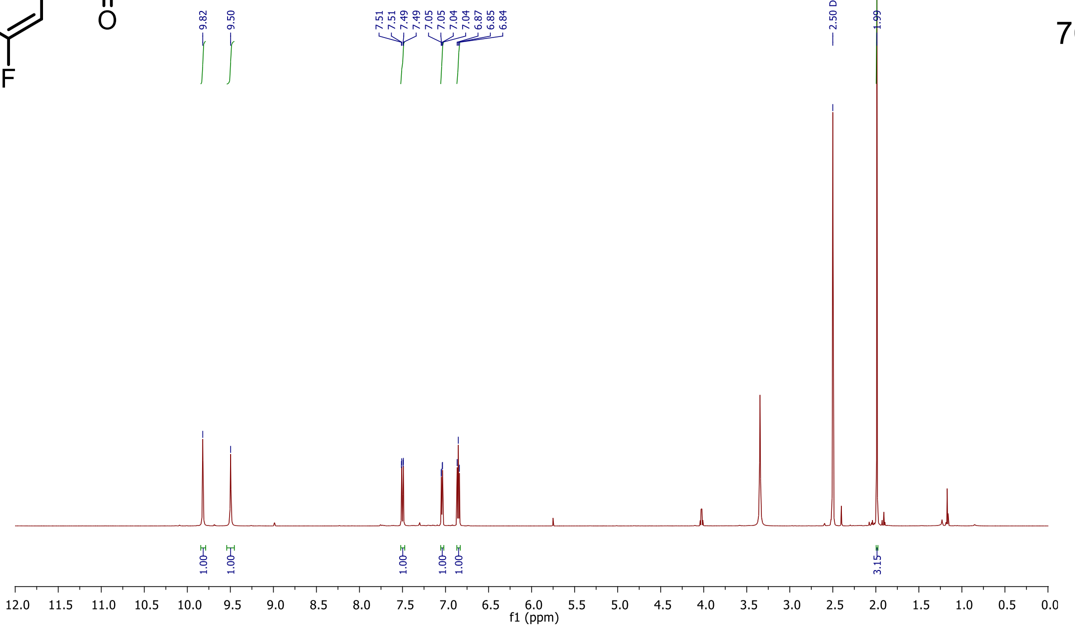

^{1}H
DMSO-d_6
700 MHz

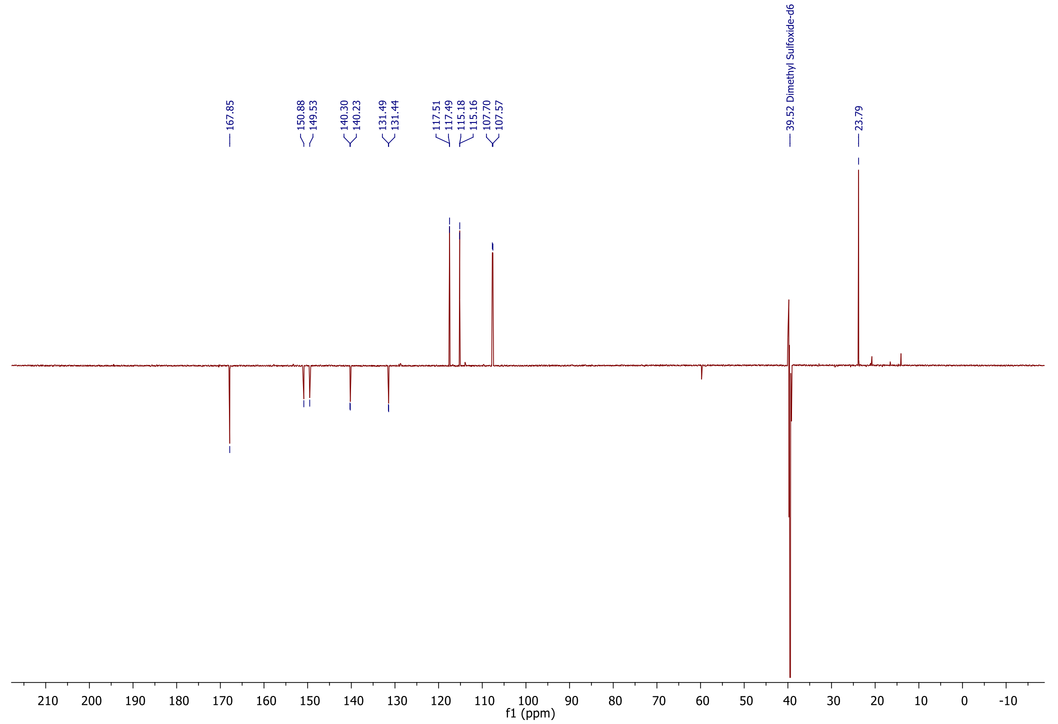

^{13}C
DMSO-d_6
176 MHz

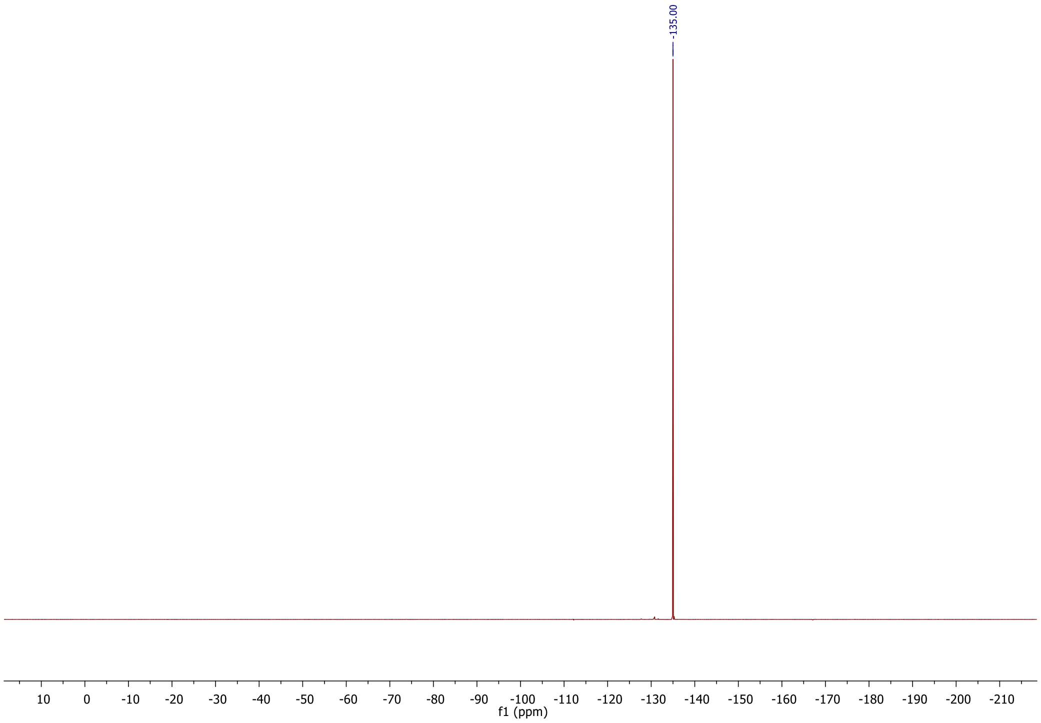

^{19}F
DMSO-d_6
659 MHz

r) ***N*-(2-fluoro-4-hydroxyphenyl)acetamide (4q)**:

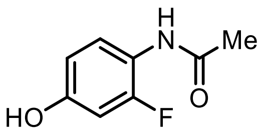

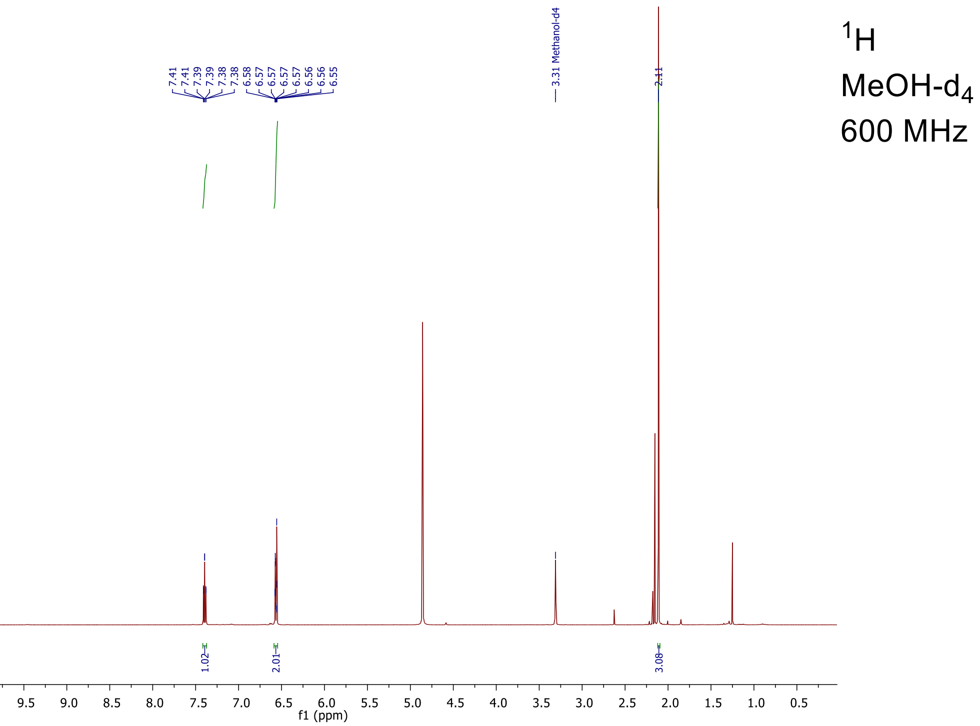

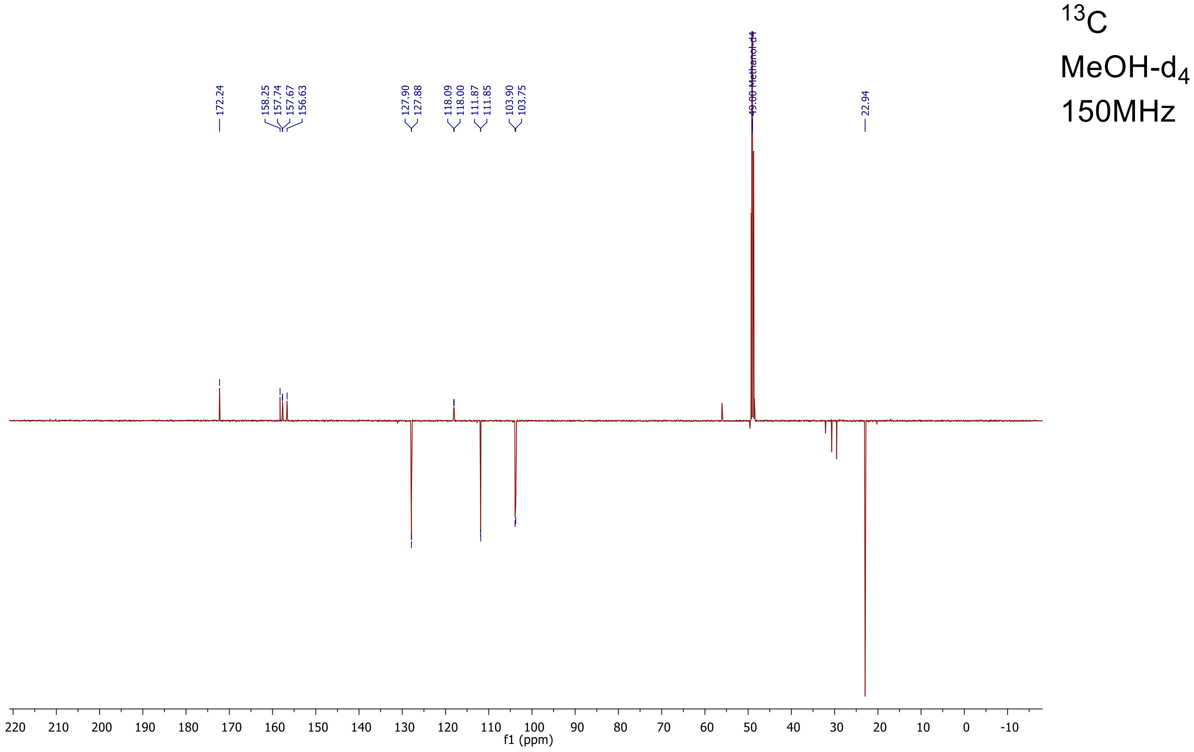

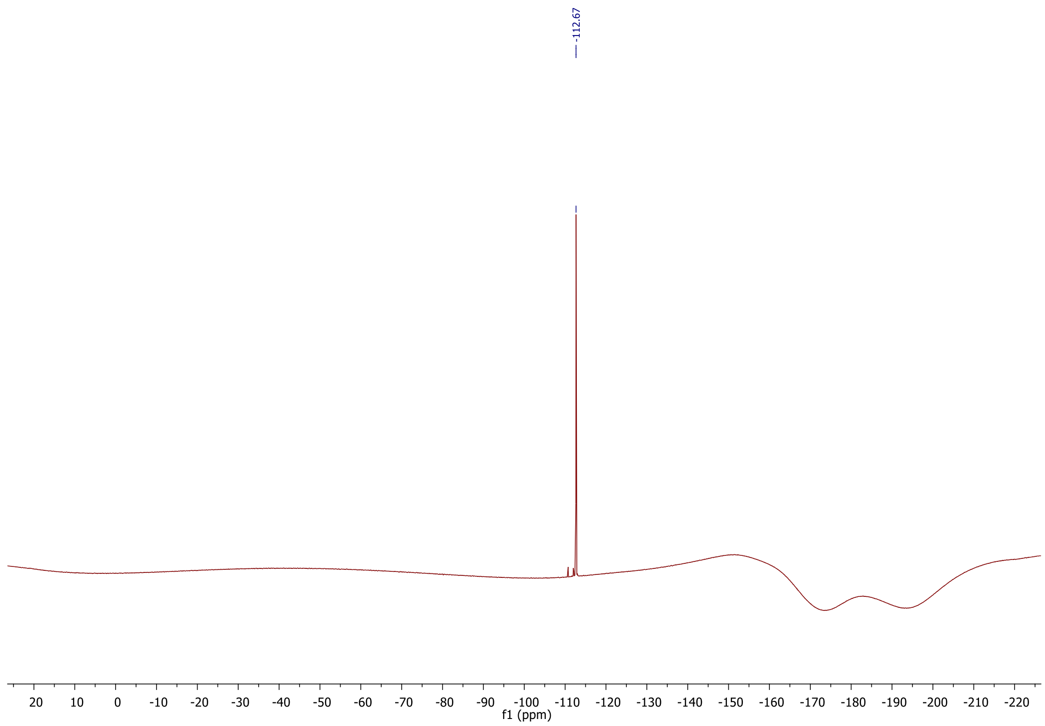

^{19}F
MeOH-d_4
470 MHz

s) ***N*-(4-hydroxy-3-(trifluoromethyl)phenyl)acetamide (4r)**:

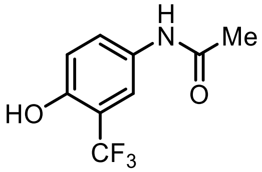

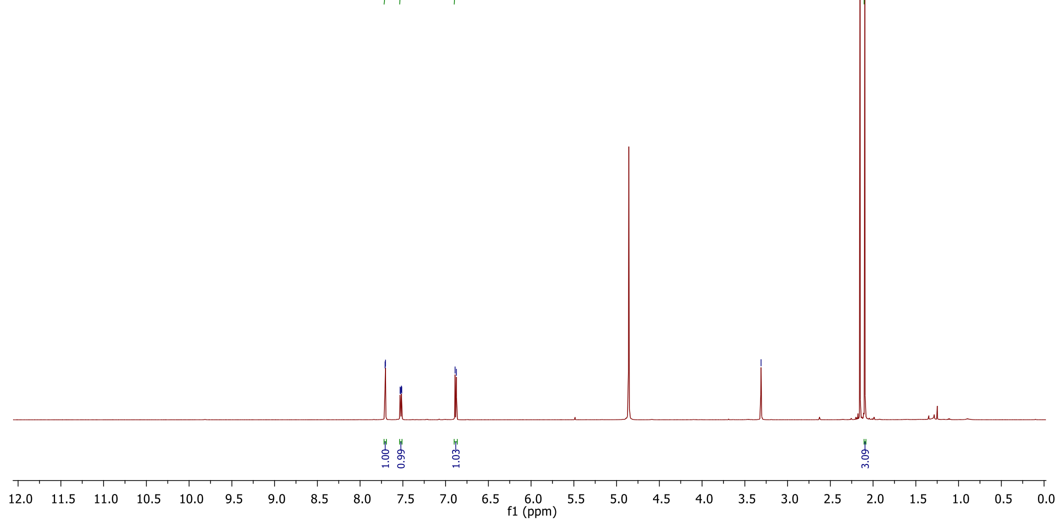

^{1}H
MeOH-d_4
600 MHz

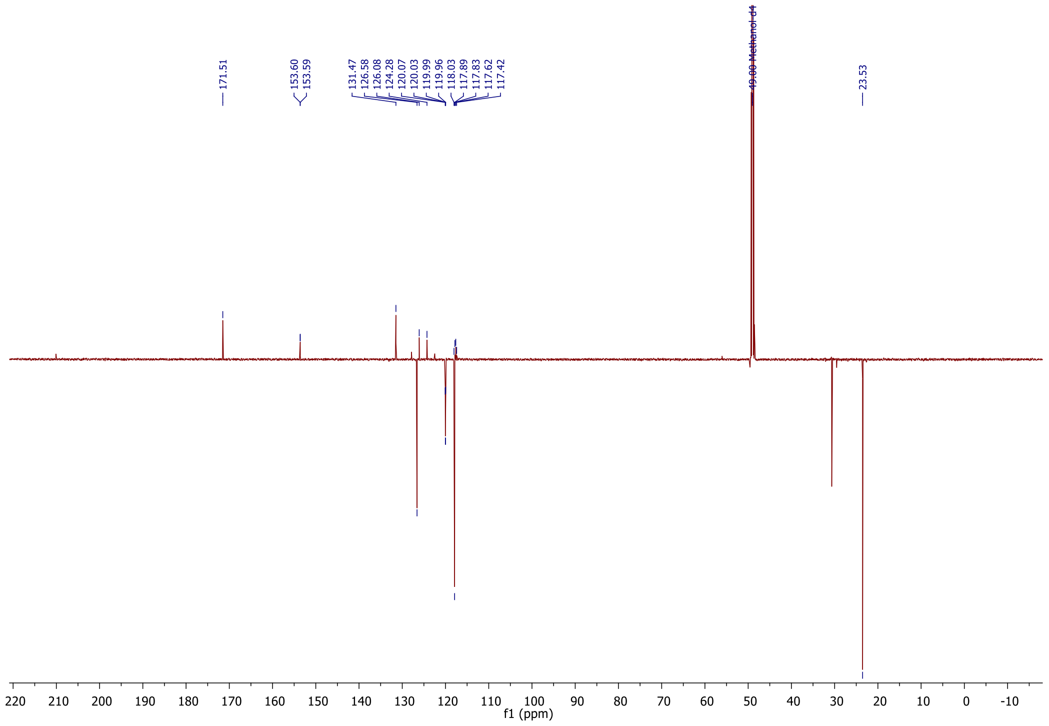

^{13}C
MeOH-d_4
150MHz

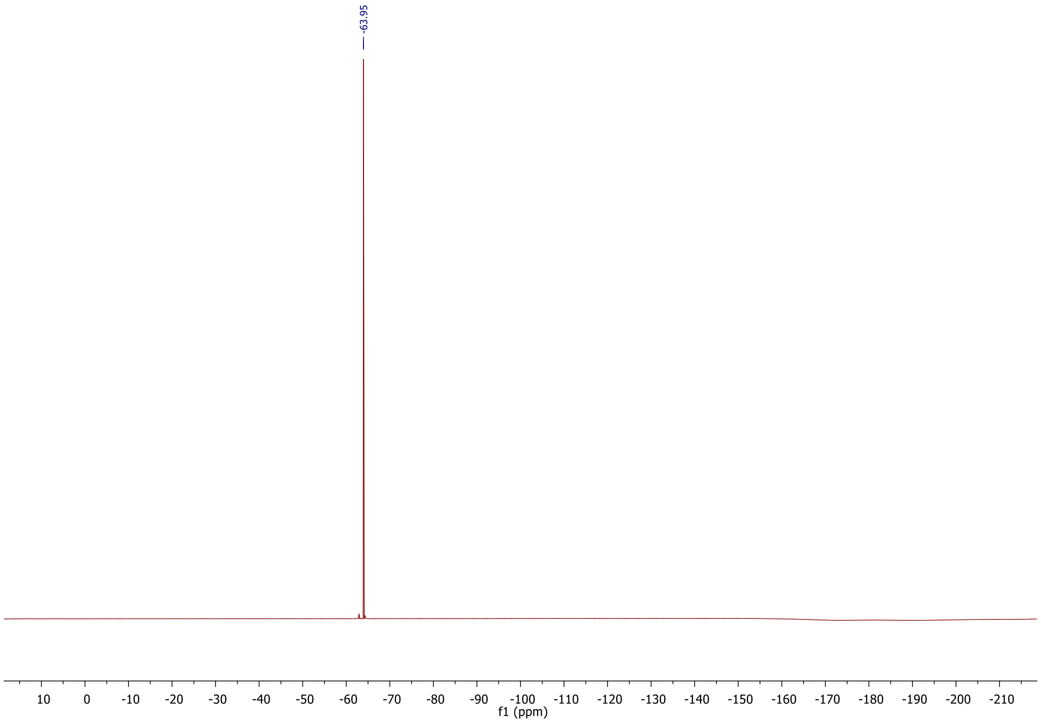

^{19}F
MeOH-d_4
565 MHz

t) ***N*-(3-bromo-4-hydroxy-2-methylphenyl)acetamide (4s)**:

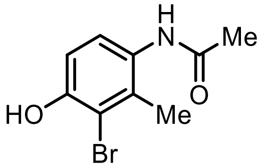

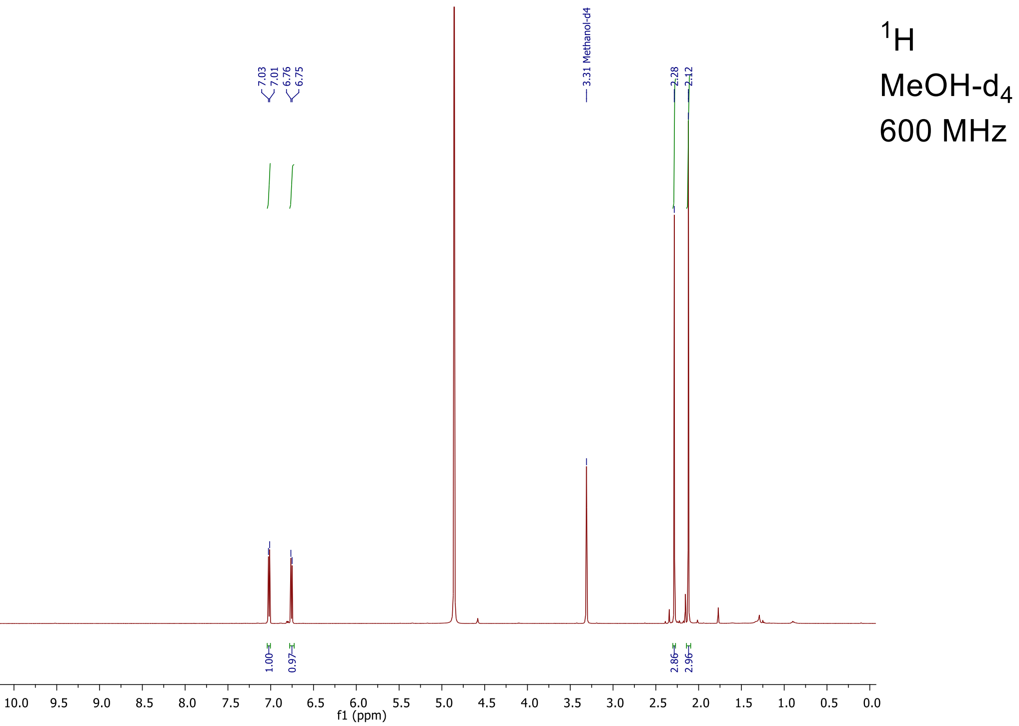

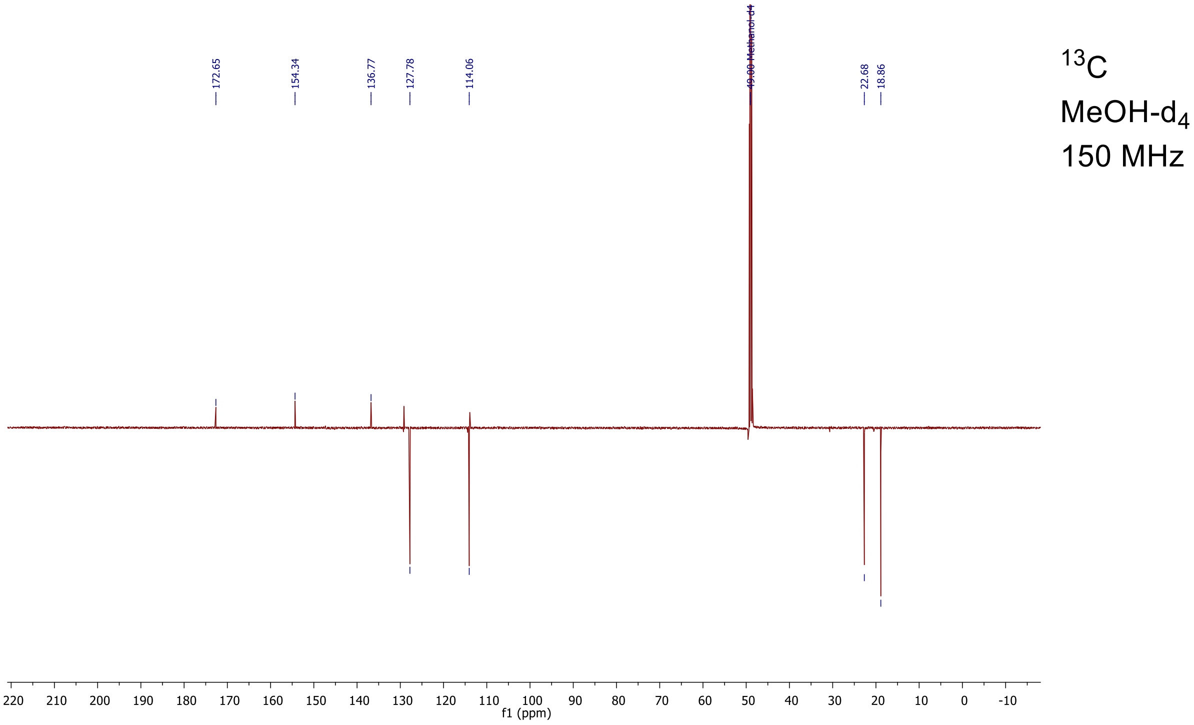

u) ***N*-(4-hydroxy-2-iodophenyl)acetamide (4t)**:

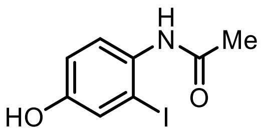

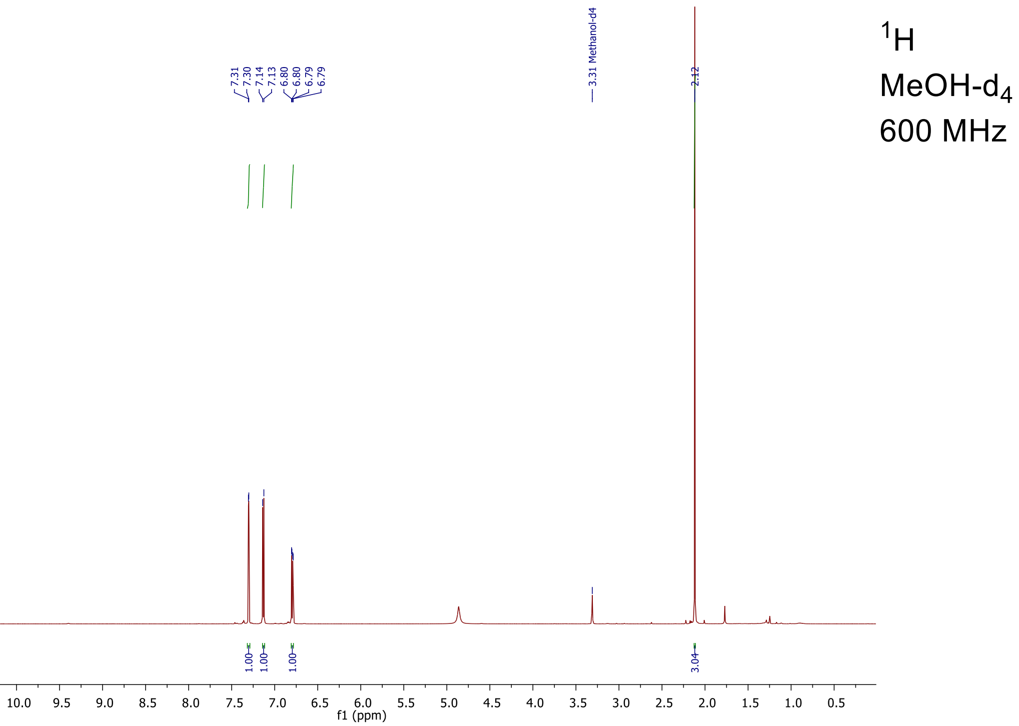

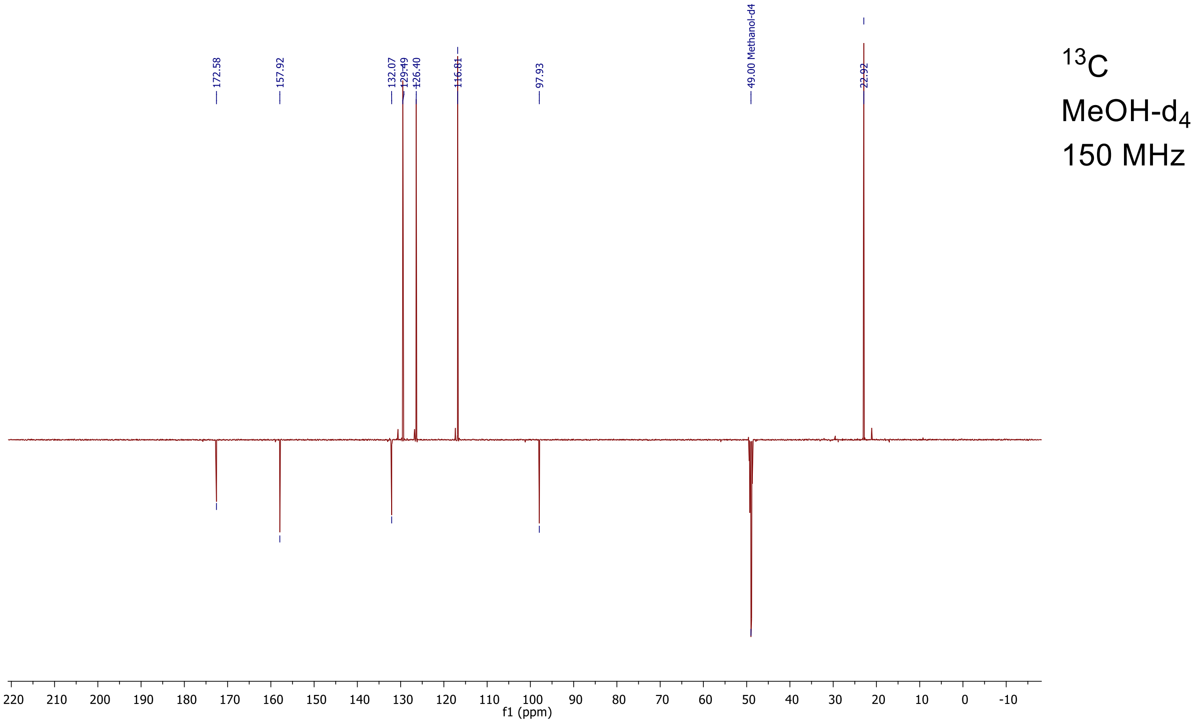

v) **Practolol (5)**:

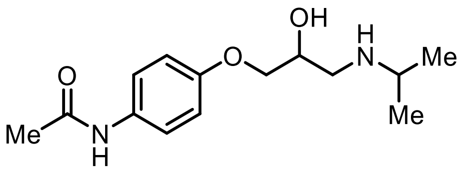

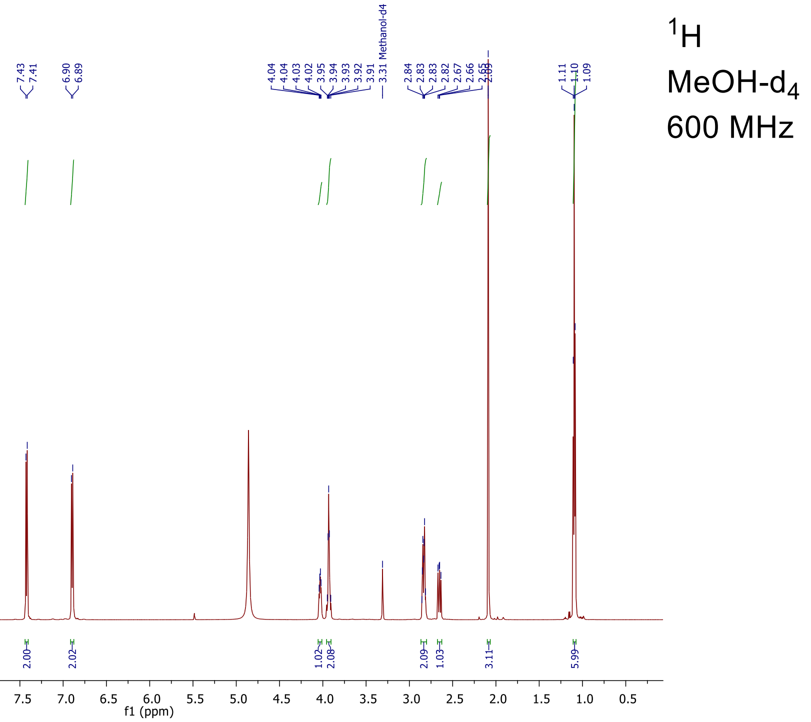

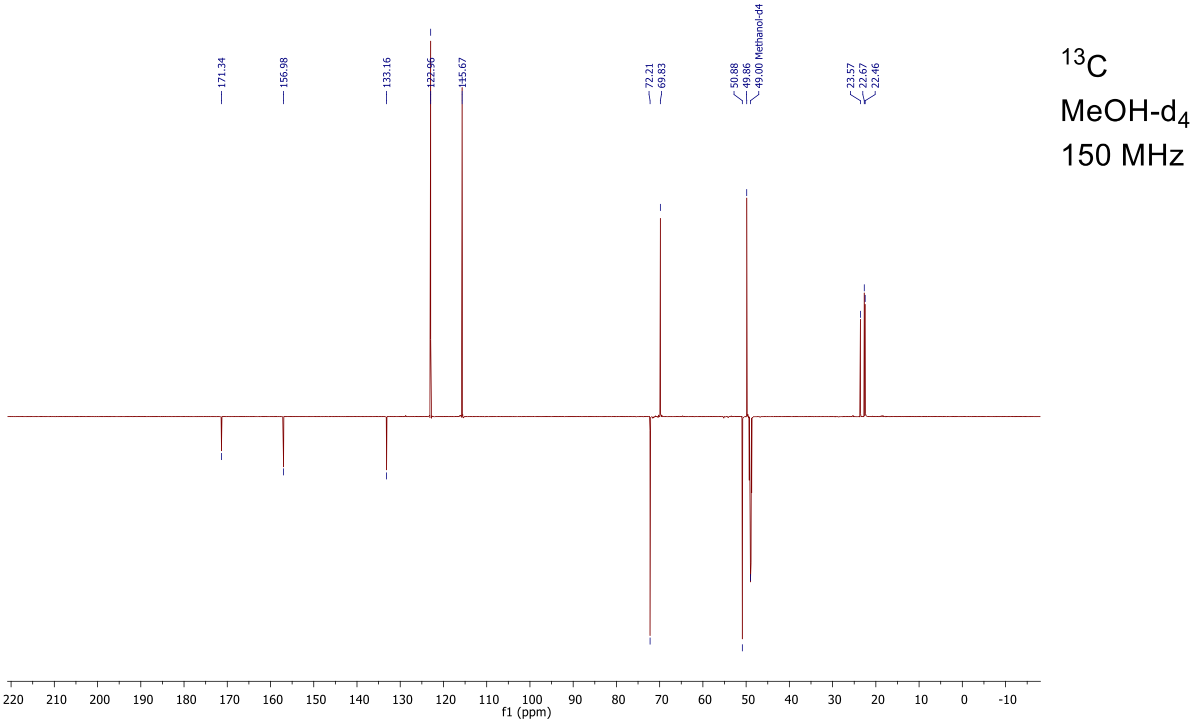

w) 2,2-dichloro-*N*-(4-hydroxyphenyl)acetamide (7):

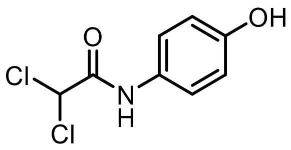

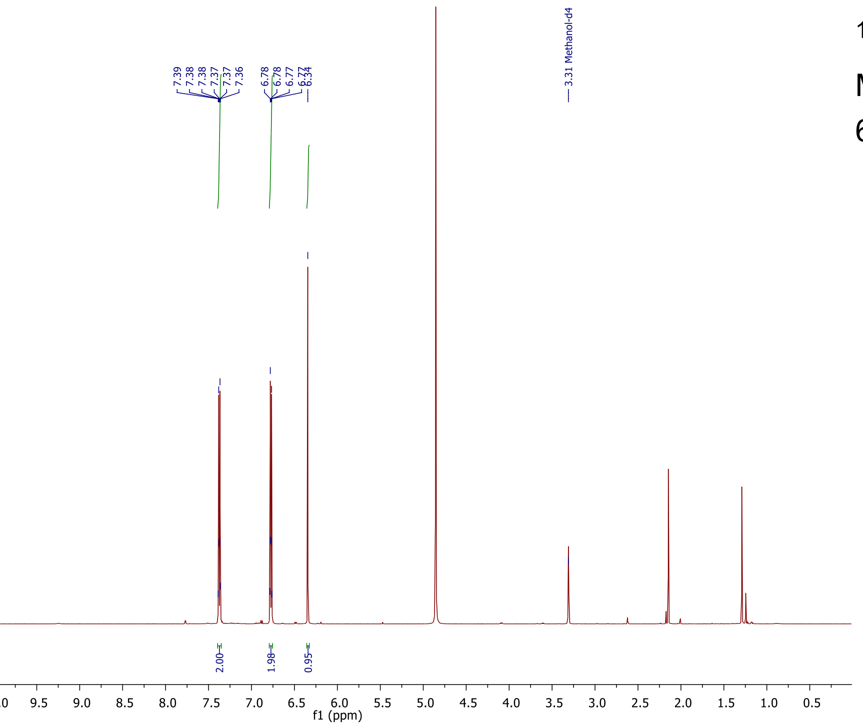

^1H
MeOH-d_4
600 MHz

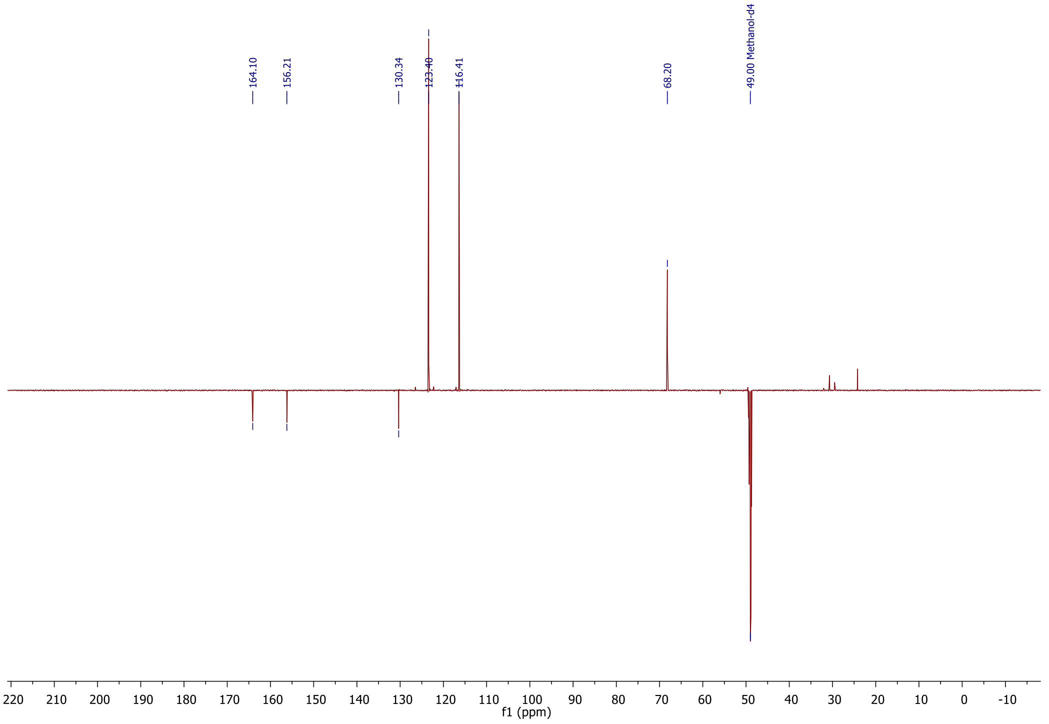

^{13}C
MeOH-d_4
150 MHz

x) 4-(2,2-dichloroacetamido)phenyl furan-2-carboxylate (7a):

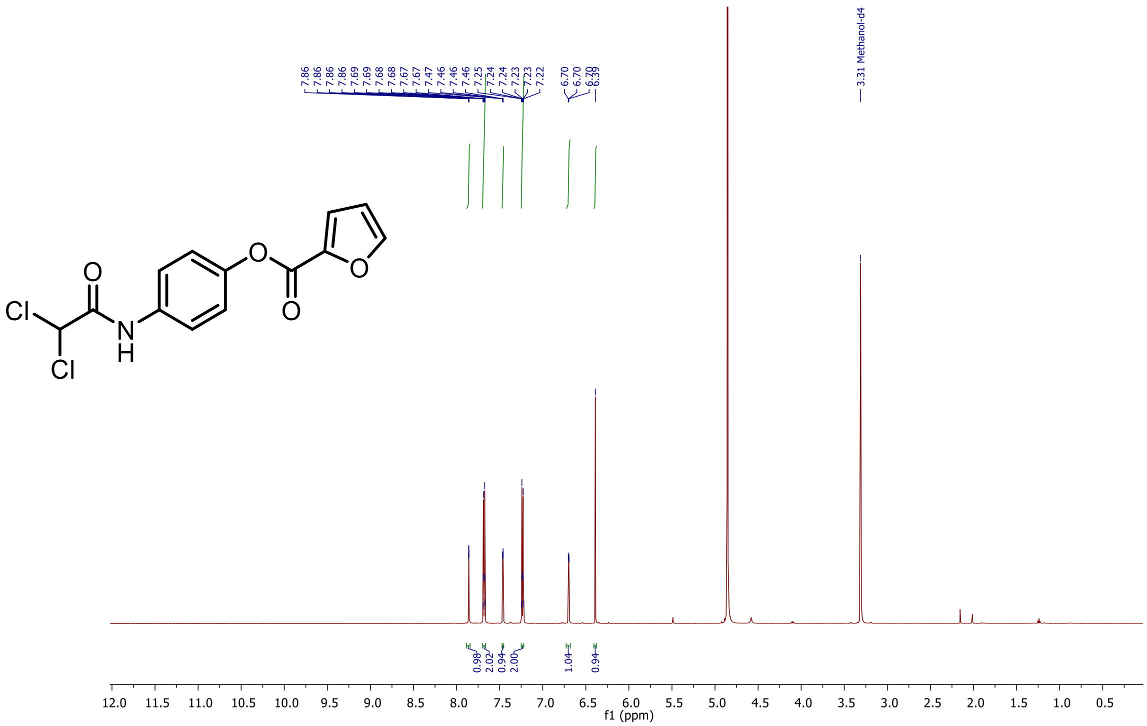

^{1}H
MeOH-d_4
600 MHz

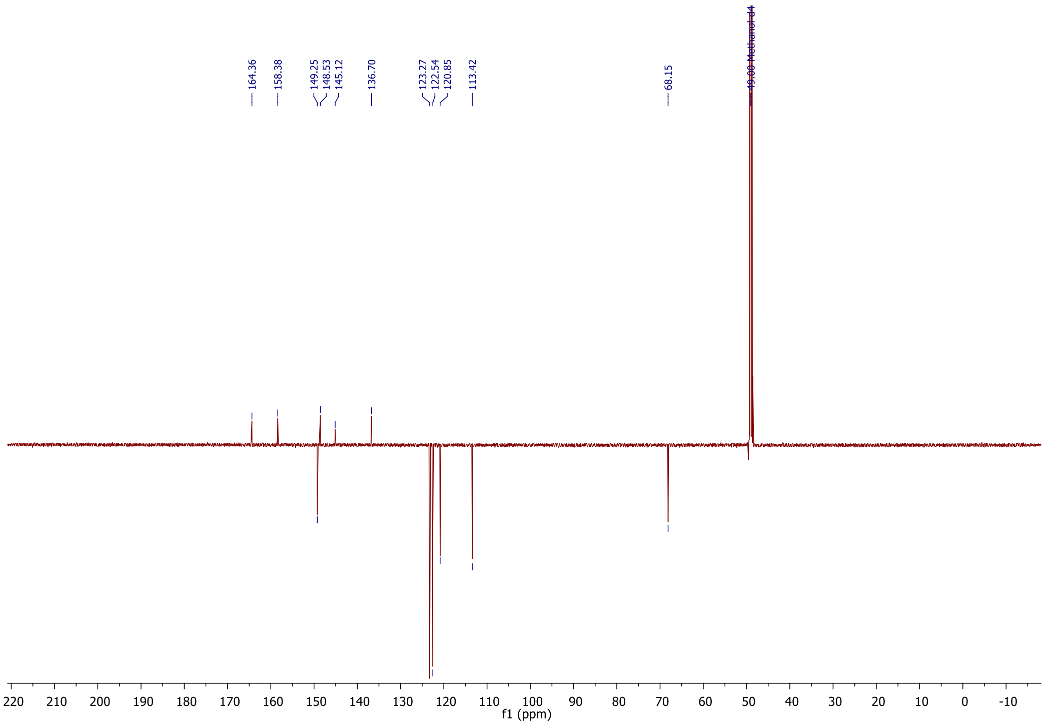

^{13}C
MeOH-d_4
150 MHz

y) Diloxanide Furoate (8):

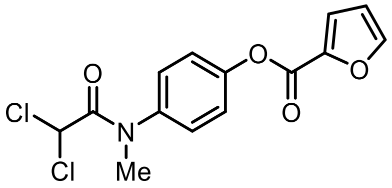

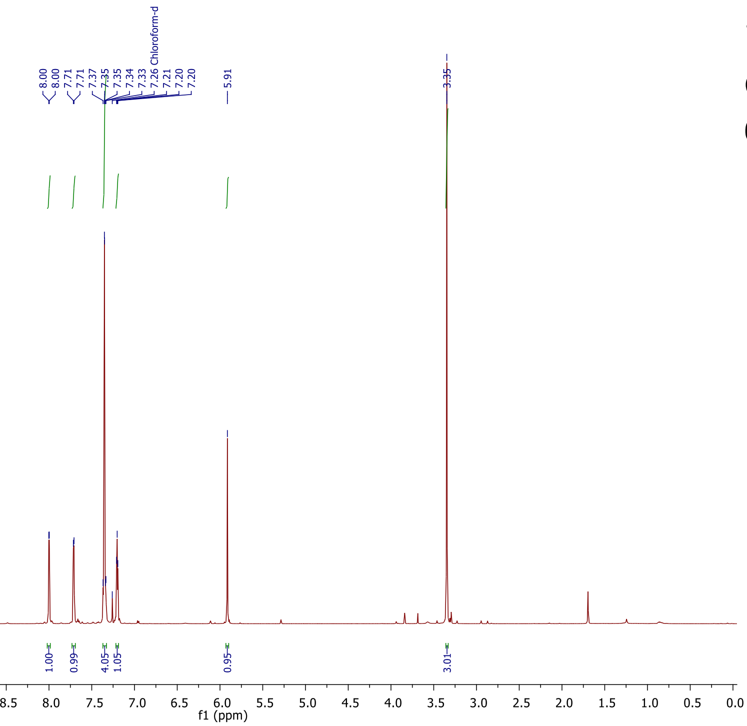

^{1}H
$CDCl_3$
600 MHz

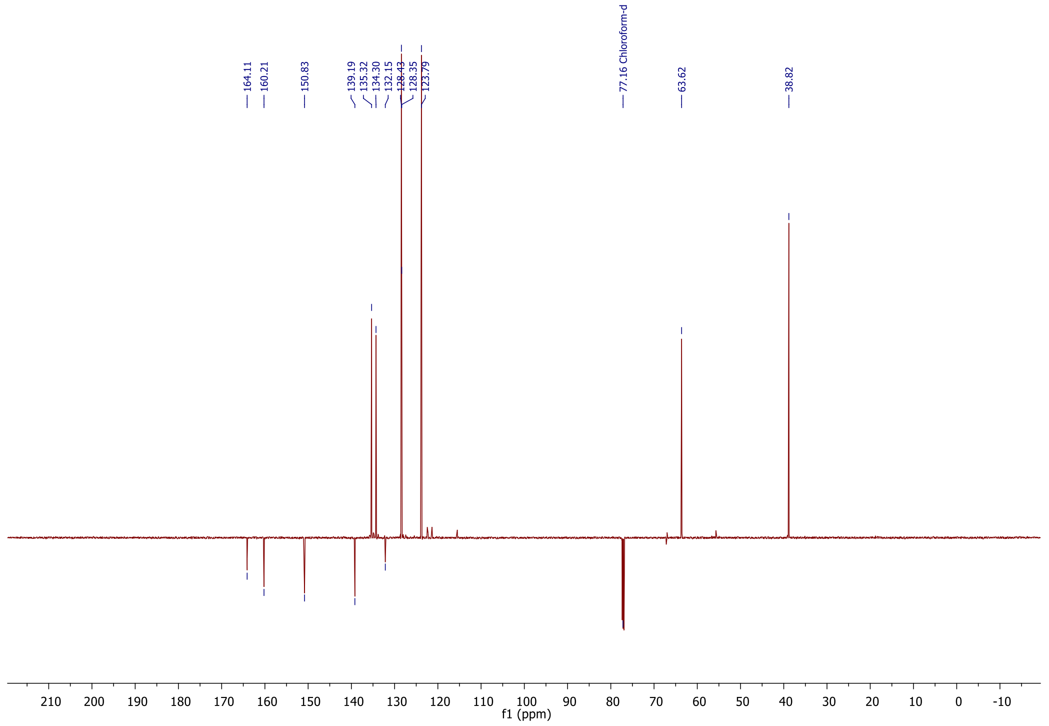

^{13}C
$CDCl_3$
150 MHz

5. Computational Details

The conformational space of all molecules has been initially searched using meta-dynamics simulations based on tight-binding quantum chemical calculations as implemented in CREST.[10,11]

The structures located with the CREST have then been subjected to PBE0-D3BJ/def2-SVP[12-16] geometry optimization. The nature of all stationary points (minima and transition states) was verified through the computation of the vibrational frequencies. The thermal corrections to the Gibbs free energy were combined with the single point energies calculated at the PBE0-D3BJ/def2-TZVP level of theory to yield Gibbs free energies ("G_{298}") at 298.15 K. All energies are reported in kcal mol^{-1}.

The polarizable continuum model (PCM) with SMD parameters[17,18] was applied to consider solvent (THF) effects for both geometries and energies. Free energies in solution have been corrected to a reference state of 1 mol l^{-1} at 298.15 K through the addition of RTln(24.46) = +7.925 kJ mol^{-1} to the gas phase (1 atm) free energies.

The DFT calculations have been performed with the Gaussian 16 program package[19].

6. References

1. J. Wen, A. Wu, P. Chen, J. Zhu, *Tetrahedron Lett*. **2015**, *56*, 5282-5286. (3a, 3h, 3j, 3m)
2. F. T. Wong, P. K. Patra, J. Seayad, Y. Zhang, J. Y. Ying, *Org*. *Lett*. **2008**, *10*, 2333-2336. (3d, 3e)
3. K. Matsumoto, M. Kato, S. Sakamoto, Y. Kikugawa, *J. Chem*. *Res*. **1995**, *1*, 34-35. (3i)
4. A. Bossum, S. Blechert, *Angew. Chem.* **1988**, *100*, 596-597. (3k)
5. N. Böge, S. Krüger, M. Schröder, C. Meier, *Synthesis*, **2007**, 24, 3907-3914. (3l)
6. K. N. Hojczyk, P. Feng, C. Zhan, M.-Y. Ngai, *Angew*. *Chem*. *Int*. *Ed*. **2014**, *53*, 14559-14563. (3p, r)
7. S. Shaaban, V. Tona, B. Peng, N. Maulide, *Angew. Chem. Int. Ed.*, **2017**, *56*, 10938-10941. (3q, 3t)
8. Santen Pharmaceutical Co., Ltd - EP2119703, 2009, A1 (3s)
9. I. Nakamura, M. Owada, T. Jo, M. Terada, *Org. Lett.* **2017**, *19*, 2194-2196.
10. P. Pracht, F. Bohle, S. Grimme, *Phys. Chem. Chem. Phys.* **2020**, *22*, DOI 10.1039/c9cp06869d.
11. S. Grimme, *J. Chem. Theory Comput.* **2019**, *15*, 2847–2862.
12. J. P. Perdew, K. Burke, M. Ernzerhof, *Phys. Rev. Lett.* **1996**, *77*, 3865–3868.
13. J. P. Perdew, K. Burke, M. Ernzerhof, *Phys. Rev. Lett.* **1997**, *78*, 1396.
14. C. Adamo, V. Barone, *J. Chem. Phys.* **1999**, *110*, 6158.
15. S. Grimme, J. Antony, S. Ehrlich, H. Krieg, *J. Chem. Phys.* **2010**, *132*, 154104.
16. S. Grimme, S. Ehrlich, L. Goerigk, *J. Comput. Chem.* **2011**, *32*, 1456–1465.
17. E. Cancès, B. Mennucci, J. Tomasi, *J. Chem. Phys.* **1997**, *107*, 3032–3041.
18. A. V. Marenich, C. J. Cramer, D. G. Truhlar, *J. Phys. Chem. B* **2009**, *113*, 6378–6396.
19. M. J. Frisch, G. W. Trucks, H. B. Schlegel, G. E. Scuseria, M. A. Robb, J. R. Cheeseman, G. Scalmani, V. Barone, B. Mennucci, G. A. Petersson, H. Nakatsuji, M. Caricato, X. Li, H. Hratchian, A. Izmaylov, J. Bloino, G. Zheng, J. Sonnenberg, M. Hada, M. Ehara, K. Toyota, R. Fukuda, J. Hasegawa, M. Ishida, T. Nakajima, Y. Honda, O. Kitao, H. Nakai, T. Vreven, J.

Montgomery, J. Peralta, F. Ogliaro, M. Bearpark, J. Heyd, E. Brothers, K. Kudin, V. Staroverov, R. Kobayashi, J. Normand, K. Raghavachari, A. Rendell, J. Burant, S. Iyengar, J. Tomasi, M. Cossi, N. Rega, J. Millam, M. Klene, J. Knox, J. Cross, V. Bakken, C. Adamo, J. Jaramillo, R. Gomperts, R. Stratmann, O. Yazyev, A. Austin, R. Cammi, C. Pomelli, J. Ochterski, R. Martin, K. Morokuma, V. Zakrzewski, G. Voth, P. Salvador, J. Dannenberg, S. Dapprich, A. Daniels, Farkas, J. Foresman, J. Ortiz, J. Cioslowski, D. Fox, **n.d.**
